# Liposomal formulation of a new antifungal hybrid compound provides protection against *Candida auris* in the *ex vivo* skin colonization model

**DOI:** 10.1128/aac.00955-23

**Published:** 2023-12-11

**Authors:** Anna Jaromin, Robert Zarnowski, Adam Markowski, Agnieszka Zagórska, Chad J. Johnson, Haniyeh Etezadi, Shinji Kihara, Pablo Mota-Santiago, Jeniel E. Nett, Ben J. Boyd, David R. Andes

**Affiliations:** 1 Department of Lipids and Liposomes, Faculty of Biotechnology, University of Wrocław, Wrocław, Poland; 2 Department of Medicine, School of Medicine & Public Health, University of Wisconsin-Madison, Madison, Wisconsin, USA; 3 Department of Medical Microbiology, School of Medicine & Public Health, University of Wisconsin-Madison, Madison, Wisconsin, USA; 4 Department of Medicinal Chemistry, Jagiellonian University Medical College, Cracow, Poland; 5 Department of Medicine, University of Wisconsin, Madison, Wisconsin, USA; 6 Department of Pharmacy, University of Copenhagen, Copenhagen, Denmark; 7 MAX IV Laboratory, Lund University, Lund, Sweden; 8 Department of Medical Microbiology and Immunology, University of Wisconsin, Madison, Wisconsin, USA; 9 Drug Delivery, Disposition and Dynamics, Monash Institute of Pharmaceutical Sciences, Monash University (Parkville Campus), Victoria, Australia; University Children's Hospital Münster, Münster, Germany

**Keywords:** *Candida auris*, biofilm, antifungal, proteomics, liposomes, fungal skin infection

## Abstract

The newly emerged pathogen, *Candida auris*, presents a serious threat to public health worldwide. This multidrug-resistant yeast often colonizes and persists on the skin of patients, can easily spread from person to person, and can cause life-threatening systemic infections. New antifungal therapies are therefore urgently needed to limit and control both superficial and systemic *C. auris* infections. In this study, we designed a novel antifungal agent, PQA-Az-13, that contains a combination of indazole, pyrrolidine, and arylpiperazine scaffolds substituted with a trifluoromethyl moiety. PQA-Az-13 demonstrated antifungal activity against biofilms of a set of 10 different *C. auris* clinical isolates, representing all four geographical clades distinguished within this species. This compound showed strong activity, with MIC values between 0.67 and 1.25 µg/mL. Cellular proteomics indicated that PQA-Az-13 partially or completely inhibited numerous enzymatic proteins in *C. auris* biofilms, particularly those involved in both amino acid biosynthesis and metabolism processes, as well as in general energy-producing processes. Due to its hydrophobic nature and limited aqueous solubility, PQA-Az-13 was encapsulated in cationic liposomes composed of soybean phosphatidylcholine (SPC), 1,2-dioleoyloxy-3-trimethylammonium-propane chloride (DOTAP), and *N*-(carbonyl-methoxypolyethylene glycol-2000)-1,2-distearoyl-*sn*-glycero-3-phosphoethanolamine, sodium salt (DSPE-PEG 2000), and characterized by biophysical and spectral techniques. These PQA-Az-13-loaded liposomes displayed a mean size of 76.4 nm, a positive charge of +45.0 mV, a high encapsulation efficiency of 97.2%, excellent stability, and no toxicity to normal human dermal fibroblasts. PQA-Az-13 liposomes demonstrated enhanced antifungal activity levels against both *C. auris* in *in vitro* biofilms and *ex vivo* skin colonization models. These initial results suggest that molecules like PQA-Az-13 warrant further study and development.

## INTRODUCTION

*C. auri*s was first identified in Japan in 2009 from the ear canal of a patient (1). Nowadays, the pathogen is considered as a threat to global public health, and it is the focus of intensive effort in many research centers around the world. However, even with appropriate antifungal treatment, mortality rates are high (2). The majority of *C. auris* isolates fall into major geographical clades: I (South Asian), II (East Asian), III (African), IV (South American), and V, recently identified in Iran (3 – 5). One hypothesis is that it may be the first new fungal disease emerging from human-induced climate change (6) but some aspects still need to be explained.

*C. auri*s has an unusual ability to persist on human skin and other surfaces, such as catheters, for a long time, and biofilm formation has been suggested as an important factor for the persistence of this organism (7). Moreover, a recent alarming study (8) reports that the emergence of the pan-drug resistance of *C. auris* to four major classes of antifungals implies that patients at high risk for the drug-resistant pathogen will soon need novel therapeutic strategies. Therefore, the identification and development of new candicidal agents able to overcome this emerging organism have become the highest priority.

**Fig 1:**
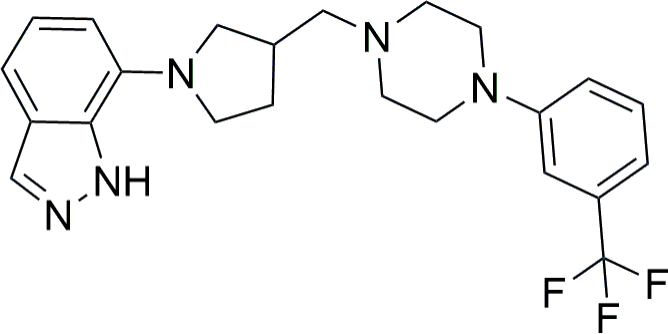
Chemical structure of PQA-Az-13 (7-(3-((4-(3-(trifluoromethyl)phenyl)piperazin-1-yl)methyl)pyrrolidin-1-yl)-1H-indazole).

To obtain an original compound active against *C. auris*, we designed and synthesized a new compound, PQA-Az-13 (Fig. 1), which contained in its structure a combination of indazole, pyrrolidine, and an arylpiperazine scaffold substituted with a trifluoromethyl moiety.

The unique chemical design adopted in the current study was prompted by promising data in the published literature showing significant antifungal activities of compounds containing these moieties in their molecules. For instance, Rodríguez-Villar and colleagues synthesized a series of 3-phenyl-1H-indazole derivatives which showed broad anticandidal activity against *C. albicans*, *C. glabrata*, and *C. tropicalis* strains (9). Additionally, a study conducted by Łukowska-Chojnacka et al. reports antifungal activity against *C. albicans* of tetrazole derivatives bearing a pyrrolidine scaffold, in which the most active compounds against biofilms *in vitro* also showed activity *in vivo* in the invertebrate model of disseminated candidiasis (10). In another study, aromatic-rich piperazines prevented *C. albicans* biofilm formation (11). By way of example, the antifungal activity of a novel quercetin derivative on *C. albicans* was improved by introduction of a trifluoromethyl group (12). Moreover, introduction of a trifluoromethyl group greatly improved the antifungal activity of coumarin thiazoles (13), as well as chalcone derivatives (14).

Although there has been significant effort and progress in developing nanotechnology-based approaches to combat *C. albicans*, highlighted in recent reviews (15 – 19), there are only a few examples, published recently, of successful formulations being active against *C. auris* biofilms. For example, interesting results were obtained using nanoparticles, especially silver. In one of the studies, silver nanoparticles showed a potent inhibitory activity on biofilm formation (IC_50_ of 0.06 ppm) and also in the case of preformed biofilms (IC_50_ of 0.48 ppm) (20). Also, promising antifungal activities were obtained in the case of other silver nanoparticles (21, 22). Another study performed by Cleare et al*.* demonstrated the effectiveness of fabricated nanoparticles which release N-acetylcysteine S-nitrosothiol and N-acetylcysteine and generated NO, on six *C. auris* strains (23). In parallel, Vazquez-Munoz et al. reported strong inhibitory activity of bismuth nanoparticles against different strains of *C. auris* (24). Moreover, Baldim et al. exploited nanostructured lipid carriers loaded with the essential oil of *Lippia sidoides* to combat *C. auris* (25). Also, an approach with the use of micafungin-loaded nanoemulsions *in vitro* and *in vivo* was recently reported (26).

Since the need for identification of new compounds active against *C. auris* has never been more pressing, the aim of this study was the synthesis and evaluation of an original antifungal agent against this emerging eminent threat to the human population worldwide. Considering its high hydrophobicity, a liposomal form of PQA-Az-13 was developed and characterized to enable its delivery, the effectiveness of which was verified against *C. auris* under *in vitro* conditions and in an *ex vivo* skin decolonization model. To the best of our knowledge, the activity of such a hybrid agent against this emerging fungus has not been reported, and neither has its liposomal form, nor its effectiveness, been described in the literature.

## MATERIALS AND METHODS

### Materials

SPC (Phospholipon 90G), DOTAP, and DSPE-PEG 2000 were purchased from Lipoid GmbH (Ludwigshafen, Germany). Chloroform and DMSO were from Archem (Kamieniec Wroclawski, Poland). MTT 3-(4,5-dimethyl-2-thiazolyl)-2,5-diphenyl-2H-tetrazolium bromide was from Sigma-Aldrich (Poznań, Poland). Dulbecco’s phosphate-buffered saline (DPBS), alpha-MEM, and the normal human dermal fibroblast cell line (NHDF) were obtained from Lonza (Walkersville, MD, USA, or Warsaw, Poland). L-Glutamine, phosphate-buffered saline, fetal bovine serum (FBS), and 100× antibiotic–antimycotic were purchased from Cytogene (Zgierz, Poland). *Candida auris* strains were stored in 15% glycerol frozen at −80°C and routinely maintained on yeast extract–peptone–dextrose (YPD) agar plates (1% yeast extract, 2% Bacto peptone, 2% dextrose, and 2% Bacto agar). Liquid cultures were grown in YPD broth (1% yeast extract, 2% Bacto peptone, and 2% dextrose) with shaking at 200 rpm at 30°C. For biofilm assays, strains were cultured in filter-sterilized Roswell Park Memorial Institute Medium 1640 (RPMI) containing 1% glucose and buffered with 4-morpholinepropanesulfonic acid adjusted to pH 7.0. *C. auris* strains were acquired from the Centers for Disease Control and Prevention (2).

### Organisms and inoculum

*C. auris* strains were cultured on yeast YPD agar plates. Cultures were propagated in YPD media overnight at 30°C at 200 rpm on an orbital shaker. Overnight cultures were counted and diluted down to the desired concentrations (1 × 10^4^ cells/mL for minimal inhibitory concentration (MIC) testing, 1 × 10^7^ cells/mL for the skin colonization assay, respectively). Skin inoculum was prepared in synthetic sweat media as previously described (27).

### Synthesis of PQA-Az-13

The detailed description of the synthesis of PQA-Az-13 is presented in the Supplementary Materials that accompany this publication.

### Antifungal susceptibility testing

Both free (1 mg/mL in DMSO, DMSO at final concentration of 0.5%) and liposomal forms of PQA-Az-13 were tested against *C. auris* isolates using the broth microdilution method employing RPMI 1640, in accordance with the standards established in the Clinical and Laboratory Standards Institute document, M27-A3 (28). Assays were established in 96-well round bottom plates and incubated at 35°C for 24 h, and MICs were determined in wells with complete growth inhibition (no viable growth relative to the growth of control). Untreated *C. auris* cells were used as controls. Two independent assays involving two technical replicates per isolate were performed in this study.

### Determination of effects on extracellular vesicle production

Extracellular vesicles (EVs) were measured directly in filter-sterilized post-culture supernatants. Extracellular vesicle samples were analyzed using a NanoSight NS300 nanoparticle tracking analyzer (Malvern) as described in reference (29). Briefly, EV samples were diluted in phosphate-buffered saline (PBS) to a final volume of 1 mL and pretested to obtain an ideal 30–100 particles per frame rate. The following settings were applied: the camera level was increased to 16 and camera gain to 2, until tested images were optimized, and nanoparticles were distinctly visible without exceeding particle signal saturation. Each measurement consisted of five 1-min videos with a delay of 5 s between sample introduction and the start of the first measurement. For detection threshold analysis, the counts were limited to 10–100 red crosses and no more than 5–7 blue crosses. Acquired data were analyzed using the NanoSight Software NTA 3.4 Build 3.4.003. At least 1,000 events in total were tracked per sample in order to minimize data skewing based on single large particles.

### *C. auris* biofilm cellular proteomics and functional mapping

Mass spectrometry-based proteomics was performed at the Mass Spectrometry Facility Core, Biotechnology Center, University of Wisconsin–Madison. *C. auris* B11203 biofilms were grown in 6-well plates for 48 h, and PQA-Az-13 was applied at a subinhibitory concentration of 0.5 µg/mL after 24 h of incubation. Biofilms were then removed from wells with a sterile spatula and harvested in 1 mL sterile water per individual well. The aliquots were combined in a 15-mL Falcon tube, and the biofilm biomass was sonicated in a water bath sonicator for 20 min, followed by centrifugation at 2,880 × *g* at 4°C for 20 min. Then, the supernatant was decanted, and pelleted fungal biofilm cells were washed twice with PBS containing cOmplete Mini Protease Inhibitor Cocktail (Roche) at the concentration recommended by the supplier. Such prepared fungal cells were transferred to 2-mL micro-tubes (Sarstedt) containing glass beads and broken open with in a bead beater (Mini-Beadbeater, Biospec Products) at full speed for 1-min increments for a total of 5 min, with 1-min incubations on ice in between bead-beating repeats. The tubes were then centrifuged at 4,000 × *g* to pellet cell debris, and the separated supernatant was filter sterilized through a 0.2-µm syringe filter and transferred to a fresh Eppendorf tube. Protein amounts in such prepared cell lysates were determined using a NanoDrop spectrophotometer (Thermo Scientific). Sample preparation and the subsequent proteomics studies were performed as described elsewhere (29). Two hundred micrograms of cellular proteins were extracted by precipitation with 15% TCA/60% acetone and then incubated at −20°C for 30 min. Pelleted proteins were resolubilized and denatured in 10 µL of 8 M urea in 100 mM NH_4_HCO_3_ for 10 min and then diluted to 60 µL for tryptic digestion “in liquid” with the following reagents: 3 µL of 25 mM DTT, 4.5 µL of acetonitrile, 36.2 µL of 25 mM NH_4_HCO_3_, 0.3 µL of 1M Tris-HCl, and 6 µL of 100 ng/µL Trypsin Gold solution in 25 mM NH_4_HCO_3_ (Promega). Digestion was conducted in two stages, first overnight at 37°C, and then, additional 4-µL volumes of trypsin solution were added and the mixtures were incubated at 42°C for an additional 2 h. The reaction was terminated by acidification with 2.5% TFA to a final concentration of 0.3% and then centrifuged at 16,000 × *g* for 10 min. Trypsin-generated peptides were analyzed by nanoLC-MS/MS using the Agilent 1100 nanoflow HPLC system (Agilent) connected to a hybrid linear ion trap-orbitrap mass spectrometer (LTQ-Orbitrap, Thermo Fisher Scientific) equipped with a nanoelectrospray ion source. Capillary HPLC was performed using an in-house fabricated column with an integrated electrospray emitter (30). Sample loading and desalting were achieved using a trapping column in line with the autosampler (Zorbax 300 SB-C18, 5 µm, 5 × 0.3 mm, Agilent). The LTQ-Orbitrap was set to acquire MS/MS spectra in a data-dependent mode as follows: MS survey scans from 300 to 2,000 m/z were collected in profile mode with a resolving power of 100,000. MS/MS spectra were collected on the five most abundant signals in each survey scan. Dynamic exclusion was employed to increase the dynamic range and maximize peptide identifications. Raw MS/MS data were searched against a concatenated *C. auris* amino acid sequence database using an in-house MASCOT search engine (31). Identified proteins were further annotated and filtered to 1.5% peptide and 0.1% protein false-discovery rate with Scaffold Q+ version 4.11.1 (Proteome Software Inc.) using the protein prophet algorithm (32). Mascot was searched with a fragment ion mass tolerance of 0.60 Da and a parent ion tolerance of 10.0 ppm. Carbamidomethyl of cysteine and j+138 of leucine/isoleucine indecision were specified in Mascot as fixed modifications. The deamination of asparagine and glutamine and the oxidation of methionine were specified in Mascot as variable modifications.

The cellular proteomes of *C. auris* were analyzed using both Kyoto Encyclopedia of Genes and Genomes (KEGG) pathway maps and the BRITE hierarchies databases deposited at the KEGG. BLASTP-based mapped protein orthologies between *C. auris* and *C. albicans* species were obtained from the Candida Genome Database, and for each protein detected in this study, a KEGG Ontology ID was assigned, while retaining related specific pathway and superpathway membership information, as determined by KEGG/BRITE assignments. The visualization of relative quantities of biofilm cellular proteins as ratios of PQA-Az-13-treated vs. untreated controls was done using KEGG/BRITE protein functional categorization. Based on this hierarchical classification scheme, Voronoi treemaps were constructed using Paver (v. 2.1.9, DECODON Software UG). This approach divides screen space according to hierarchical levels in which the main functional categories determine screen sections on the first level, subsidiary categories on the second level, and so forth. The polygonic cells of the deepest level represent functionally classified proteins and are colored according to the relative abundance of each protein that was determined, based on the total counts of the corresponding trypsin-digested peptides.

### Preparation of liposomes with PQA-Az-13

Liposomes were prepared by a thin-film hydration method followed by a sonication step. Briefly, SPC:DOTAP:DSPE-PEG 2000 at molar ratios of 79.5:20:0.5, dissolved in chloroform, were mixed (total lipid content 20 mg). Then, 1.0 mg of PQA-Az-13 in chloroform was added (1:20 wt ratio). Subsequently, all components were thoroughly mixed, and chloroform was evaporated under a stream of nitrogen. The obtained dry, thin lipid film was hydrated with 1.0 mL of Milli-Q water and placed in an ultrasound bath (Sonic-10, Polsonic, Warsaw, Poland) at 50°C for 1.5 min. Liposomes were further ultrasonicated (Vibra Cell VCX130, Sonics & Materials Inc., Newtown, CT, USA) for 1 min at 30% amplitude at room temperature. The obtained liposomal suspension was centrifuged at 13,000 rpm for 2 min to separate the unencapsulated PQA-Az-13. Finally, the resulting formulation was stored at 4°C until further use. Control liposomes were prepared using the method described above but without addition of PQA-Az-13.

### Analysis of liposome structure using cryogenic transmission electron microscopy

Morphological analysis was performed using a Tecnai G2 20 TWIN transmission electron microscope (FEI, Hillsboro, OR, USA) as described in (33).

### Analysis of liposome structure using small-angle X-ray scattering

The small-angle X-ray scattering (SAXS)/wide angle X-ray scattering (WAXS) experiments were conducted in air and room temperature in transmission mode with an X-ray energy of 12.4 keV at the CoSAXS beamline at the 3-GeV ring at the MAX IV (34) laboratory. Data were acquired with a hybrid pixel X-ray detector, namely, an Eiger2 4M detector, and an acquisition time of 0.1 s per measurement. The sample-to-detector distance was determined using silver behenate (AgBeh) as calibrant resulting in a scattering vector q range of 0.005 to 0.5 Å ^−1^ ( q defined as $q=\frac{4\pi }{\lambda }∙\sin\theta$ , where 2θ is the scattering angle). Thin-wall quartz capillaries were filled with liposomes, loaded on a capillary holder mounted on high-precision vertical and horizontal translation stages. Each sample was measured 10 mm along the capillary, from the bottom, with a step size of 0.5 mm. After normalization to exposure time and masking of invalid pixels, data reduction was conducted using the Python-based azint library developed at MAX IV Laboratory (35). Measurements with low acquisition times and at various positions along the capillary were collected to minimize any potential radiation damage effect. The compendium of reduced data was averaged using a correlation mapping to disregard variations in concentration or the presence of artifacts such as air bubbles for each sample (36). The analysis of the SAXS data was conducted with SasView (version 5.05). The SAXS patterns were analyzed using a lamellar head and tail group model (37) to estimate the thickness of the lipid head and tail groups, with the Levenberg–Marquardt fitting algorithm.

### Analysis of size and zeta potential

Size, polydispersity index (PdI), and zeta potential of the liposomes were determined using dynamic light scattering (DLS) on a Malvern NanoZS instrument (Malvern Industries, Malvern, UK). Measurements of samples composed of 10 µL of liposomes and 990 µL of Milli-Q water were made at 25°C and in triplicate.

### Encapsulation efficiency

Briefly, the standard solution of PQA-Az-13 was prepared by dissolving 1 mg of the compound (accurately weighed) in 1 mL of methanol. This stock solution was used to prepare further dilutions of standard solutions. The standard solutions were measured at 253 nm, using a microplate reader (EnSpire Multimode; PerkinElmer, Waltham, MA, USA). The calibration curve was constructed by plotting absorbance values (*A*) against concentrations of PQA-Az-13 (c). The regression equation of the linear calibration graph was calculated as *A* = 1.116*c* + 0.89 (*R* ^2^ = 0.9836). Before analysis, a 100-µL aliquot of PQA-Az-13 liposomes was dissolved in 900 µL of methanol for disruption of liposomal structure. The encapsulation efficiency was calculated according to the following equation:


$$Encapsulation\;efficiency\;(\%)=\frac{L}{T}\times 100$$

where *L* is the amount of PQA-Az-13 measured in the liposomal suspension and *T* is the total amount of PQA-Az-13 used in the preparation of liposomes, *n* = 6.

### Stability and retention analysis

The size, PdI, and zeta potential of liposomes with PQA-Az-13 were measured immediately after preparation and during storage at 4°C for 7, 14, and 21 days. Additionally, at these intervals, liposomes were vortexed, centrifuged at 13,000 rpm for 2 min, and analyzed to determine the PQA-Az-13 content as described below.

### Cytotoxicity test

The evaluation of the cytotoxicity of PQA-Az-13 liposomes was performed *in vitro* on normal human dermal fibroblast cells using the MTT assay (38) as described in references (39, 40).

### Treatment of porcine skin biofilm

Decolonization of porcine skin was carried out as previously described (40) with slight modifications. Porcine skin samples were acquired under protocols approved by the University of Wisconsin–Madison Institutional Animal Care and Use Committee in compliance with the National Institutes of Health and United States Department of Agriculture guidelines. Skin biopsies (12 mm) were obtained and prepared as previously described (27, 41). *C auris* (10 µL, 1 × 10^7^ /mL in synthetic sweat media) was placed on the prepared skin surface and incubated for 24 h in a 37°C incubator with 5% CO_2_. After 24 h, skins were blotted dry with a sterile cotton applicator, and either they were left untreated (no treatment control) or PQA-Az-13-liposomes or control liposome treatments were applied with a saturated sterile applicator. Skins were then incubated for 1 h at 37°C and then dabbed dry with sterile applicators. Skins were placed in 10 mL DPBS, vortexed on high for 1 min, and then diluted and plated on YPD + chloramphenicol (25 µg/mL) plates. Plates were incubated overnight at 30°C, and colonies were enumerated (limit of detection 10^3^ cells).

### Histopathology

After treatment, as described above, skin samples were gently immersed in 10% neutral-buffered formalin for at least 24 h. Following fixation, skin tissues were sent to the University of Wisconsin surgery histology core facility, where they were bisected and embedded midline down in paraffin. Ten-micron sections were cut and stained with hematoxylin and eosin. Slides were imaged on a Nikon Ti2 microscope using NIS Elements software.

### Statistical analysis

Data sets of equal sample size were analyzed using non-parametric Kruskal–Wallis one-way analysis of variance with post hoc uncorrected Dunn’s multiple comparison test without prior elimination of outliers. Data were processed with GraphPad Prism 9 for Windows 64-bit [version 9.5.0 (730)].

## RESULTS AND DISCUSSION

### Properties of PQA-Az-13

As a result of well-established synthetic pathways for alkylation or N-arylation reactions, PQA-Az-13 was successfully obtained for which a molecular property prediction was performed (Table S1). It is worth noting that this structure fulfills all criteria established in Lipinski’s rule of five, namely, log *P*_o/w_ (octanol/water partition coefficient) < 5, molecular weight (MW) < 500, H-bond acceptors < 10, H-bond donors < 5, and the Veber rule criteria, such as rotatable bonds (RB), with preferably an RB < 10, and a topological polar surface area < 140 A^2^. Additionally, this compound does not contain substructural features recognized as pan-assay interference compounds, as demonstrated by the application of the SwissADME tool. Therefore, it could be concluded that the new synthesized structure shows drug-like properties and possibly favorable membrane permeability and bioavailability.

### Antifungal activity of PQA-Az-13 against *C. auris*

PQA-Az-13 was further tested for its antifungal efficacy against 10 different clinical isolates of *C. auris*, including each of the common clades. The determined MIC values (the lowest concentration at which no growth was observed) revealed susceptibility of the tested strains of pathogen toward this compound in a very narrow concentration range 0.67–1.25 µg/mL (Table 1). It is clear that this agent has a strong activity against *C. auris*, especially when the obtained results are compared with the data available for fluconazole (42). Additionally, the compound had lower MIC values for six strains of *C. auris* (B11801, B11203, B11219, B11211, B11104, and B11785) than amphotericin B.”

**TABLE 1:** Susceptibility of clinical *C. auris* strains

Strain	Country of origin	MIC (µg/mL)			
		PQA-Az-13	Fluconazole^*a*^	Amphotericin B^*a*^	Rezafungin^*b*^
B11804	Colombia	1.25	2	0.5	–^*c*^
B11220	Japan	1.25	4	0.38	0.06
B11221	South Africa	1.25	128	0.38	–
B11801	Colombia	0.67	16	6	–
B11203	India	0.67	256	4	–
B11219	India	1.25	>256	3	–
B11211	India	0.67	>256	1.5	2
B11104	Pakistan	0.67	>256	1	–
B11799	Colombia	1.25	16	0.5	0.25
B11785	Colombia	1.25	8	1.5	0.125

^*a*^ Data from reference (42).

^*b*^ Data from reference (43).

^*c*^ "-" data unavailable.

However, the exhibited MIC values were, with one exception (B11211), higher than those found for rezafungin, which is a novel echinocandin being developed for the treatment and prevention of invasive fungal infections (43). It is also worth mentioning that the determined antifungal activity of PQA-Az-13 was much better than the recently published data for azobenzene (44) or thiazole derivatives (45), for Schiff bases of sulphonamides (46), for 4-dialkoxynaphthalen-2-acyl imidazolium salts (47), and for derivatives of the antimalarial drug mefloquine (48). Thus, our results are particularly interesting, especially in the context that, for the pan-resistant *C. auris* strain, B11211, a MIC value of 0.67 µg/mL was obtained for PQA-Az-13, whereas MIC values of >256 µg/mL for fluconazole and 1.5 µg/mL for amphotericin B were reported in reference (42) and a MIC of 2 µg/mL reported for rezafungin (43), confirming the superior antifungal properties of the synthesized structure and its potential application to drug-resistant fungi. Experiments reported in this work were conducted at 35°C, which closely mimics thermal skin conditions occurring during *C. auris* colonization and biofilm development.

### Mode of action of PQA-Az-13 against *C. auris* cells

**Fig 2:**
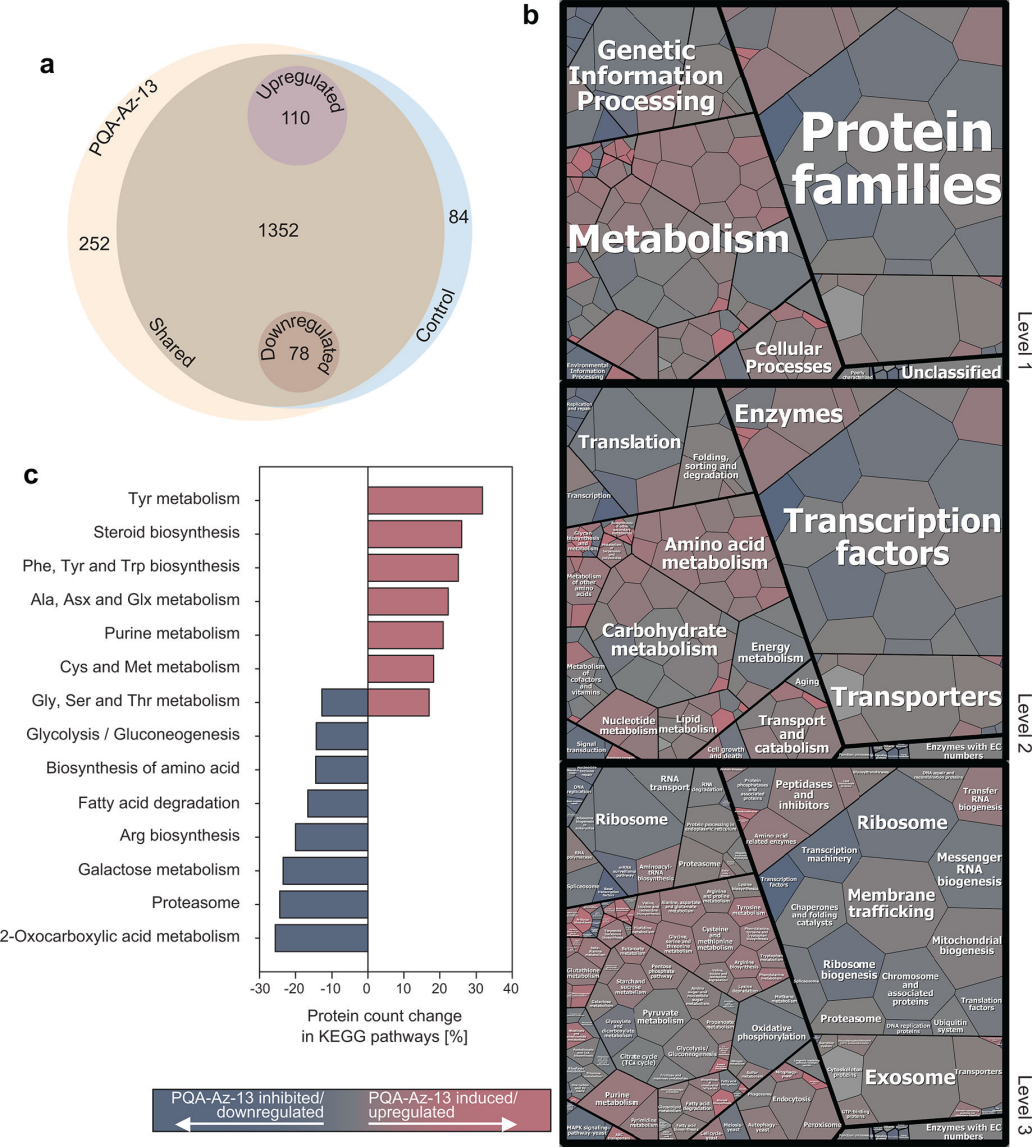
Cellular proteomics reveal the mode of action of PQA-Az-13 against *C. auris*. (**a**) A Venn diagram depicting both qualitative and quantitative changes in the *C. auris* cellular proteome after treatment with PQA-Az-13. Both control and compound-treated cells shared a pool of 1,352 proteins (dark-vanilla brown), out of which 110 (silver pink) and 78 (mocha orange) were upregulated or downregulated in the presence of the tested compound, respectively. PQA-Az-13 completely inhibited a pool of 84 proteins (Columbia blue) that were identified only in control cells, whereas another pool of 252 proteins (very pale orange) were induced and found only in the drug-treated cells. (b) KEGG/BRITE-based functional mapping of the *C. auris* cellular proteome in the presence of PQA-Az-13. Clusters of drug-inhibited/downregulated and drug-induced/upregulated proteins are shown in a color gradient ramp, starting with rose-gold pink-red and reaching dark-electric blue. Proteins that were not affected by PQA-Az-13 are indicated in trolley grey. (c) Graphical summary of main changes in protein counts in the compound-modulated KEGG pathways in *C. auris*. PQA-Az-13 caused an inhibitory effect upon amino acid biosynthesis and metabolism processes, as well as in general energy producing processes (such as fatty acid degradation and glycolysis/gluconeogenesis). Possibly, because of metabolic inhibition compensation to drug action, metabolic pathways responsible for aromatic amino acid biosynthesis or steroid biosynthesis were induced.

The analysis of cellular proteomics in control and PQA-Az-13-treated *C. auris* cells led to the identification of a total of 1,688 proteins (Fig. 2; Table SM2). Both the compared cellular extracts contained a total of 1,352 shared proteins, of which 110 and 78 were up- and downregulated in the presence of the tested compound, respectively. In addition, the untreated control contained 84 unique proteins, whereas 252 unique proteins were identified in the treated cells. Thus, PQA-Az-13 induced or increased the production of 363 proteins, while decreasing or completely abolishing the synthesis of 162 proteins, respectively. These proteins were further mapped functionally based on their assignments in the KEGG and Brite databases (49) (Fig. 2b).

Theoretically, any drug targeting cellular metabolism causes a decrease in level(s) of drug target(s) and an accumulation of functionally impaired enzymatic proteins, due to changes in cellular substrates–products equilibria. The treatment of *C. auris* with PQA-Az-13 negatively impacted several proteins involved in transcription, proteins associated with chromosomes, proteins involved in ribosome biogenesis, transcription factors, and, among others, messenger RNA biogenesis. Brite-based functional classification of proteins strongly suggested that PQA-Az-13 impairs both the intercellular exosome and membrane trafficking pathways (Table 2).

**TABLE 2:** Major *C. auris* biofilm proteins modulated by PQA-Az-13

Protein level	KEGG/Brite-assigned function	Protein name
Decreased	Transcription machinery	Rpb11, B9J08_003412, Med11, Ngg1, B9J08_004752, B9J08_003128, B9J08_003058, Ssu72, Nab3, Ess1, B9J08_004585, Rtf1, Lys12, Maf1
	Chromosome and associated proteins	Nap1, Ngg1, B9J08_003412, B9J08_004752, B9J08_000541, B9J08_001735, Sin3, Rts1, Dpb4, B9J08_005192, B9J08_003058, B9J08_003128
	Ribosome biogenesis	B9J08_005358, B9J08_004502, Cic1, B9J08_001941, Pwp1, B9J08_005126, Ltv1, B9J08_001560, B9J08_000910, B9J08_002602
	Transcription factors	Tye7, Msn4, Bcr1, Cas5, Cta5, B9J08_002959, Rfg1, Hap5
	Messenger RNA biogenesis	Ssu72, B9J08_004752, B9J08_000310, Rts1, She3, Ssd1, U4/U6-U5 snRNP complex subunit
	Intercellular exosomes	Tdh3, Cdc19, Gpa2, Arf1, Chc1, Arp2, Arp3, Phb12, Tub1, Tcp1, B9J08_004407, Cct5, Cct6, Cct7, Pph21, Imh3, Gsp1, Sec21, Tub2, Arg1, Cys3, Fbp1, Gal1, Glc3, Cwh41, Mis12, Asc1, B9J08_001902, B9J08_004825, B9J08_002703, Phb2, Sec26, Sec27
	Membrane trafficking	Pld1, Chc1, Sla4, Arf1, B9J08_002589, Vps1, Sec13, Sec23, Erp5, B9J08_004361, B9J08_001902, Sec26, Sec27, Sec21, B9J08_004846, Atg1, B9J08_000779, B9J08_000251, Glk1, Pex6, Tdh3, Cdc19, Pph21, Arc35, Arp2, Arp3
Increased	Tyrosine metabolism	Uga2, Adh2, Amo2, B9J08_002737, His5, Aro9, B9J08_002703
	Steroid biosynthesis	Erg1, Erg9, Erg6, Erg26, Erg11, Erg5
	Phenylalanine, tyrosine, and tryptophan biosynthesis	His5, Aro9, Trp5, Trp2, Aro2
	Alanine, aspartate, and glutamate metabolism	Uga2, Ade4, B9J08_002863, B9J08_002737, Alt1, Cpa1, Gdh3, Arg1, Cpa2, Ade13, Ura2
	Purine metabolism	Ade4, Gua1, Met3, B9J08_005520, B9J08_001315, Prs1, B9J08_000825, B9J08_001217, Imh3, Rnr22, Prs5, Pnp1, Ade2, Ysa1, B9J08_003573, B9J08_001298, Pgm2, Ade6, Ade13
	Cysteine and methionine metabolism	Sam4, Sam2, B9J08_001645, B9J08_002822, Bat21, Meu1, B9J08_000042, Hom3, Cys3, Gsh2
	Glycine, serine, and threonine metabolism	Gcv2, Cly1, Ilv1, Amo2, B9J08_000042, Dao2, Hom3, Ifg3, Thr4, Shm2, B9J08_001967, Cys3
	Regulation of biofilm formation	Flo8, Bcr1, Gap4, B9J08_000099, Ywp1, Ifh1, Tye7, Cas5
	Fungal cell adhesion	Mss11, Bcr1, Ywp1, Cas5
	Cell wall organization	Zcf21, Bcr1, Ywp1, Rfg1, Atc1, Rca1, Snf7, Ssd1, Cas5, Cta8
	Intracellular and extracellular vesicle trafficking	Plb1, Plb4.5, Uso1, Gap4, Ywp1, Ena21, Snf7

Among the proteins either induced or upregulated by this compound, a total of 142 proteins involved in metabolism were classified in the KEGG database, of which 80 were predicted to participate in the biosynthesis of secondary metabolites. A large pool of 36 proteins was also classified into the biosynthesis of the amino acid cluster, while two other sets consisting of 20 proteins each were included into carbon metabolism and biosynthesis of cofactors. Further functional enrichment analysis pinpointed a handful number of enzymatic clusters, which were enhanced in the presence of PQA-Az-13. These groups included proteins participating in tyrosine metabolism; steroid biosynthesis; phenylalanine, tyrosine, and tryptophan biosynthesis; alanine, aspartate, and glutamate metabolism; purine metabolism; cysteine and methionine metabolism; and glycine, serine, and threonine metabolism (Table 2).

PQA-Az-13 negatively impacted numerous proteins functionally associated with fungal cell and biofilm biology including genes participating in the regulation of biofilm, fungal cell adhesion, cell wall organization, and intracellular and extracellular vesicle trafficking (Table 2).

**Fig 3:**
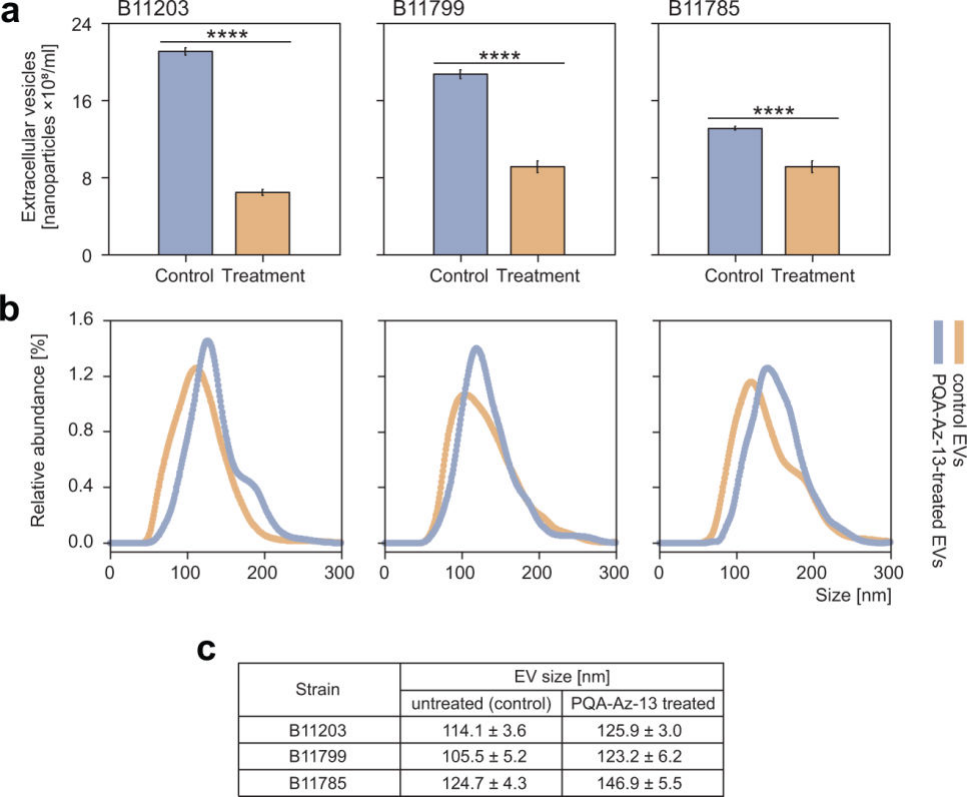
Effects of PQA-Az-13 on concentration (**a**), size distribution (**b**), and size (**c**) of EVs derived from the B11203, B11799, and B11785 strains of *C. auris*; *n* = 3 biological and 6 technical per biological.

These results encouraged us to further explore the possible effects of PQA-Az-13 on the size and concentration of extracellular vesicles produced during biofilm growth. To assess the impact of the compound on vesicle production, we selected representative strains of *C. auris*, namely, B11203, B11799, and B11785. The results, summarized in Fig. 3, clearly indicate that this compound strongly influenced this process in all tested strains. Firstly, the size of the produced vesicles increased, even by 22 nm for the B11785 strain. This observation is consistent with the results obtained for caspofungin, where also an increase of the size of EVs upon drug treatment was recorded (50). Secondly, their concentration decreased significantly at the same time, especially in the case of the B11203 and B11799 strains. This is a different phenomenon compared with the response observed to caspofungin treatment, where an increased amount of EVs was observed (50). On this basis, it can be concluded that PQA-Az-13 induced the production of a smaller number of EVs than in the case of the control, but at the same time, they were at least 10% larger by size. However, the exact cause of this phenomenon is unknown and requires further research to fully explain it, especially the decreased release of EVs and the possible effects on vesicle content.

### Liposomal formulation of PQA-Az-13

Taking into account that *Candida* carries a negative surface charge (51), we designed positively charged cationic liposomes in our research so as to strengthen the adhesion of liposomes to the surface of the pathogen. To prepare a liposomal formulation of PQA-Az-13, we used phosphatidylcholine from soybean, which is commonly used in drug delivery nanosystems (52), and a cationic lipid, DOTAP, in order to obtain positively charged liposomes, with the addition of DSPE-PEG 2000 to gain a steric stabilization. Liposomes containing these components were prepared by the lipid thin film hydration method, followed by sonication. The average hydrodynamic diameter of the liposomes, determined by the DLS technique, was 76.4 ± 1.8 nm (Table 3). Another analyzed parameter, PdI, which ranges from 0.0 to 1.0 and describes the degree of non-uniformity of the size distribution of particles, was 0.25 ± 0.02. Since the PdI value of ≤0.3 indicates a homogenous population of liposomes (53), the obtained value was, therefore, satisfactory. Also, very high positive values of zeta potential were detected for both control liposomes (+53.3 ± 1.1 mV) and PQA-Az-13-containing liposomes (+45.0 ± 1.7 mV), indicating a high colloidal stability. Moreover, high encapsulation efficiency (97.2% ± 5.0 %) testifies to the almost complete enclosure of the compound in the liposome structure.

**TABLE 3:** Characterization of control and PQA-Az-13-loaded liposomes

	Size (nm)	PdI	Zeta potential (mV)	Encapsulation efficiency (%)
Control liposomes	87.3 ± 2.1	0.27 ± 0.02	+53.3 ± 1.1	–
PQA-Az-13 liposomes	76.4 ± 1.8	0.25 ± 0.02	+45.0 ± 1.7	97.2 ± 5.0

**Fig 4:**
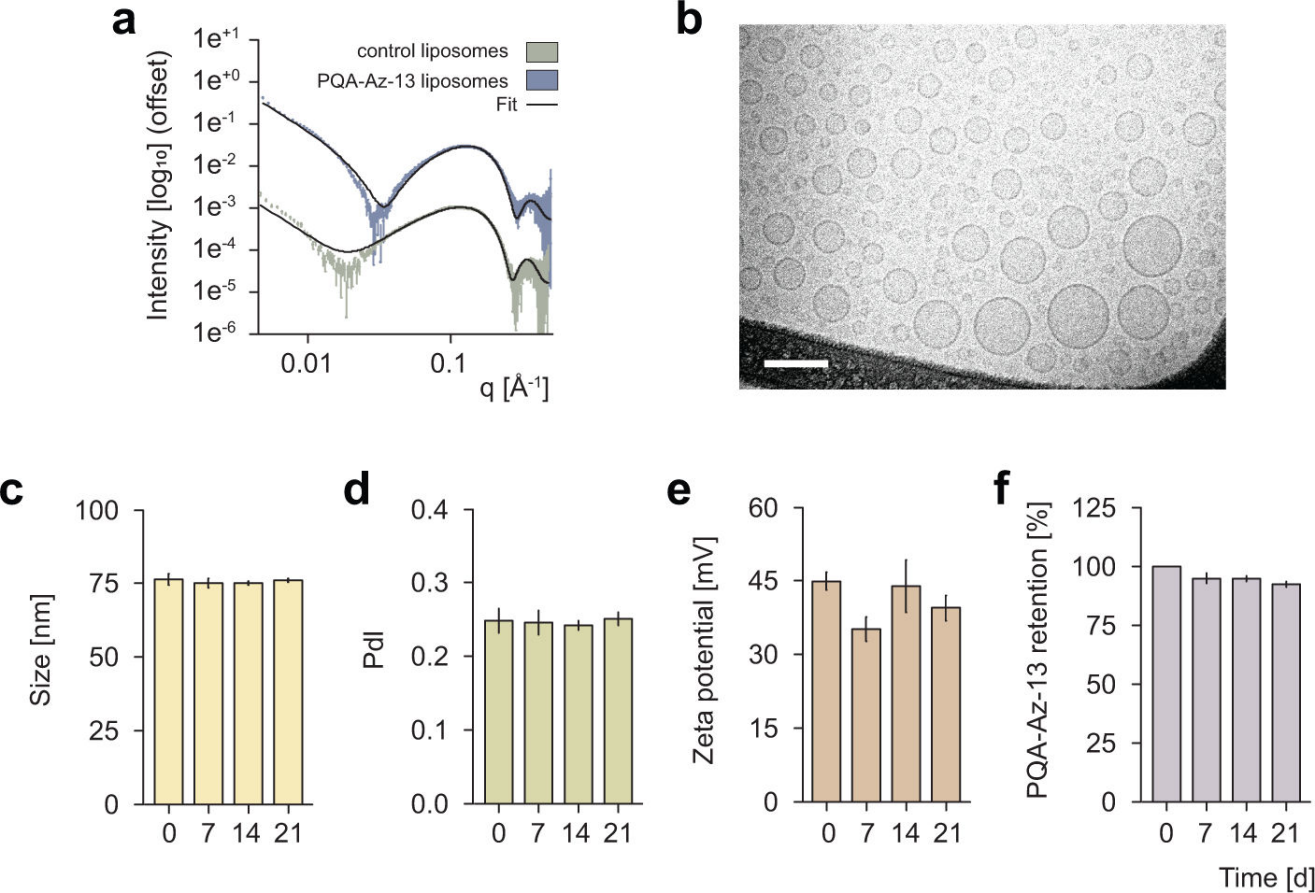
X-ray scattering intensity of the liposomes with and without PQA-Az-13 (data were fitted using a lamellar phase with head and tail group models (37) (**a**) and cryo-TEM (**b**) analysis of PQA-Az-13-loaded liposomes (scale bars represent 100 nm). Stability of PQA-Az-13 liposomes during a 21-day period on storage at 4°C—changes in hydrodynamic size (**c**), PdI (**d**), and zeta potential (**e**). Retention analysis of PQA-Az-13 liposomes (**f**).

The morphology of the developed liposomal formulation was further characterized by SAXS using a synchrotron source (Fig. 4a). The scattering patterns resembled the characteristic bilayer structure, supported by the fit to a previously reported liposomal scattering model (37). While the bilayer structure was retained with the introduction of PQA-Az-13, a minor structural alteration was observed, notably in the expansion of the hydrophilic headgroup of the lipid layer from 10.6 to 11.5 Å, along with a small thinning of the hydrophobic tail layer from 11.4 to 10.7 Å (fitting parameters are shown in Table SM3). Although the cause of this thickness variation remains unidentified and outside the scope of this work, it is possible that the introduction of the molecules altered the fluidity of the membrane and the tail ordering within the bilayer, which affected the occupied volume by each lipid layer.

Cryo-TEM imaging was also carried out, presented in Fig. 4b, which confirmed the presence of spherical vesicles. Additionally, the stability of the developed liposomes upon storage was determined. Measurements of size, PdI, and zeta potential were performed periodically for 21 days, as shown in Fig. 4c through e, and showed that the obtained formulation was very stable. Additionally, no disadvantageous processes, such as precipitation of the compound, were evident during the storage time and PQA-Az-13 retention was at the level of 92.4% after 21 days (Fig. 4f).

**Fig 5:**
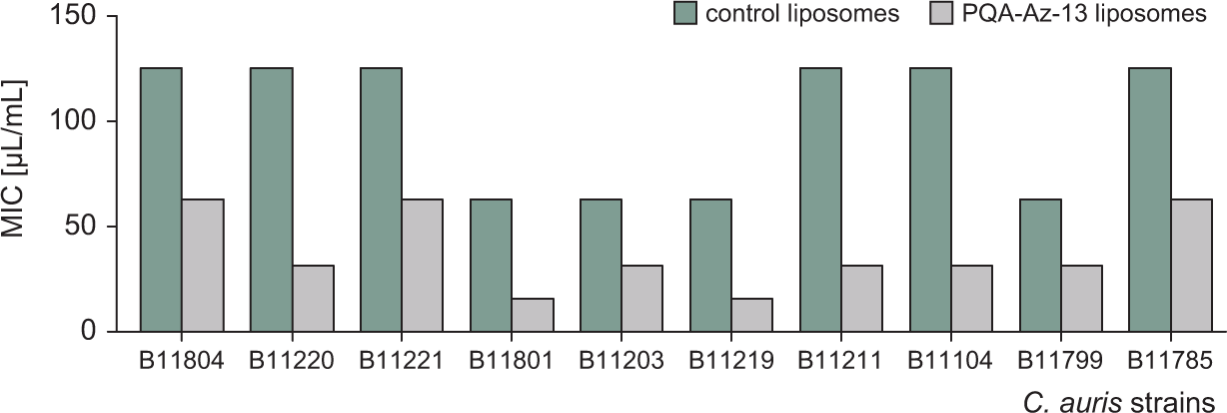
MICs for control liposomes and PQA-Az-13 liposomes against clinical *C. auris* strains.

The susceptibility of 10 *C*. *auris* strains to PQA-Az-13 liposomes as well as to control liposomes was then tested. The MIC values for loaded liposomes varied in the range of 15.6–62.5 µL/mL, which, taking into account the results from the encapsulation efficiency, corresponds to a PQA-Az-13 concentration of 15.2–60.7 µg/mL. Interestingly, control liposomes were also active but at higher concentrations 62.5–125 µL/mL (Fig. 5). Significantly, the values obtained for liposomes with PQA-Az-13 were at least 2–4 times lower than for the control liposomes, which indicates better activity of PQA-Az-13 in this developed lipid system. Although the effectiveness is not as great as for AmBisome, a liposomal preparation of amphotericin B (MIC range 0.25–2 µg/mL) (54), they are, however, at a similar level as those obtained for silver nanoparticles tested against *C. auris* MRL6057 (MIC 50.0 µg/mL) (22).

**Fig 6:**
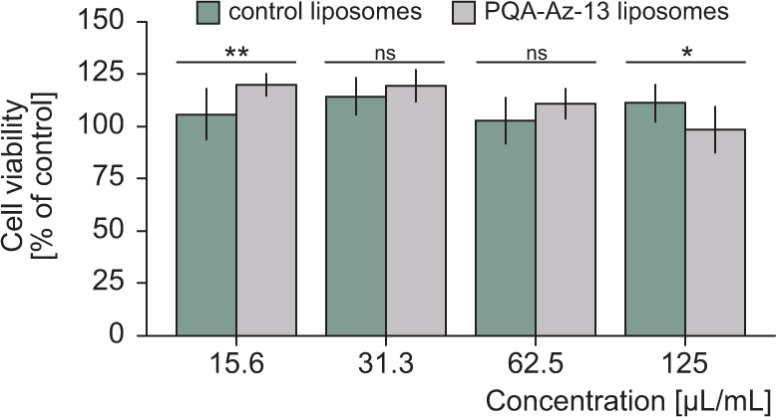
Viability of NHDF cells after 24 h of incubation with different concentrations of liposomes using MTT assay.

Cytotoxicity of these formulations is an important consideration; consequently, the *in vitro* cytotoxicity of the developed formulation was studied. We conducted this research with the use of normal human dermal fibroblast cells since, ultimately, the liposomes would be applied on the skin. As shown in Fig. 6, both control and test liposomes tested at the same concentrations as the MICs do not show any cytotoxicity (usually indicated by a viability level of <80%) and are, therefore, safe for application in the vicinity of this type of cells.

**Fig 7:**
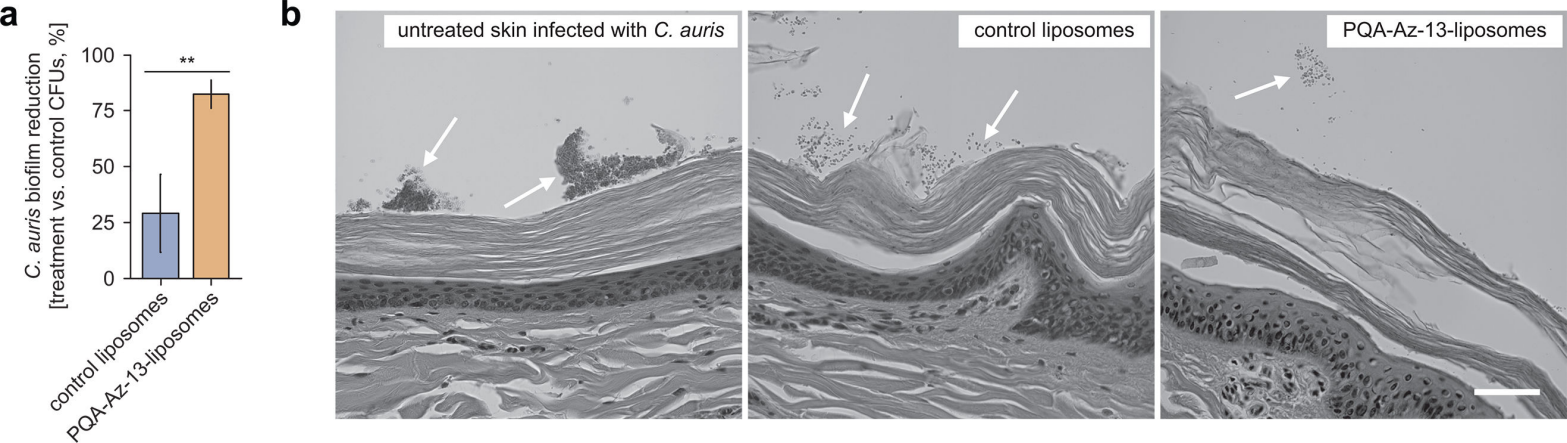
Inhibition of *C. auris* biofilms on pig skin after addition of PQA-Az-13 liposomes (**a**). The histology analysis of pig skin samples (arrows indicate the presence of fungal biofilm) (**b**).

Finally, to evaluate the efficacy of the developed liposomal formulation on skin biofilm colonization by *C. auris*, we utilized an *ex vivo* pig skin model. We decided to use porcine skin due to the similarity it has to human skin in terms of skin thickness, mechanisms of skin repair, and the types and distribution of skin cells (55 – 58). Moreover, the used skin samples were obtained from a human-sized miniature swine breed (Wisconsin Miniature Swine) since miniature varieties of pigs more closely mimic human body composition (55, 59), in contrast to conventional pigs which have been bred for increased muscle mass and size. All experiments were performed with the use of the B11203 strain of *C. auris*, isolated from a patient in India and phylogenetically placed in the South Asian or Indian/Pakistan clade. We inoculated the surface of skin samples with *C. auris* to allow for skin colonization and biofilm growth. We then examined the impact of treatment with PQA-Az-13-liposomes and control liposomes, respectively. As shown in Fig. 7, the PQA-Az-13-loaded nanoformulation caused an 83% reduction of *C. auris* biofilm in the cutaneous environment, confirming their fungicidal activity. Interestingly, the control liposomes also showed some inhibition toward *C. auris* biofilm, albeit to a much lesser extent (30% inhibition). It can, therefore, be concluded that the observed differences in activity are due to the availability of this candicidal agent via its nanosized form. In our study, we used positively charged vesicles and this property may, at least in part, explain the observed activity for the control liposomes. However, it should be noted there have only been a limited number of formulations which have been tested on *C. auris*, and according to our knowledge, this is the first liposomal composition tested on this *ex vivo* model. Therefore, further research is needed to understand the effects of such nanoformulation effects on these fungi.

PQA-Az-13, a novel antifungal, demonstrates inhibition of *C. auris* biofilms both *in vitro* and *ex vivo*, showing promise for effectively treating skin infections in patient-focused scenarios. However, its current physical–chemical properties restrict its potential application as a disinfectant for sterilizing surfaces in *C. auris*-infested settings. Enhancements in its chemical properties, such as improved water solubility, would significantly broaden the antifungal applications of this innovative molecule. The advantages of applying lipid formulations to the skin is worth noting. These benefits encompass enhanced skin penetration, reduced dosages, increased drug concentration, and improved biocompatibility. Nonetheless, there are disadvantages, such as challenges in clinical translation and scalability, as well as occasional issues related to physical and chemical stability.

### Conclusions

*C. auris* has emerged as a major pathogenic fungus that efficiently colonizes the skin. Its multidrug- and pandrug-resistant strains force an urgent search for compounds and strategies that combat this opportunistic pathogen. In this study, a new original compound was synthesized (PQA-Az-13) which showed high activity against clinical isolates of *C. auris* and was further analyzed with a proteomics approach, to determine the mode of action of PQA-Az-13 against *C. auris*. Our study indicates that the antifungal activity of PQA-Az-13 may be attributed to its complete or partial inhibition of numerous enzymatic proteins within *C. auris* biofilms. These proteins are primarily involved in amino acid biosynthesis, metabolic processes, and general energy production, including fatty acid degradation and glycolysis/gluconeogenesis. It is likely that, in response to the inhibitory effects of PQA-Az-13, compensatory mechanisms result in the upregulation of metabolic pathways responsible for aromatic amino acid biosynthesis as well as steroid biosynthesis. Recognizing that its poor solubility will limit options for formulation as a solution, the compound was encapsulated in cationic liposomes. PQA-Az-13-liposomes showed excellent activity in an *ex vivo* porcine skin model of *C. auris* skin colonization in sweat, mimicking conditions on the surface of the skin. The results confirmed that the proposed approach is a promising alternative to existing therapies and antifungals which can be used for the reduction of *Candida auris* colonization on the skin.

## Data Availability

The mass spectrometry proteomics data have been deposited to the ProteomeXchange Consortium via the PRIDE partner repository with the dataset identifier PXD046911 and DOI 10.6019/PXD046911.

## SUPPLEMENTAL MATERIAL

Additional experimental details, Table S1, Table S1, Table S3 aac.00955-23-s0001.pdf Synthesis of PQA-Az-13, general chemistry information, synthetic procedures, physicochemical properties prediction and developability studies, 1H NMR, 13C NMR, 19F NMR; Table S1 (Predicted and calculated molecular properties of PQA-AZ-13); Table S2 (Cellular proteomics in control and PQA-Az-13 treated *C. auris* cells); Table S3 (Fitting parameters for the SAXS patterns of control liposomes and liposomes with PQA-Az-13).

ASM does not own the copyrights to Supplemental Material that may be linked to, or accessed through, an article. The authors have granted ASM a non-exclusive, world-wide license to publish the Supplemental Material files. Please contact the corresponding author directly for reuse.
