# Supplementary material for: Liposomal formulation of a new antifungal hybrid compound provides protection against Candida auris in the ex vivo skin colonization model
Source: Antimicrob Agents Chemother. 2023 Dec 11;68(1):e00955-23. doi: 10.1128/aac.00955-23 (PMC10777852; doi:10.1128/aac.00955-23)
Supplement: Additional experimental details, Table S1, Table S1, Table S3 — Synthesis of PQA-Az-13, general chemistry information, synthetic procedures, physicochemical properties prediction and developability studies, 1H NMR, 13C NMR, 19F NMR; Table S1 (Predicted and calculated molecular properties of PQA-AZ-13); Table S2 (Cellular proteomics in control and PQA-Az-13 treated C. auris cells); Table S3 (Fitting parameters for the SAXS patterns of control liposomes and liposomes with PQA-Az-13). [file aac.00955-23-s0001.pdf]

Supplementary materials

Liposomal formulation of a new antifungal hybrid compound provides protection in the *ex vivo Candida auris* skin colonization model

Anna Jaromin[1*], Robert Zarnowski[2,3], Adam Markowski[1], Agnieszka Zagórska[4], Chad J. Johnson[5], Haniyeh Etezadi[6], Shinji Kihara[6], Pablo Mota-Santiago[7], Jeniel E. Nett[5,8], Ben J. Boyd[6,9], David R. Andes[2,3*]

[1] Department of Lipids and Liposomes, Faculty of Biotechnology, University of Wrocław, Wrocław, Poland; adam.markowski@uwr.edu.pl (A.M.)

[2] Department of Medicine, School of Medicine & Public Health, University of Wisconsin-Madison, Madison, USA; rzarnowski@medicine.wisc.edu; dra@medicine.wisc.edu (D.R.A.)

[3] Department of Medical Microbiology, School of Medicine & Public Health, University of Wisconsin-Madison, Madison, USA

[4] Department of Medicinal Chemistry, Jagiellonian University Medical College, Cracow, Poland; agnieszka.zagorska@uj.edu.pl (A.Z.)

[5] Department of Medicine, University of Wisconsin, Madison, USA; cjohnson@medicine.wisc.edu (C.J.J.)

[6] Department of Pharmacy, University of Copenhagen, Copenhagen, Denmark; haniyeh.etezadi@sund.ku.dk (H.E.); shinji.kihara@sund.ku.dk (S.K.); ben.boyd@sund.ku.dk (B.B.)

[7] MAX IV Laboratory, Lund University, Lund, Sweden; pablo.mota-santiago@maxiv.lu.se (P.M.S.)

[8] Department of Medical Microbiology and Immunology, University of Wisconsin, Madison, WI 53706, USA; jenett@medicine.wisc.edu (J.E.N.)

[9] Drug Delivery, Disposition and Dynamics, Monash Institute of Pharmaceutical Sciences, Monash University (Parkville Campus), Victoria, Australia

* Correspondence: anna.jaromin@uwr.edu.pl (A.J.); dra@medicine.wisc.edu (D.R.A.)

Table of contents:

Synthesis of PQA-Az-13…………………………………………………………...…..4

General Chemistry Information……………………………………….……………4

Synthetic procedures…………………………………………………………….…..5

Physicochemical properties prediction and developability studies…………………..………6

^{1}H NMR……………………………………………………………………………..7

^{13}C NMR……………………………………………………………………………7

^{19}F NMR……………………………………………………………………...…7

Table S1. Predicted and calculated molecular properties of PQA-AZ-13…………..…………8

Table S2. Cellular proteomics in control and PQA-Az-13 treated *C. auris* cells……………...8

Table S3. Fitting parameters for the SAXS patterns of control liposomes and liposomes with PQA-Az-13……………………………………………………………………………...27

References………………………………………………………………………….27

Synthesis of PQA-Az-13

General Chemistry Information. All the reagents were purchased from commercial suppliers: Sigma-Aldrich (Saint Louis, USA), Merck (Darmstadt, Germany), Fluorochem (Hadfield, UK), Acros Organics (Hampton, USA), Apollo Scientific (Bredbury, UK), and were used without further purification. Analytical thin-layer chromatography (TLC) was performed on Merck Kieselgel 60 F_{254} (0.25 mm) precoated aluminum sheets (Merck, Darmstadt, Germany). The compound was visualized with UV light in a 2.9 % solution of ninhydrin in acetone. Column chromatography was performed using silica gel (particle size 0.063-0.200 mm; 70-230 Mesh ATM) purchased from Merck (Merck, Darmstadt, Germany). The microwave reactions were conducted in a Discover LabMate apparatus (CEM Corporation, Matthews, USA). The UPLC-MS or UPLC-MS/MS analyses were done on an UPLC-MS/MS system comprising a Waters ACQUITY UPLC (Waters Corporation, Milford, USA) coupled with a Waters TQD mass spectrometer (electrospray ionization mode ESI with tandem quadrupole). Chromatographic separations were carried out using an ACQUITY UPLC BEH (bridged ethyl hybrid) C18 column: 2.1 × 100 mm with a particle size of 1.7 µm. The column was maintained at 40 °C and eluted under gradient conditions using 95 to 0% of eluent A over 10 min, at a flow rate of 0.3 mL/min. Eluent A: 0.1% solution of formic acid in water (v/v); eluent B: 0.1% solution of formic acid in acetonitrile (v/v). A total of 10 µL of each sample was injected, and chromatograms were recorded using a Waters eλ PDA detector. The spectra were analyzed in the range of 200−700 nm with 1.2 nm resolution and at a sampling rate of 20 points/s. The UPLC/MS purity of all the test compounds and key intermediates was determined to be >95%.

^{1}H NMR and ^{13}C NMR spectra were recorded using an FT-NMR 500 MHz spectrometer (Joel Ltd., Akishima, Tokyo, Japan), and a ^{19}F NMR spectrum was obtained in a Varian Mercury spectrometer (282 MHz, Varian Inc., Palo Alto, USA). Chemical shifts are reported as δ values (ppm) relative to TMS δ = 0 (^{1}H) as the internal standard. The *J* values are expressed in hertz (Hz). Signal multiplicities are represented by the following abbreviations: s (singlet), br s (broad singlet), d (doublet), t (triplet), q (quartet), and m (multiplet). Elemental analyses were conducted using a Vario EL III elemental analyzer (Elementar Analysensysteme GmbH, Langenselbold, Germany). Elemental analyses were found to be within ±0.4% of the theoretical values. Melting point was determined on a Büchi Melting Point B-540 apparatus (Büchi Labortechnik, Essen, Germany) using open glass capillaries and are uncorrected.

Synthetic procedures (Figure 1). A mixture of 3-chloromethyl-pyrrolidine-1-carboxylic acid *tert*-butyl ester, 1-(3-trifluoromethyl)phenyl-piperazine, cesium carbonate, and a catalytic amount of potassium iodide in DMF (5 mL) was stirred at 120 °C for 2 h under microwave irradiation. After that time, the reaction mixture was cooled to room temperature and the mixture was extracted with ethyl acetate (20 mL). The organic layer was washed with water (20 mL) and then dried over anhydrous sodium sulfate. After evaporation of ethyl acetate, the product was purified by column chromatography over silica gel using dichloromethane/acetone (70/30, v/v) as eluent. The crude products were obtained after deprotection of the *tert*-butyloxycarbonyl group, conducted according to a general procedure. A mixture of 7-chloroindazole and 1-(3-trifluoromethylphenyl)-4-(pyrrolidin-3-ylmethyl)piperazine, triethylamine, and 2 mL of toluene were stirred at 100 °C for 4 h under microwave irradiation. After that time, the solvent was evaporated under reduced pressure. Next, the reaction mixture was purified by column chromatography over silica gel using dichloromethane/methanol (9/1, v/v) as eluent.

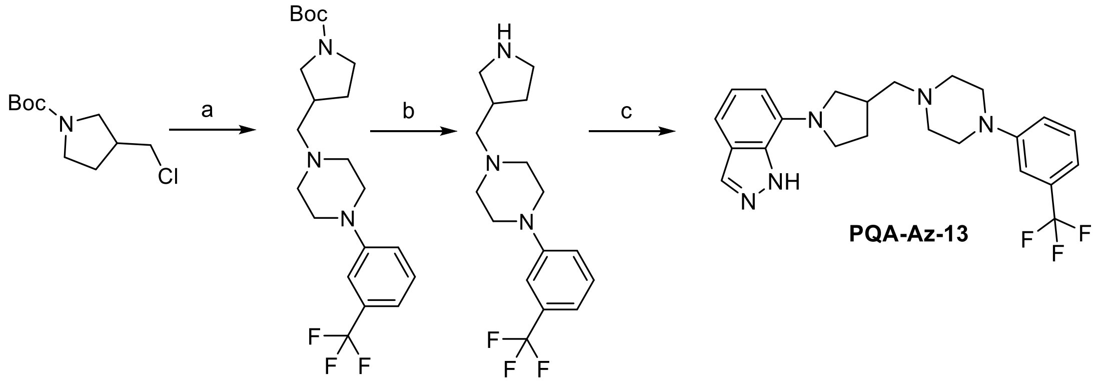

Figure 1. Synthesis of PQA-Az-13.

Reagents and conditions: (a) $CsCO_3$, KI, DMF, M/W, 120°C; (b) TFA/DCM, rt; (c) 7-chloroindazole, Et_3N, toluene, M/W 100°C.

7-(3-((4-(3-Trifluoromethylphenylpiperazin-1-yl)methyl)pyrrolidin-1-yl)-1H-indazole (**PQA-Az-13**). Compound **PQA-Az-13** was prepared starting from 7-chloroindazole (0.36 mmol, 1.0 equiv, 0.06 g) and 1-(3-trifluoromethylphenyl)-4-(pyrrolidin-3-ylmethyl)piperazine (1.08 mmol, 3.0 equiv, 0.399 g). Yield 21%, yellowish oil. ^1H NMR ($CDCl_3$, δ): 8.24 (d, J= 3.72 Hz, 1H), 8.15 (s, 1H), 7.70 (dd, J_1= 8.02 Hz, J_2= 0.86 Hz, 1H), 7.53 (dd, J_1= 7.45 Hz, J_2= 0.57 Hz, 1H), 7.36-7.30 (m, 1H), 7.12-7.02 (m, 4H), 3.24-3.20 (m, 4H), 2.66-2.54.(m, 4H), 2.42-2.34 (m, 2H), 2.11-2.04 (m, 2H), 1.76-1.59 (m, 3H), 1.34-1.22 (m, 2H). ^{13}C NMR

($CDCl_3$, δ): 139.51, 136.00, 129.90, 129.41, 129.23, 124.40, 122.78, 122.37, 120.17, 117.01, 116.91, 110.98, 108.99, 103.11, 59.88, 55.29, 42.27, 32.01, 31.71, 29.79, 29.41, 23.58, 22.78 (multiplets CF_3 not detected). ^{19}F NMR (282 MHz, $CDCl_3$): δ (ppm): -62.99 (s, 3F). Formula: $C_{23}H_{26}F_3N_5$; MS (ESI^+): *m* / *z* 430.2 [$M+H^+$].

Physicochemical properties prediction and developability studies

Instant JChem 20.19.0, 2020, ChemAxon (http://www.chemaxon.com) was used for physicochemical properties prediction [1], whereas The PAINS and Brenk alerts were determined using the SwissADME server [2].

^{1}H NMR

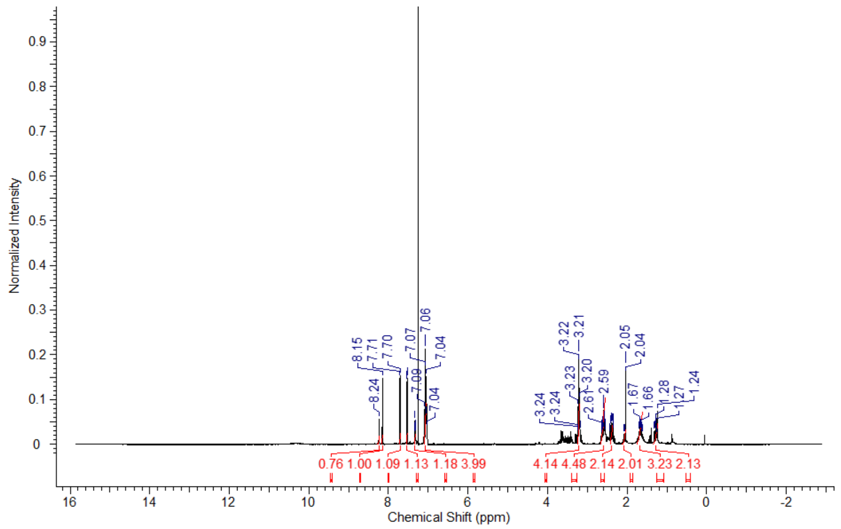

^{13}C NMR

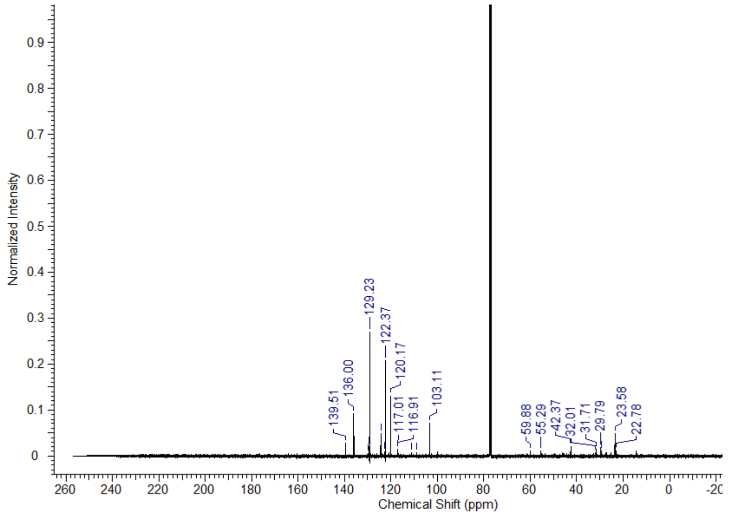

^{19}F NMR

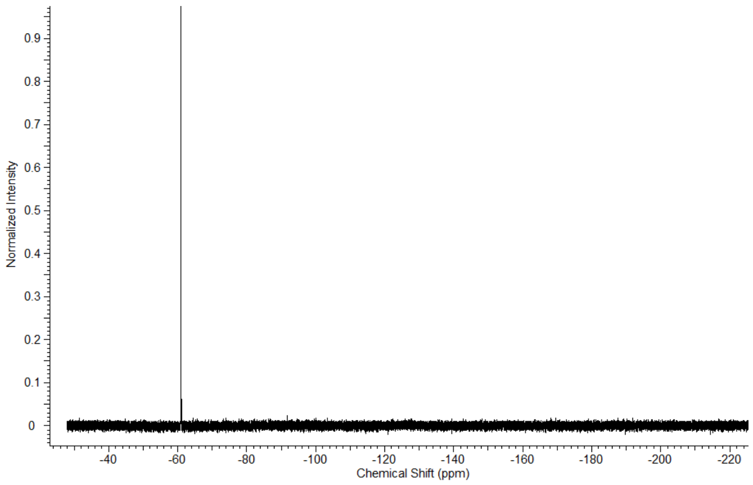

Table S1. Predicted and calculated molecular properties of PQA-AZ-13.

	Lipinski's rule of 5				Veber filter	
	Log $P_{o/w}$	MW	HBD	HBA	RB	TPSA
PQA-AZ-13	4.1	429.5	1	4	5	38.4

Properties calculated using InstantJChem software (ChemAxon): Log $P_{o/w}$ - Predicted octanol/water partition coefficient; MW – molecular weight; HBD – hydrogen bond donor in pH 7.4; HBA – hydrogen bond acceptor in pH 7.4; RB – rotatable bonds; TPSA – total polar surface area.

Table S2. Cellular proteomics in control and PQA-Az-13 treated *C. auris* cells.

Identified Proteins	Accession Number	Gene name (ORF)	Alternate ID	Control	PQA-Az-13	Control/AZ	Z score
Uncharacterized protein	A0A1D8PC56	CAALFM_C100210CA	orf19	0	0.95	drug induced	-2.021
DNA-directed RNA polymerase	A0A1D8PCA4	CAALFM_C100640CA	RPO41	0	1.43	drug induced	-2.021
Nma111p	A0A1D8PCD2	CAALFM_C100990CA	NMA111	0	0	drug induced	-2.021
Trm2p	A0A1D8PCG3	CAALFM_C101380CA	TRM2	0	0.45	drug induced	-2.021
Mrs7p	A0A1D8PCG9	CAALFM_C101320WA	MRS7	0	1.9	drug induced	-2.021
Uncharacterized protein	A0A1D8PCL4	CAALFM_C101910WA	orf19	0	0.97	drug induced	-2.021
Eukaryotic translation initiation factor 2A	A0A1D8PCQ3	CAALFM_C102430CA	orf19	0	4.05	drug induced	-2.021
Threonine--tRNA ligase	A0A1D8PCV0	CAALFM_C102930CA	MST1	0	0	drug induced	-2.021
Aminotran_5 domain-containing protein	A0A1D8PCX5	CAALFM_C102970WA	orf19	0	0	drug induced	-2.021
Proteasome regulatory particle lid subunit	A0A1D8PD15	CAALFM_C103520WA	RPN3	0	1.9	drug induced	-2.021
Ifd6p	A0A1D8PD78	CAALFM_C104140WA	IFD6	0	0.5	drug induced	-2.021
Putative dehydrogenase	A0A1D8PDC5	CAALFM_C104750WA	IFE1	0	0	drug induced	-2.021
Coatomer subunit gamma	A0A1D8PDE3	CAALFM_C104830WA	SEC21	0	2.55	drug induced	-2.021
Chromatin-remodeling ATPase	A0A1D8PE13	CAALFM_C107340WA	orf19	0	0	drug induced	-2.021
Amidophosphoribosyltransferase	A0A1D8PE37	CAALFM_C107710CA	ADE4	0	0.52	drug induced	-2.021
S-adenosylmethionine-homocysteine S-methyltransferase	A0A1D8PEB0	CAALFM_C108410CA	SAM4	0	0.52	drug induced	-2.021
Bifunctional acetylglutamate kinase/N-acetyl-gamma-glutamyl-phosphate reductase	A0A1D8PEI6	CAALFM_C109290CA	ARG5	0	0.9	drug induced	-2.021
Mevalonate kinase	A0A1D8PEL1	CAALFM_C109460WA	ERG12	0	0.5	drug induced	-2.021
DNA-directed RNA polymerase subunit	A0A1D8PEX3	CAALFM_C110670CA	RPA190	0	0	drug induced	-2.021
DAO domain-containing protein	A0A1D8PEZ9	CAALFM_C110840CA	orf19	0	3.89	drug induced	-2.021
Dolichyl-diphosphooligosaccharide--protein glycosyltransferase subunit WBP1	A0A1D8PF22	CAALFM_C111170WA	WBP1	0	0.98	drug induced	-2.021
Erp5p	A0A1D8PF48	CAALFM_C110940CA	ERP5	0	0	drug induced	-2.021
Phospholipase	A0A1D8PF62	CAALFM_C111590WA	PLD1	0	0	drug induced	-2.021
ApbA_C domain-containing protein	A0A1D8PF76	CAALFM_C111770CA	orf19	0	0	drug induced	-2.021
Uncharacterized protein	A0A1D8PFN9	CAALFM_C113290WA	orf19	0	0	drug induced	-2.021
5'-3' exoribonuclease 1	A0A1D8PFP3	CAALFM_C113420CA	KEM1	0	0.97	drug induced	-2.021
High-affinity iron permease	A0A1D8PFV0	CAALFM_C114130WA	FTR1	0	0	drug induced	-2.021
Sphingolipid transporter	A0A1D8PFW9	CAALFM_C114300CA	NCR1	0	1.35	drug induced	-2.021
Uncharacterized protein	A0A1D8PG26	CAALFM_C200360CA	orf19	0	0	drug induced	-2.021
Kynurenine--oxoglutarate transaminase	A0A1D8PGR0	CAALFM_C202950WA	BNA31	0	5.2	drug induced	-2.021
Small nuclear ribonucleoprotein G	A0A1D8PH20	CAALFM_C203880CA	orf19	0	0.52	drug induced	-2.021
Mitochondrial 37S ribosomal protein MRP51	A0A1D8PH91	CAALFM_C204790CA	orf19	0	1.54	drug induced	-2.021
Lem3p	A0A1D8PHB8	CAALFM_C205110WA	LEM3	0	0.45	drug induced	-2.021
Sulfite reductase	A0A1D8PHL8	CAALFM_C206170CA	ECM17	0	2.04	drug induced	-2.021
Gre2p	A0A1D8PHQ6	CAALFM_C206720WA	GRE2	0	2.46	drug induced	-2.021
AB hydrolase-1 domain-containing protein	A0A1D8PHY7	CAALFM_C207650CA	orf19	0	0	drug induced	-2.021
Uncharacterized protein	A0A1D8PHZ2	CAALFM_C207790CA	orf19	0	0	drug induced	-2.021
Rho family GTPase	A0A1D8PHZ5	CAALFM_C207750WA	RHO2	0	0	drug induced	-2.021
Serine/threonine protein kinase	A0A1D8PI07	CAALFM_C207130CA	RCK2	0	0.5	drug induced	-2.021
Rnr22p	A0A1D8PI17	CAALFM_C207570WA	RNR22	0	6.06	drug induced	-2.021
Uncharacterized protein	A0A1D8PI34	CAALFM_C208200WA	orf19	0	1.88	drug induced	-2.021
Structural maintenance of chromosomes protein	A0A1D8PI59	CAALFM_C208560WA	SMC2	0	1	drug induced	-2.021
Bifunctional farnesyl-diphosphate farnesyltransferase/squalene synthase	A0A1D8PI71	CAALFM_C208610WA	ERG9	0	3.05	drug induced	-2.021
ATP-binding cassette transporter	A0A1D8PIM2	CAALFM_C209990CA	YOR1	0	0	drug induced	-2.021
Ato2p	A0A1D8PJ42	CAALFM_C300930WA	ATO2	0	0	drug induced	-2.021
DUF155 domain-containing protein	A0A1D8PJ57	CAALFM_C301190CA	orf19	0	0.45	drug induced	-2.021
Beta-glucosidase	A0A1D8PJB5	CAALFM_C301820WA	orf19	0	0	drug induced	-2.021
Uncharacterized protein	A0A1D8PJE5	CAALFM_C302270WA	orf19	0	1.02	drug induced	-2.021
Guanine nucleotide-binding protein subunit alpha	A0A1D8PJG1	CAALFM_C302240CA	GPA2	0	0.45	drug induced	-2.021
D-aminoacyl-tRNA deacylase	A0A1D8PJK2	CAALFM_C303010CA	DTD2	0	1.9	drug induced	-2.021
Putative metallocarboxypeptidase	A0A1D8PJK3	CAALFM_C303030CA	ECM14	0	0.97	drug induced	-2.021
D-lactate dehydrogenase	A0A1D8PJK5	CAALFM_C303040WA	AIP2	0	3.8	drug induced	-2.021
Phospholipid-transporting ATPase	A0A1D8PJN3	CAALFM_C303250WA	orf19	0	0	drug induced	-2.021
Alanine transaminase	A0A1D8PJP4	CAALFM_C303480CA	ALT1	0	0.52	drug induced	-2.021
Ctp1p	A0A1D8PJV4	CAALFM_C304270CA	CTP1	0	1.05	drug induced	-2.021
Bifunctional glutathione transferase/peroxidase	A0A1D8PJX4	CAALFM_C303600CA	GTT12	0	0	drug induced	-2.021
Dolichyl-diphosphooligosaccharide--protein glycosyltransferase subunit 1	A0A1D8PK87	CAALFM_C305530WA	OST1	0	0.98	drug induced	-2.021
L-serine/L-threonine ammonia-lyase	A0A1D8PKG0	CAALFM_C306280WA	orf19	0	1.05	drug induced	-2.021
Uncharacterized protein	A0A1D8PKG5	CAALFM_C306370CA	orf19	0	0	drug induced	-2.021
Acetyltransferase	A0A1D8PKI3	CAALFM_C306490WA	orf19	0	5.07	drug induced	-2.021
Uncharacterized protein	A0A1D8PKI6	CAALFM_C306050CA	orf19	0	0.97	drug induced	-2.021
RING-type domain-containing protein	A0A1D8PKR8	CAALFM_C307380WA	orf19	0	0	drug induced	-2.021
V-type proton ATPase subunit	A0A1D8PKX3	CAALFM_C400020WA	VMA6	0	1.88	drug induced	-2.021
Cnh1p	A0A1D8PKY1	CAALFM_C400040WA	CNH1	0	0.45	drug induced	-2.021
Histidine biosynthesis trifunctional protein	A0A1D8PKY7	CAALFM_C400140CA	HIS4	0	2.41	drug induced	-2.021
Uncharacterized protein	A0A1D8PKZ5	CAALFM_C307690CA	orf19	0	0	drug induced	-2.021
Histidinol-phosphate transaminase	A0A1D8PL37	CAALFM_C400650WA	HIS5	0	1.05	drug induced	-2.021
Hydroxyacyl-thioester dehydratase	A0A1D8PLR8	CAALFM_C403200CA	orf19	0	0	drug induced	-2.021
Mitochondrial 54S ribosomal protein YmL22	A0A1D8PLT6	CAALFM_C403410WA	orf19	0	0	drug induced	-2.021
Dao2p	A0A1D8PLT7	CAALFM_C403380CA	DAO2	0	0	drug induced	-2.021
Ubiquitin-specific protease	A0A1D8PLU4	CAALFM_C403440CA	DOT4	0	0	drug induced	-2.021
Uncharacterized protein	A0A1D8PLX7	CAALFM_C403880WA	orf19	0	0	drug induced	-2.021
Uncharacterized protein	A0A1D8PLZ2	CAALFM_C403960WA	orf19	0	1.05	drug induced	-2.021
Endo/exonuclease/phosphatase domain-containing protein	A0A1D8PM02	CAALFM_C404140WA	orf19	0	1.57	drug induced	-2.021
Ald6p	A0A1D8PM94	CAALFM_C405130CA	ALD6	0	0.9	drug induced	-2.021
Aspartokinase	A0A1D8PMB8	CAALFM_C405540WA	HOM3	0	2.41	drug induced	-2.021
Aromatic-amino-acid:2-oxoglutarate transaminase	A0A1D8PMC5	CAALFM_C405560CA	ARO9	0	1.9	drug induced	-2.021
I-AAA protease	A0A1D8PME0	CAALFM_C405670WA	YME1	0	1.42	drug induced	-2.021
Cwh41p	A0A1D8PMH9	CAALFM_C406100WA	CWH41	0	0	drug induced	-2.021
Uncharacterized protein	A0A1D8PMM8	CAALFM_C406710WA	orf19	0	1.02	drug induced	-2.021
Oye32p	A0A1D8PMP0	CAALFM_C406780CA	OYE32	0	1.04	drug induced	-2.021
Glycerol-3-phosphate dehydrogenase	A0A1D8PMP1	CAALFM_C406760WA	GUT2	0	3.3	drug induced	-2.021
Anthranilate phosphoribosyltransferase	A0A1D8PMR2	CAALFM_C407090CA	TRP4	0	1.43	drug induced	-2.021
Mitochondrial 54S ribosomal protein MRPL1	A0A1D8PMT8	CAALFM_C500030WA	orf19	0	2.85	drug induced	-2.021
Fcr3p	A0A1D8PN91	CAALFM_C501810WA	FCR3	0	0	drug induced	-2.021
Uncharacterized protein	A0A1D8PNH7	CAALFM_C502690WA	orf19	0	0	drug induced	-2.021
Dap2p	A0A1D8PNK0	CAALFM_C502970WA	DAP2	0	0	drug induced	-2.021
Aldolase_II domain-containing protein	A0A1D8PNQ3	CAALFM_C503670CA	orf19	0	0	drug induced	-2.021
Mtr10p	A0A1D8PNS3	CAALFM_C503760CA	MTR10	0	0	drug induced	-2.021
Protein disulfide isomerase	A0A1D8PNX4	CAALFM_C504340WA	orf19	0	0	drug induced	-2.021

Methionine--tRNA ligase	A0A1D8PP21	CAALFM_C504700CA	MES1	0	2.01	drug induced	-2.021
2-aminoadipate transaminase	A0A1D8PPA8	CAALFM_C600210WA	orf19	0	0	drug induced	-2.021
Ardp	A0A1D8PPB1	CAALFM_C600150WA	ARD	0	1.02	drug induced	-2.021
Uncharacterized protein	A0A1D8PPB2	CAALFM_C600200CA	orf19	0	0.5	drug induced	-2.021
Ebp1p	A0A1D8PPK1	CAALFM_C601180CA	EBP1	0	5.11	drug induced	-2.021
E3 ubiquitin-protein ligase	A0A1D8PPK7	CAALFM_C601350WA	orf19	0	0	drug induced	-2.021
Acf2p	A0A1D8PPP4	CAALFM_C601690WA	ACF2	0	0	drug induced	-2.021
PHD domain-containing protein	A0A1D8PQ51	CAALFM_C603290WA	orf19	0	0	drug induced	-2.021
1,4-alpha-glucan branching enzyme	A0A1D8PQ59	CAALFM_C603340CA	GLC3	0	1.94	drug induced	-2.021
ATP-binding cassette transporter	A0A1D8PQ95	CAALFM_C603840CA	SNQ2	0	6.15	drug induced	-2.021
3-isopropylmalate dehydrogenase	A0A1D8PQK5	CAALFM_C700400WA	LEU2	0	6.65	drug induced	-2.021
Fgr2p	A0A1D8PQL0	CAALFM_C700480WA	FGR2	0	0	drug induced	-2.021
Putative ferric-chelate reductase	A0A1D8PQM2	CAALFM_C700430WA	orf19	0	1.85	drug induced	-2.021
DNA-directed RNA polymerase subunit beta	A0A1D8PQM4	CAALFM_C700570WA	RPA135	0	0	drug induced	-2.021
Uncharacterized protein	A0A1D8PQP9	CAALFM_C700920CA	orf19	0	0	drug induced	-2.021
Sla2p	A0A1D8PRF5	CAALFM_C703810WA	SLA2	0	0	drug induced	-2.021
Nicotinate phosphoribosyltransferase	A0A1D8PRI0	CAALFM_C704040CA	NPT1	0	0.45	drug induced	-2.021
TauD domain-containing protein	A0A1D8PRK5	CAALFM_C704310CA	orf19	0	0.5	drug induced	-2.021
Uncharacterized protein	A0A1D8PRK7	CAALFM_C704280CA	orf19	0	0.5	drug induced	-2.021
NAD(P)-bd_dom domain-containing protein	A0A1D8PRL3	CAALFM_CR00090CA	CAALFM_CR00090CA	0	0.5	drug induced	-2.021
Aminotran_1_2 domain-containing protein	A0A1D8PRL8	CAALFM_CR00130CA	CAALFM_CR00130CA	0	0	drug induced	-2.021
Mitochondrial 54S ribosomal protein YmL9	A0A1D8PRP6	CAALFM_CR00490WA	CAALFM_CR00490WA	0	1.02	drug induced	-2.021
Uncharacterized protein	A0A1D8PRQ2	CAALFM_CR00310CA	CAALFM_CR00310CA	0	0	drug induced	-2.021
Trans-hexaprenyltranstransferase	A0A1D8PRR4	CAALFM_CR00570WA	CAALFM_CR00570WA	0	0.53	drug induced	-2.021
Argininosuccinate synthase	A0A1D8PRR5	CAALFM_CR00620CA	ARG1	0	4.59	drug induced	-2.021
Mitochondrial 37S ribosomal protein MRPS28	A0A1D8PRY0	CAALFM_CR01370CA	CAALFM_CR01370CA	0	2.85	drug induced	-2.021
Putative proline--tRNA ligase	A0A1D8PRZ3	CAALFM_CR01480WA	PRS	0	1.02	drug induced	-2.021
Anthranilate synthase	A0A1D8PS03	CAALFM_CR01590CA	TRP2	0	0.97	drug induced	-2.021
Centrin	A0A1D8PSM8	CAALFM_CR04020CA	CAALFM_CR04020CA	0	0	drug induced	-2.021
Threonine aldolase	A0A1D8PSW2	CAALFM_CR04880WA	CAALFM_CR04880WA	0	1.49	drug induced	-2.021
Mitochondrial 2-oxodicarboxylate carrier	A0A1D8PT04	CAALFM_CR05480WA	CAALFM_CR05480WA	0	1.55	drug induced	-2.021
Aminotran_1_2 domain-containing protein	A0A1D8PTM0	CAALFM_CR08000CA	CAALFM_CR08000CA	0	0.45	drug induced	-2.021
Uncharacterized protein	A0A1D8PTU4	CAALFM_CR08920WA	CAALFM_CR08920WA	0	0	drug induced	-2.021
U6 snRNA-associated Sm-like protein LSm1	A0A1D8PTV1	CAALFM_CR08930CA	LSM1	0	2.4	drug induced	-2.021
Putative hydrolase	A0A1D8PTV6	CAALFM_CR08840CA	NIT2	0	0.45	drug induced	-2.021
Actin-related protein 2	A0A1D8PTX1	CAALFM_CR08950WA	ARP2	0	1.57	drug induced	-2.021
Uncharacterized protein	A0A1D8PTZ1	CAALFM_CR09330CA	CAALFM_CR09330CA	0	1.45	drug induced	-2.021
DNA-directed RNA polymerase subunit	A0A1D8PUA6	CAALFM_CR10680WA	RPO21	0	0	drug induced	-2.021
C-22 sterol desaturase	G1UB11	CAALFM_C702840CA	ERG5	0	1.52	drug induced	-2.021
Sterol 24-C-methyltransferase	O74198	CAALFM_C302150CA	ERG6	0	2.55	drug induced	-2.021
Ribosome biogenesis protein C3_06160C_A	P0CU36	CAALFM_C306160CA	CAALFM_C306160CA	0	0.52	drug induced	-2.021
ADP-ribosylation factor	P22274	CAALFM_CR08700CA	ARF1	0	0	drug induced	-2.021
Glucan 1,3-beta-glucosidase	P29717	CAALFM_C102990CA	XOG1	0	3.8	drug induced	-2.021
Cell growth-regulated gene 1 protein	P56553	CAALFM_C402760CA	CGR1	0	0	drug induced	-2.021
Cysteine desulfurase, mitochondrial	P87185	CAALFM_C700390WA	NFS1	0	2.85	drug induced	-2.021
Amine oxidase	Q59KV5	CAALFM_C206700WA	AMO2	0	0	drug induced	-2.021
Serine/threonine-protein phosphatase SIT4	Q59KY8	CAALFM_C104380WA	SIT4	0	1.54	drug induced	-2.021
Agglutinin-like protein 3	Q59L12	CAALFM_CR07070CA	ALS3	0	0	drug induced	-2.021
Cyclin-dependent kinases regulatory subunit	Q59LQ4	CAALFM_C504070CA	CKS1	0	0.52	drug induced	-2.021
Bifunctional 4-alpha-glucanotransferase/amylo-alpha-1,6-glucosidase	Q59MN2	CAALFM_C405140CA	GDB1	0	1.02	drug induced	-2.021
N-acetyltransferase domain-containing protein	Q59MT3	CAALFM_CR06770CA	CAALFM_CR06770CA	0	0	drug induced	-2.021
Phosphoribosylformylglycinamidine synthase	Q59MZ5	CAALFM_CR04740CA	ADE6	0	0.5	drug induced	-2.021
Gly1p	Q59NC4	CAALFM_C110450WA	GLY1	0	3.3	drug induced	-2.021
NADH-cytochrome b5 reductase 1	Q59P03	CAALFM_C405450CA	CBR1	0	1.9	drug induced	-2.021
Glyoxylate reductase	Q59P08	CAALFM_C405390WA	orf19	0	0	drug induced	-2.021
Threonine dehydratase	Q59P56	CAALFM_C206190WA	ILV1	0	0.9	drug induced	-2.021
Uncharacterized protein	Q59PS7	CAALFM_CR05750WA	CAALFM_CR05750WA	0	0	drug induced	-2.021
Chaperonin-containing T-complex alpha subunit	Q59QB7	CAALFM_C108560WA	TCP1	0	2.41	drug induced	-2.021
Kynureninase	Q59QC4	CAALFM_C108490WA	BNA5	0	0	drug induced	-2.021
Bub3p	Q59QR8	CAALFM_C503240WA	BUB3	0	3.23	drug induced	-2.021
Uncharacterized protein	Q59R27	CAALFM_C703610CA	orf19	0	0	drug induced	-2.021
Putative serine--tRNA ligase	Q59RK3	CAALFM_C600390WA	orf19	0	0	drug induced	-2.021
Mitochondrial intermediate peptidase	Q59RK9	CAALFM_C600340CA	10/1/2021	0	0	drug induced	-2.021
Mitochondrial import inner membrane translocase subunit TIM14	Q59SI2	CAALFM_C400520WA	PAM18	0	0.45	drug induced	-2.021
S-methyl-5'-thioadenosine phosphorylase	Q59ST1	CAALFM_C303790WA	MEU1	0	6.29	drug induced	-2.021
Succinate-semialdehyde dehydrogenase	Q59T88	CAALFM_C101810CA	UGA2	0	1.02	drug induced	-2.021
NAD(P)H-hydrate epimerase	Q59VX9	CAALFM_C102220CA	CAALFM_C102220CA	0	2.01	drug induced	-2.021
4-nitrophenylphosphatase	Q59WC5	CAALFM_C107230WA	PHO15	0	0.5	drug induced	-2.021
Glutathione peroxidase	Q59WW8	CAALFM_C600840WA	GPX2	0	0	drug induced	-2.021
Exopolyphosphatase	Q59X24	CAALFM_C206110WA	orf19	0	0	drug induced	-2.021
WD_REPEATS_REGION domain-containing protein	Q59X91	CAALFM_C500840WA	orf19	0	0	drug induced	-2.021
Cu/Pi carrier	Q59YD1	CAALFM_C209590CA	orf19	0	3.37	drug induced	-2.021
Increased recombination centers protein 22-1	Q59YF4	CAALFM_C209780CA	IRC22-1	0	1.4	drug induced	-2.021
T-complex protein 1 subunit eta	Q59YH4	CAALFM_C501690CA	CCT7	0	2.41	drug induced	-2.021
Actin-related protein 3	Q59Z11	CAALFM_C207320WA	ARP3	0	2.04	drug induced	-2.021
Chaperonin-containing T-complex subunit	Q59Z12	CAALFM_C207310WA	CCT5	0	1.02	drug induced	-2.021
Smr domain-containing protein	Q59Z54	CAALFM_C206920CA	orf19	0	0	drug induced	-2.021
AAA family ATPase peroxin 6	Q59ZE6	CAALFM_C205440WA	PEX6	0	0	drug induced	-2.021
Coatomer subunit zeta	Q5A006	CAALFM_CR03600CA	CAALFM_CR03600CA	0	0.5	drug induced	-2.021
Sterol-4-alpha-carboxylate 3-dehydrogenase	Q5A1B0	CAALFM_C406270CA	ERG26	0	3.37	drug induced	-2.021
Probable kinetochore protein NDC80	Q5A216	CAALFM_CR02680WA	NDC80	0	0.52	drug induced	-2.021
Ribose phosphate diphosphokinase subunit	Q5A2L2	CAALFM_C207890WA	PRS5	0	0.5	drug induced	-2.021
Blm3p	Q5A2T3	CAALFM_C208010WA	BLM3	0	0	drug induced	-2.021
Mitochondrial presequence protease	Q5A301	CAALFM_C306230WA	CYM1	0	0	drug induced	-2.021
Dodecenoyl-CoA isomerase	Q5A318	CAALFM_CR08690CA	ECI1	0	0.45	drug induced	-2.021
Glutathione synthetase	Q5A359	CAALFM_CR08370WA	GSH2	0	15.21	drug induced	-2.021
Type 2C protein phosphatase	Q5A388	CAALFM_CR08160WA	PTC5	0	1.52	drug induced	-2.021
Protein transport protein SEC23	Q5A455	CAALFM_C405690WA	SEC23	0	1.05	drug induced	-2.021
DNA-directed RNA polymerase II core subunit	Q5A462	CAALFM_C405630WA	orf19	0	0	drug induced	-2.021
Frp6p	Q5A4K0	CAALFM_C601430CA	FRP6	0	0	drug induced	-2.021
Glucan 1\3-alpha-glucosidase ROT2	Q5A4X3	CAALFM_C500220WA	ROT2	0	0.98	drug induced	-2.021
Ribose phosphate diphosphokinase subunit	Q5A4X7	CAALFM_C500260WA	orf19	0	0.5	drug induced	-2.021
Endonuclease	Q5A4X9	CAALFM_C500280CA	orf19	0	0	drug induced	-2.021
Metallophos domain-containing protein	Q5A5Q7	CAALFM_C210630WA	orf19	0	0.5	drug induced	-2.021
Uncharacterized protein	Q5A5V8	CAALFM_C404160WA	orf19	0	0.5	drug induced	-2.021

Serine/threonine-protein kinase ATG1	Q5A649	CAALFM_C404450CA	ATG1	0	0	drug induced	-2.021
Acyl-CoA desaturase	Q5A747	CAALFM_C108360CA	OLE1	0	1.43	drug induced	-2.021
Multiple drug resistance-associated protein-like transporter 1	Q5A762	CAALFM_C108210CA	MLT1	0	0.53	drug induced	-2.021
Pre-mRNA-splicing factor PRP46	Q5A7Q3	CAALFM_C300500CA	PRP46	0	1.42	drug induced	-2.021
rRNA-processing protein	Q5A7Q9	CAALFM_C300560CA	SOF1	0	0	drug induced	-2.021
Uncharacterized protein	Q5A7S3	CAALFM_C300640WA	orf19	0	0	drug induced	-2.021
Carbamoyl-phosphate synthase	Q5A8A6	CAALFM_CR01330WA	CPA2	0	2.01	drug induced	-2.021
SKI complex subunit WD repeat protein	Q5A8I4	CAALFM_C603890CA	SKI8	0	0	drug induced	-2.021
Uncharacterized protein	Q5A8I5	CAALFM_C603880WA	orf19	0	1.02	drug induced	-2.021
MoCF_biosynth domain-containing protein	Q5A8J1	CAALFM_C603820CA	orf19	0	0.45	drug induced	-2.021
Agglutinin-like protein 5	Q5A8T7	CAALFM_C603690WA	ALS5	0	0	drug induced	-2.021
WD_REPEATS_REGION domain-containing protein	Q5A9D8	CAALFM_CR01410CA	CAALFM_CR01410CA	0	1.42	drug induced	-2.021
Arp2/3 complex 34 kDa subunit	Q5AA47	CAALFM_C106080CA	ARC35	0	0.45	drug induced	-2.021
Mitochondrial 54S ribosomal protein YmL19	Q5AAS9	CAALFM_C106790CA	MRPL19	0	0.45	drug induced	-2.021
Vacuolar protein sorting-associated protein 29	Q5AB94	CAALFM_C100320WA	orf19	0	1.02	drug induced	-2.021
Uncharacterized protein	Q5ABA5	CAALFM_C100420WA	orf19	0	2.05	drug induced	-2.021
Ornithine carbamoyltransferase	Q5ABU0	CAALFM_C603230WA	ARG3	0	5.05	drug induced	-2.021
Uricase	Q5ACV3	CAALFM_C200180CA	orf19	0	0	drug induced	-2.021
Cystathionine beta-lyase	Q5ACX5	CAALFM_C200390CA	orf19	0	2.85	drug induced	-2.021
Allantoicase	Q5AD02	CAALFM_C200630CA	orf19	0	0	drug induced	-2.021
M-AAA protease subunit	Q5AD10	CAALFM_C200700WA	orf19	0	1.42	drug induced	-2.021
GFO_IDH_MocA domain-containing protein	Q5AD31	CAALFM_C200880WA	orf19	0	0.5	drug induced	-2.021
Methylthioribose-1-phosphate isomerase	Q5AD59	CAALFM_C201160WA	MRI1	0	0.45	drug induced	-2.021
Aldo-keto reductase superfamily protein	Q5ADM5	CAALFM_C306860CA	orf19	0	3.37	drug induced	-2.021
Dolichyl-phosphate-mannose--protein mannosyltransferase 2	Q5ADM9	CAALFM_C306890WA	PMT2	0	1.9	drug induced	-2.021
Putative phosphomutase	Q5ADN2	CAALFM_C306920WA	orf19	0	0.5	drug induced	-2.021
ADP-ribose diphosphatase	Q5ADP5	CAALFM_C307040CA	YSA1	0	2.53	drug induced	-2.021
Glutamate-5-semialdehyde dehydrogenase	Q5ADR2	CAALFM_C307220CA	PRO2	0	1.02	drug induced	-2.021
Ubiquitin	Q5ADS0	CAALFM_C307270CA	UBI4	0	0	drug induced	-2.021
Aldo_ket_red domain-containing protein	Q5ADT3	CAALFM_C307330WA	orf19	0	1.52	drug induced	-2.021
Phosphoribomutase	Q5ADV2	CAALFM_C307490WA	orf19	0	2.53	drug induced	-2.021
Uncharacterized protein	Q5ADW5	CAALFM_C307630CA	orf19	0	0	drug induced	-2.021
Succinate-semialdehyde dehydrogenase	Q5AEC3	CAALFM_C303470WA	orf19	0	1	drug induced	-2.021
Uncharacterized protein	Q5AEE8	CAALFM_C303210WA	orf19	0	0	drug induced	-2.021
Protein transport protein SEC13	Q5AEF2	CAALFM_C303170WA	SEC13	0	2.53	drug induced	-2.021
Probable cytosolic iron-sulfur protein assembly protein 1	Q5AG86	CAALFM_C502730CA	CIA1	0	8.05	drug induced	-2.021
Membrane insertase	Q5AGX4	CAALFM_C701600WA	orf19	0	1.05	drug induced	-2.021
RuvB-like helicase 2	Q5AGZ9	CAALFM_C701810WA	RVB2	0	1.52	drug induced	-2.021
tRNA (guanine-N(7)-)-methyltransferase non-catalytic subunit	Q5AH60	CAALFM_C702340CA	TRM82	0	0	drug induced	-2.021
AMP deaminase	Q5AHA6	CAALFM_C203360WA	orf19	0	0	drug induced	-2.021
Ubiquinone biosynthesis monooxygenase COQ6, mitochondrial	Q5AHZ4	CAALFM_C103550CA	COQ6	0	1.94	drug induced	-2.021
Trehalose-phosphatase	Q5AI14	CAALFM_C103380WA	TPS2	0	2.4	drug induced	-2.021
Autophagy-related protein 21	Q5AI22	CAALFM_C103330CA	ATG21	0	0	drug induced	-2.021
Carboxymethylenebutenolidase	Q5AI87	CAALFM_C102780WA	orf19	0	1.52	drug induced	-2.021
Carbonic anhydrase	Q5AJ71	CAALFM_C301300CA	NCE103	0	0.52	drug induced	-2.021
AAA family ATPase	Q5AJC2	CAALFM_C301760WA	AFG3	0	0	drug induced	-2.021
Histidine--tRNA ligase	Q5AJX0	CAALFM_C505490CA	HTS1	0	3.05	drug induced	-2.021
Proteasome subunit beta	Q5AJZ5	CAALFM_C505310WA	PRE1	0	1.52	drug induced	-2.021
Uncharacterized protein	Q5AK01	CAALFM_C505260WA	PSY4	0	0	drug induced	-2.021
Transcription factor SFL2	Q5AK51	CAALFM_C504830WA	SFL2	0	0	drug induced	-2.021
CTP synthase	Q5AK79	CAALFM_C504570CA	URA7	0	1.02	drug induced	-2.021
Mitochondrial 37S ribosomal protein RSM26	Q5AK82	CAALFM_C504530WA	orf19	0	0.98	drug induced	-2.021
Phosphoacetylglucosamine mutase	Q5AKW4	CAALFM_C113760WA	AGM1	0	0.45	drug induced	-2.021
Amino acid transporter	Q5AL11	CAALFM_C113400CA	AGC1	0	2.4	drug induced	-2.021
Fgr22p	Q5ALJ2	CAALFM_C202600CA	FGR22	0	0	drug induced	-2.021
Ribose phosphate diphosphokinase subunit	Q5ALK3	CAALFM_C202510WA	PRS1	0	0.97	drug induced	-2.021
Glutamine-dependent NAD	Q5ALW6	CAALFM_C201530CA	orf19	0	6.87	drug induced	-2.021
Carbamoyl-phosphate synthase arginine-specific small chain	Q5AML6	CAALFM_C401550CA	CPA1	0	0.5	drug induced	-2.021
3-oxoacyl-[acyl-carrier-protein] synthase	Q5ANC6	CAALFM_C305070WA	CEM1	0	2.4	drug induced	-2.021
Prohibitin	Q5AND0	CAALFM_C305030WA	PHB2	0	3.14	drug induced	-2.021
Afp99p	Q5ANN8	CAALFM_C304200WA	AFP99	0	4.57	drug induced	-2.021
Golgi apparatus membrane protein TVP18	Q5APC0	CAALFM_C109800CA	TVP18	0	1.04	drug induced	-2.021
Ribosome biogenesis protein YTM1	Q5APF0	CAALFM_C109510WA	YTM1	0	0	drug induced	-2.021
GMP synthase [glutamine-hydrolyzing]	Q5APF2	CAALFM_C109490CA	GUA1	0	5.1	drug induced	-2.021
Squalene monooxygenase	Q92206	CAALFM_C108590CA	ERG1	0	1.05	drug induced	-2.021
Phosphoribosylaminoimidazole carboxylase	Q92210	CAALFM_C304520CA	ADE2	0	3.35	drug induced	-2.021
Sulfate adenylyltransferase	Q9Y872	CAALFM_C113870WA	MET3	0	1.9	drug induced	-2.021
Cystathionine gamma-lyase	Q5A362	CAALFM_CR08340WA	CYS3	0.5	27.03	-5.757	-1.070
WD_REPEATS_REGION domain-containing protein	A0A1D8PSI3	CAALFM_CR03520CA	CAALFM_CR03520CA	0.5	23.31	-5.543	-1.022
Glutamate dehydrogenase	A0A1D8PMH8	CAALFM_C406120WA	GDH3	1.05	28.94	-4.785	-0.852
Acyl-coenzyme A oxidase	A0A1D8PQ67	CAALFM_C603560WA	POX1	1.04	26.34	-4.663	-0.825
Isopentenyl-diphosphate delta-isomerase	A0A1D8PLI2	CAALFM_C402280WA	IDI1	0.5	8.15	-4.027	-0.682
Purine nucleoside phosphorylase	A0A1D8PJL8	CAALFM_C303180CA	PNP1	0.52	8.15	-3.970	-0.669
Phenylpyruvate decarboxylase	Q59MU3	CAALFM_CR06860CA	ARO10	1.03	12.72	-3.626	-0.592
Putative Xaa-Pro dipeptidase	A0A1D8PFY5	CAALFM_C114450CA	orf19	0.5	6.11	-3.611	-0.589
3-hydroxyanthranilate 3,4-dioxygenase	Q59K86	CAALFM_CR05440WA	BNA1	0.52	5.83	-3.487	-0.561
Dihydroxy-acid dehydratase	Q5AJY2	CAALFM_C505410CA	ILV3	2.63	28.16	-3.421	-0.546
Uracil phosphoribosyltransferase	Q59QT3	CAALFM_C503390CA	FUR1	0.53	5.57	-3.394	-0.540
Acyl-coenzyme A oxidase	Q5AJD9	CAALFM_C301960CA	POX1-3	16.35	167.81	-3.360	-0.533
Pyruvate carboxylase	A0A1D8PLY4	CAALFM_C403940CA	PYC2	1.03	9.72	-3.238	-0.505
Proteasome endopeptidase complex	A0A1D8PTY6	CAALFM_CR09380WA	PRE8	1.55	14.16	-3.192	-0.495
Proteasome core particle subunit beta 5	Q59Z65	CAALFM_C206820CA	PRE2	1.02	9.14	-3.164	-0.489
Fructose 1,6-bisphosphate 1-phosphatase	A0A1D8PKW2	CAALFM_C307830WA	FBP1	2.61	21.53	-3.044	-0.462
Chorismate synthase	A0A1D8PTK1	CAALFM_CR07710WA	ARO2	2.05	16.79	-3.034	-0.460
Proline dehydrogenase	A0A1D8PNH4	CAALFM_C502600WA	PUT1	0.53	4.29	-3.017	-0.456
Bifunctional UDP-glucose 4-epimerase/aldose 1-epimerase	A0A1D8PCN0	CAALFM_C102150WA	GAL10	1.08	8.7	-3.010	-0.454
Putative cystathionine beta-lyase	A0A1D8PTV7	CAALFM_CR09010CA	CAALFM_CR09010CA	1.02	8.12	-2.993	-0.450
Uncharacterized protein	A0A1D8PLE6	CAALFM_C401460CA	orf19	0.52	4.05	-2.961	-0.443
Aconitate hydratase, mitochondrial	A0A1D8PT27	CAALFM_CR05790CA	ACO2	0.5	3.84	-2.941	-0.439
Lanosterol 14-alpha demethylase	P10613	CAALFM_C500660CA	ERG11	0.5	3.8	-2.926	-0.435
Tryptophan--tRNA ligase	Q5A3P4	CAALFM_C112380CA	WRS1	0.52	3.8	-2.869	-0.423
Homoaconitase, mitochondrial	Q5A644	CAALFM_C404410CA	LYS4	0.55	3.75	-2.769	-0.400
Pyruvate kinase	P46614	CAALFM_C205460WA	CDC19	103.35	700.63	-2.761	-0.398
Multidrug resistance protein CDR2	P78595	CAALFM_C304890WA	CDR2	3.07	20.8	-2.760	-0.398
Proteasome endopeptidase complex	A0A1D8PJ20	CAALFM_C300770CA	SCL1	1.52	10.16	-2.741	-0.394

Aldehyde dehydrogenase	A0A1D8PGT5	CAALFM_C202970CA	ALD5	47.69	310.3	-2.702	-0.385
ATP-dependent (S)-NAD(P)H-hydrate dehydratase	Q59M69	CAALFM_C602030CA	CAALFM_C602030CA	1.05	6.68	-2.670	-0.378
Pleiotropic ABC efflux transporter of multiple drugs CDR1	Q5ANA3	CAALFM_C305220WA	CDR1	4.59	27.6	-2.588	-0.360
GTP-binding nuclear protein	Q59P43	CAALFM_C206310CA	GSP1	1	5.77	-2.529	-0.346
Acetyl-CoA hydrolase	P83773	CAALFM_C502000CA	ACH1	21.07	119.92	-2.509	-0.342
Orotidine 5'-phosphate decarboxylase	P13649	CAALFM_C301350CA	URA3	5.51	31.28	-2.505	-0.341
Glycine cleavage system P protein	A0A1D8PE97	CAALFM_C108400CA	GCV2	2.57	14.19	-2.465	-0.332
Branched-chain-amino-acid aminotransferase	A0A1D8PH42	CAALFM_C204230WA	BAT21	2.05	11.2	-2.450	-0.329
Coatomer subunit alpha	A0A1D8PJB0	CAALFM_C301720CA	orf19	1.03	5.62	-2.448	-0.328
Galactokinase	Q59VY8	CAALFM_C102130CA	GAL1	2.57	13.67	-2.411	-0.320
Aspartyl aminopeptidase	Q59WG6	CAALFM_C110820CA	orf19	2.05	10.76	-2.392	-0.316
Coatomer subunit beta'	A0A1D8PPV5	CAALFM_C602260CA	SEC27	0.52	2.62	-2.333	-0.302
Fesur1p	Q5ADP7	CAALFM_C307060WA	FESUR1	1.55	7.78	-2.328	-0.301
Fe-S cluster-binding ribosome biosynthesis protein	Q5AI20	CAALFM_C103350CA	RLI1	2.06	10.22	-2.311	-0.297
Uncharacterized protein	Q5A915	CAALFM_C101300WA	orf19	0.5	2.48	-2.310	-0.297
1,3-beta-glucan synthase	A0A1D8PCT0	CAALFM_C102420CA	GSC1	1.08	5.35	-2.309	-0.297
V-type proton ATPase subunit C	Q5A2U9	CAALFM_C208190WA	VMA5	0.5	2.41	-2.269	-0.288
NADPH:quinone reductase	Q59Z38	CAALFM_C207070WA	orf19	0.5	2.4	-2.263	-0.287
Serine/threonine-protein phosphatase	A0A1D8PJ80	CAALFM_C301600WA	PPH21	2.05	9.65	-2.235	-0.280
RuvB-like helicase 1	Q5A0W7	CAALFM_C406800WA	RVB1	0.5	2.35	-2.233	-0.280
T-complex protein 1 subunit delta	A0A1D8PLN3	CAALFM_C402780WA	orf19	1.02	4.79	-2.232	-0.280
6,7-dimethyl-8-ribityllumazine synthase	A0A1D8PDK6	CAALFM_C105560WA	RIB4	0.52	2.41	-2.212	-0.275
Uncharacterized protein	A0A1D8PR55	CAALFM_C702610CA	CAALFM_C702610CA	0.52	2.4	-2.207	-0.274
Fre7p	A0A1D8PTG2	CAALFM_CR07290WA	FRE7	0.52	2.4	-2.207	-0.274
Glycogen [starch] synthase	Q5A850	CAALFM_CR00780CA	GSY1	8.69	39.6	-2.188	-0.270
Mis12p	A0A1D8PRL4	CAALFM_CR00080WA	MIS12	0.53	2.4	-2.179	-0.268
Chaperonin-containing T-complex subunit	A0A1D8PMN9	CAALFM_C406830CA	CCT6	1.07	4.82	-2.171	-0.266
Sorbose reductase SOU1	P87219	CAALFM_C406390CA	SOU1	1.05	4.71	-2.165	-0.265
Phosphotransferase	Q59TZ8	CAALFM_CR07150WA	GLK1	12.46	55.69	-2.160	-0.264
Carnitine O-acetyltransferase	Q5AMQ5	CAALFM_C402020WA	CAT2	1.03	4.6	-2.159	-0.263
Gst2p	Q5AFB4	CAALFM_C402990CA	GST2	2.05	9.04	-2.141	-0.259
Acetylornithine transaminase	A0A1D8PM83	CAALFM_C405070CA	ARG8	4.51	19.87	-2.139	-0.259
37S ribosomal protein S25, mitochondrial	Q5AQ57	CAALFM_C108920WA	RSM25	0.55	2.4	-2.126	-0.256
NADPH--cytochrome P450 reductase	A0A1D8PLR7	CAALFM_C403180WA	NCP1	0.5	2.1	-2.070	-0.243
Glycolipid 2-alpha-mannosyltransferase 1	Q00310	CAALFM_C301810CA	MNT1	2.16	9	-2.059	-0.241
Isocitrate dehydrogenase [NAD] subunit, mitochondrial	A0A1D8PGS5	CAALFM_C203080WA	IDH2	12.09	50.19	-2.054	-0.240
Inosine-5'-monophosphate dehydrogenase	Q59Q46	CAALFM_C206390CA	IMH3	5.89	24.37	-2.049	-0.239
M20_dimer domain-containing protein	A0A1D8PLD9	CAALFM_C401830CA	orf19	1.05	4.34	-2.047	-0.238
Alcohol dehydrogenase 2	O94038	CAALFM_C108330CA	ADH2	20.51	84.29	-2.039	-0.236
Serine hydroxymethyltransferase, cytosolic	O13426	CAALFM_C603760CA	SHM2	9.28	37.52	-2.016	-0.231
Dynamin-like GTPase	A0A1D8PN45	CAALFM_C501210WA	VPS1	0.52	2.09	-2.007	-0.229
Prb1p	Q59Z57	CAALFM_C206880CA	PRB1	1.05	4.19	-1.997	-0.227
Phenylalanine--tRNA ligase subunit beta	A0A1D8PS16	CAALFM_CR01760CA	FRS1	0.52	2.04	-1.972	-0.221
Histone acetyltransferase type B subunit 2	Q59RH5	CAALFM_C604540CA	HAT2	0.52	2.04	-1.972	-0.221
FACT complex subunit POB3	Q5ALL8	CAALFM_C202380WA	POB3	0.5	1.96	-1.971	-0.221
Isocitrate dehydrogenase [NADP]	A0A1D8PHH7	CAALFM_C205890CA	IDP1	7.89	30.39	-1.946	-0.215
Proteasome regulatory particle base subunit	Q5A0L8	CAALFM_C203060WA	PR26	1.08	4.1	-1.925	-0.211
Glutamine--tRNA ligase	A0A1D8PQM9	CAALFM_C700550CA	GLN4	0.5	1.88	-1.911	-0.208
S-adenosylmethionine synthase	A0A1D8PF68	CAALFM_C111450CA	SAM2	15.53	57.15	-1.880	-0.201
Proteasome core particle subunit beta 1	A0A1D8PK85	CAALFM_C305560WA	PRE3	4.07	14.73	-1.856	-0.195
Clathrin heavy chain	A0A1D8PPS9	CAALFM_C602120WA	CHC1	1.53	5.38	-1.814	-0.186
Clustered mitochondria protein homolog	Q59MA9	CAALFM_C104970WA	CLU1	4.1	14.23	-1.795	-0.182
Adenylosuccinate lyase	A0A1D8PT56	CAALFM_CR06150CA	ADE13	5.13	17.72	-1.788	-0.180
Phosphoglucomutase	A0A1D8PSA9	CAALFM_CR02820WA	PGM2	11.8	40.23	-1.770	-0.176
Coatomer subunit beta	Q5A6M6	CAALFM_CR04380CA	SEC26	1	3.39	-1.761	-0.174
Guanine nucleotide-binding protein subunit beta-like protein	P83774	CAALFM_C701250WA	ASC1	30.07	101.07	-1.749	-0.171
Ifg3p	A0A1D8PMX7	CAALFM_C500450CA	IFG3	3.01	10.09	-1.745	-0.171
Isocitrate dehydrogenase [NAD] subunit, mitochondrial	A0A1D8PEM5	CAALFM_C109630WA	IDH1	14.37	46.84	-1.705	-0.161
NADH-ubiquinone reductase	Q5AEC9	CAALFM_C303420CA	NDE1	3.09	10.04	-1.700	-0.160
Proteasome subunit beta	A0A1D8PNC8	CAALFM_C502230WA	orf19	2.05	6.61	-1.689	-0.158
Proteasome core particle subunit beta 3	A0A1D8PRB6	CAALFM_C703390CA	PUP3	4.59	14.73	-1.682	-0.156
Phosphate transporter	A0A1D8PF54	CAALFM_C111480WA	PHO84	5.64	17.95	-1.670	-0.154
26S proteasome regulatory subunit RPN1	A0A1D8PFL7	CAALFM_C113300CA	RPN1	1.61	5.1	-1.663	-0.152
Threonine synthase	A0A1D8PNG9	CAALFM_C502270WA	THR4	6.31	19.8	-1.650	-0.149
Uncharacterized protein	Q59TT2	CAALFM_C111580WA	orf19	1.08	3.37	-1.642	-0.147
Prohibitin	A0A1D8PJT2	CAALFM_C303590WA	orf19	1.53	4.72	-1.625	-0.144
Proteasome regulatory particle base subunit	A0A1D8PI93	CAALFM_C208780WA	RPT6	0.5	1.54	-1.623	-0.143
Glyceraldehyde-3-phosphate dehydrogenase	Q5ADM7	CAALFM_C306870WA	TDH3	163.66	499.05	-1.609	-0.140
Nuc2p	Q5AH07	CAALFM_C701900WA	NUC2	3.19	9.72	-1.607	-0.140
Mitochondrial 37S ribosomal protein NAM9	Q5A6Q6	CAALFM_CR04140WA	CAALFM_CR04140WA	0.5	1.52	-1.604	-0.139
Bifunctional carbamoylphosphate synthetase/aspartate transcarbamylase	A0A1D8PTD1	CAALFM_CR07050CA	URA2	3.62	10.84	-1.582	-0.134
Proteasome endopeptidase complex	Q5AEB8	CAALFM_C303520CA	PRE9	6.12	18.26	-1.577	-0.133
Actin	A0A1D8PFR4	CAALFM_C113700WA	ACT1	24.25	72.25	-1.575	-0.132
RNA export factor	A0A1D8PQE5	CAALFM_C604360CA	GLE2	0.52	1.54	-1.566	-0.130
Ume1p	A0A1D8PRH3	CAALFM_C703960CA	UME1	0.52	1.54	-1.566	-0.130
Agglutinin-like protein 2	P0CU38	CAALFM_C604380WA	ALS2	1.55	4.59	-1.566	-0.130
ATP synthase subunit alpha	A0A1D8PDC4	CAALFM_C104610WA	ATP1	50.18	147.72	-1.558	-0.129
Glucosamine_iso domain-containing protein	Q59YH1	CAALFM_C209970CA	orf19	0.52	1.52	-1.548	-0.126
Tubulin beta chain	A0A1D8PC97	CAALFM_C100710CA	TUB2	4.2	12.12	-1.529	-0.122
Tubulin alpha chain	A0A1D8PTV4	CAALFM_CR09120CA	TUB1	3.09	8.9	-1.526	-0.121
Arginine biosynthesis bifunctional protein ArgJ, mitochondrial	Q5AH38	CAALFM_C702150CA	ECM42	2.15	6.11	-1.507	-0.117
Uncharacterized protein	A0A1D8PPV4	CAALFM_C602300CA	orf19	0.5	1.42	-1.506	-0.117
Asparagine--tRNA ligase	Q59R18	CAALFM_C703670WA	DED81	7.37	20.8	-1.497	-0.115
Orotate phosphoribosyltransferase	A0A1D8PS11	CAALFM_CR01650WA	URA5	2.05	5.76	-1.490	-0.113
Pyruvate dehydrogenase E1 component subunit alpha	Q5A0Z9	CAALFM_C407110CA	PDA1	9.38	26.13	-1.478	-0.111
S-formylglutathione hydrolase	A0A1D8PU04	CAALFM_CR09670CA	CAALFM_CR09670CA	3.11	8.66	-1.478	-0.111
NAD-specific glutamate dehydrogenase	A0A1D8PI00	CAALFM_C207900WA	GDH2	14.91	41.09	-1.463	-0.107
Uncharacterized protein	A0A1D8PDL7	CAALFM_C105630CA	orf19	2.06	5.65	-1.456	-0.106
Eukaryotic translation initiation factor 3 subunit A	Q59PL9	CAALFM_C112770WA	RPG1A	1.57	4.3	-1.454	-0.105
Etr1p	A0A1D8PIW1	CAALFM_C300200CA	ETR1	28.09	76.71	-1.449	-0.104
CAAX prenyl protease	A0A1D8PL01	CAALFM_C400280WA	STE24	0.52	1.42	-1.449	-0.104
Uncharacterized protein	A0A1D8PP33	CAALFM_C504950CA	orf19	1.05	2.85	-1.441	-0.102
Adh1p	A0A1D8PP43	CAALFM_C505050WA	ADH1	247.07	669.3	-1.438	-0.102
Transketolase	Q5A750	CAALFM_C108320WA	TKL1	26.62	71.9	-1.434	-0.101
Diphosphomevalonate decarboxylase	A0A1D8PC43	CAALFM_C100070WA	MVD	1.61	4.34	-1.431	-0.100

Multifunctional fusion protein	Q5AK46	CAALFM_C504880CA	PUT2	5.66	15.14	-1.420	-0.098
Peroxisomal catalase	O13289	CAALFM_C106810WA	CAT1	40.95	108.34	-1.404	-0.094
Elongation factor G, mitochondrial	Q5AL45	CAALFM_C113060CA	MEF1	1.56	4.1	-1.394	-0.092
DLH domain-containing protein	A0A1D8PLD7	CAALFM_C401840CA	orf19	21.91	57.57	-1.394	-0.092
Ubiquinol--cytochrome-c reductase catalytic subunit	A0A1D8PHA3	CAALFM_C204950CA	CYT1	3.6	9.43	-1.389	-0.091
Proteasome core particle subunit beta 2	A0A1D8PU67	CAALFM_CR10300WA	PUP1	5.1	13.21	-1.373	-0.087
Deoxyhypusine synthase	A0A1D8PJD2	CAALFM_C302180CA	orf19	0.55	1.42	-1.368	-0.086
Proline--tRNA ligase	Q59R20	CAALFM_C703660CA	orf19	5.72	14.71	-1.363	-0.085
Alpha-ketoglutarate dehydrogenase	A0A1D8PJ26	CAALFM_C300880WA	KGD1	7.75	19.89	-1.360	-0.084
4-aminobutyrate transaminase	A0A1D8PH55	CAALFM_C204190CA	UGA1	3.57	9.14	-1.356	-0.083
Csh1p	Q59QH2	CAALFM_C104020CA	CSH1	2.05	5.24	-1.354	-0.083
Translation initiation factor eIF2 subunit gamma	Q5AGF6	CAALFM_C502170CA	GCD11	3.22	8.23	-1.354	-0.083
Fatty acid synthase subunit beta	Q5A4W7	CAALFM_C500190CA	FAS1	12.09	30.83	-1.351	-0.082
Acetyl-CoA C-acetyltransferase	A0A1D8PH52	CAALFM_C204310WA	ERG10	39.76	101.26	-1.349	-0.082
Glucose-6-phosphate 1-dehydrogenase	A0A1D8PEG2	CAALFM_C108980CA	ZWF1	6.32	16.01	-1.341	-0.080
Sfc1p	A0A1D8PNY6	CAALFM_C504440CA	SFC1	1.61	4.07	-1.338	-0.079
Malate synthase	Q5APD2	CAALFM_C109690WA	MLS1	13.88	34.96	-1.333	-0.078
Chaperonin-containing T-complex subunit	Q5AB74	CAALFM_C100110WA	CCT8	1.63	4.1	-1.331	-0.078
Aspartate aminotransferase	A0A1D8PL85	CAALFM_C401200CA	AAT22	17.41	43.7	-1.328	-0.077
Dolichol-phosphate mannosyltransferase subunit 1	A0A1D8PEA2	CAALFM_C108010WA	DPM1	1.05	2.62	-1.319	-0.075
Ribonucleotide-diphosphate reductase subunit	Q5A0L0	CAALFM_C203010CA	RNR21	3.16	7.85	-1.313	-0.074
Ribosomal 60S subunit protein L5	Q5AGZ7	CAALFM_C701790CA	RPL5	18.01	44.74	-1.313	-0.074
Pex7p	Q59WW3	CAALFM_C600880WA	PEX7	6.14	15.24	-1.312	-0.073
Psa2p	Q5AL34	CAALFM_C113160WA	PSA2	5.77	14.19	-1.298	-0.070
Glucose-6-phosphate 1-epimerase	Q5A1Q0	CAALFM_C501230CA	orf19	1.07	2.62	-1.292	-0.069
SEC14 cytosolic factor	P46250	CAALFM_C500480CA	SEC14	1.57	3.84	-1.290	-0.069
Trifunctional aldehyde reductase/xylose reductase/glucose 1-dehydrogenase	A0A1D8PNK3	CAALFM_C502930CA	GRE3	10.63	25.89	-1.284	-0.067
Uncharacterized protein	Q5AD19	CAALFM_C200760CA	orf19	4.21	10.18	-1.274	-0.065
Uncharacterized protein	A0A1D8PDH0	CAALFM_C105160CA	orf19	1.05	2.53	-1.269	-0.064
UTP--glucose-1-phosphate uridylyltransferase	Q59KI0	CAALFM_CR04660CA	UGP1	5.22	12.57	-1.268	-0.064
Protein FMP52, mitochondrial	Q5AP65	CAALFM_C110280CA	FMP52	2.56	6.15	-1.264	-0.063
Regulator of cytoskeleton and endocytosis RVS161	Q5AFE4	CAALFM_C700020CA	RVS161	1.6	3.84	-1.263	-0.062
PALP domain-containing protein	A0A1D8PRJ1	CAALFM_C704210CA	orf19	9.75	23.37	-1.261	-0.062
Adh5p	A0A1D8PS45	CAALFM_CR02070CA	ADH5	9.17	21.81	-1.250	-0.060
3-isopropylmalate dehydratase	A0A1D8PRP8	CAALFM_CR00360CA	LEU1	1.02	2.41	-1.241	-0.057
Rab family GTPase	A0A1D8PTI2	CAALFM_CR07520CA	YPT31	1.08	2.55	-1.240	-0.057
NADH dehydrogenase [ubiquinone] flavoprotein 1, mitochondrial	Q59TD5	CAALFM_C204550CA	NDH51	2.59	6.1	-1.236	-0.056
Alpha,alpha-trehalose-phosphate synthase [UDP-forming]	Q92410	CAALFM_CR05720WA	TPS1	2.61	6.11	-1.227	-0.054
Bifunctional aminoimidazole ribotide synthase/glycinamide ribotide synthase	A0A1D8PE67	CAALFM_C107890CA	ADE5	2.06	4.82	-1.226	-0.054
Aspartate aminotransferase	Q59N40	CAALFM_CR07620WA	AAT21	27.58	63.99	-1.214	-0.052
Mannose-1-phosphate guanyltransferase	O93827	CAALFM_C307950CA	MPG1	9.43	21.86	-1.213	-0.051
Adenosylhomocysteinase	P83783	CAALFM_C504270CA	SAH1	24.66	56.98	-1.208	-0.050
Branched-chain-amino-acid aminotransferase	A0A1D8PKB9	CAALFM_C305590CA	BAT22	15	34.58	-1.205	-0.049
Arginine--tRNA ligase	A0A1D8PCI5	CAALFM_C101530CA	orf19	2.05	4.71	-1.200	-0.048
Dihydroorotate dehydrogenase	Q874I4	CAALFM_C109720WA	URA9	2.1	4.82	-1.199	-0.048
Valine--tRNA ligase	A0A1D8PHR0	CAALFM_C206640CA	VAS1	6.52	14.96	-1.198	-0.048
Glutamine synthetase	A0A1D8PSY1	CAALFM_CR05050WA	GLN1	2.12	4.86	-1.197	-0.048
Glutathione reductase	Q59NQ5	CAALFM_C501520CA	GLR1	6.66	15.23	-1.193	-0.047
Aspartate--tRNA ligase	Q59UF7	CAALFM_CR03170WA	DPS1-1	8.28	18.83	-1.185	-0.045
Proteasome endopeptidase complex	A0A1D8PQW1	CAALFM_C701470CA	PRE10	5.14	11.68	-1.184	-0.045
Mitochondrial 54S ribosomal protein YmL11	Q5A693	CAALFM_C404820CA	orf19	1.07	2.41	-1.171	-0.042
Proteasome regulatory particle base subunit	A0A1D8PIV7	CAALFM_C300290WA	RPT2	1.08	2.41	-1.158	-0.039
Uncharacterized protein	Q5A4L1	CAALFM_C601560WA	orf19	1.08	2.4	-1.152	-0.038
Thioredoxin reductase	Q5AG89	CAALFM_C502710WA	TRR1	1.08	2.4	-1.152	-0.038
Glyco_hydro_63 domain-containing protein	Q5AED0	CAALFM_C303410CA	orf19	6.82	15.14	-1.151	-0.037
Bifunctional hydroxyacyl-CoA dehydrogenase/enoyl-CoA hydratase	A0A1D8PJ13	CAALFM_C300810CA	FOX2	4.63	10.24	-1.145	-0.036
Proteasome endopeptidase complex	A0A1D8PSS4	CAALFM_CR04550WA	PRE6	4.6	10.11	-1.136	-0.034
Chaperonin-containing T-complex subunit	Q59YC4	CAALFM_C209520CA	CCT2	1.07	2.35	-1.135	-0.034
Serine/threonine-protein phosphatase	Q59N42	CAALFM_CR07650WA	GLC7	4.21	9.16	-1.122	-0.031
Homoisocitrate dehydrogenase	Q5A9D9	CAALFM_CR01400WA	LYS12	29.91	64.87	-1.117	-0.030
Proteinase A	Q59U59	CAALFM_C207400CA	4/1/2021	10.52	22.6	-1.103	-0.027
Flavohemoprotein	Q59MV9	CAALFM_CR07790CA	YHB1	3.57	7.65	-1.100	-0.026
Leu42p	A0A1D8PIF8	CAALFM_C209750WA	LEU42	5.37	11.39	-1.085	-0.022
Mitochondrial 37S ribosomal protein MRP4	A0A1D8PQ89	CAALFM_C603730CA	orf19	0.5	1.05	-1.070	-0.019
Cysteine--tRNA ligase	Q5AL46	CAALFM_C113030CA	orf19	0.5	1.05	-1.070	-0.019
Enolase 1	P30575	CAALFM_C108500CA	ENO1	376.27	784.99	-1.061	-0.017
Glycerol 2-dehydrogenase	Q5ADT4	CAALFM_C307340WA	GCY1	5.41	11.27	-1.059	-0.017
40S ribosomal protein S27	A0A1D8PDP4	CAALFM_C105510CA	RPS27A	0.5	1.04	-1.057	-0.016
Pyruvate decarboxylase	P83779	CAALFM_C406570CA	PDC11	87.2	180.98	-1.053	-0.015
Tom40p	Q5AH14	CAALFM_C701970CA	TOM40	2.56	5.31	-1.053	-0.015
Cytochrome c oxidase subunit 2	Q9B8D8	CaalfMp01	COX2	2.05	4.25	-1.052	-0.015
Mir1p	Q5AP79	CAALFM_C110160WA	MIR1	11.91	24.68	-1.051	-0.015
Pentafunctional AROM polypeptide	Q5AME2	CAALFM_C400890WA	ARO1	1.02	2.1	-1.042	-0.013
Dipeptidyl peptidase 3	A0A1D8PQB4	CAALFM_C603960WA	orf19	3.68	7.55	-1.037	-0.012
Acyl-protein thioesterase 1	Q5AGD1	CAALFM_C502400WA	CAALFM_C502400WA	2.12	4.34	-1.034	-0.011
Alanine--tRNA ligase	Q5A8K2	CAALFM_C603720WA	ALA1	5.19	10.61	-1.032	-0.011
Uncharacterized protein	A0A1D8PMU2	CAALFM_C500100CA	orf19	2.14	4.37	-1.030	-0.010
Aspartate--tRNA ligase	A0A1D8PD81	CAALFM_C104030WA	orf19	0.5	1.02	-1.029	-0.010
Nicotinamide-nucleotide adenylyltransferase	A0A1D8PRN2	CAALFM_CR00350WA	CAALFM_CR00350WA	0.5	1.02	-1.029	-0.010
tRNA (Cytosine-5-)-methyltransferase	A0A1D8PSR2	CAALFM_CR04300WA	CAALFM_CR04300WA	0.5	1.02	-1.029	-0.010
NADH-ubiquinone oxidoreductase chain 5	Q9B8C9	CaalfMp13	NAD5	0.5	1.02	-1.029	-0.010
Citrate synthase	A0A1D8PSH3	CAALFM_CR03500WA	CIT1	23.7	48.33	-1.028	-0.010
Ddi1p	A0A1D8PFX6	CAALFM_C114420WA	DDI1	1.03	2.1	-1.028	-0.010
Acetolactate synthase	A0A1D8PJF9	CAALFM_C302320WA	ILV2	4.84	9.86	-1.027	-0.009
Mdj1p	A0A1D8PNQ8	CAALFM_C503600WA	MDJ1	0.52	1.05	-1.014	-0.007
Mitochondrial import inner membrane translocase subunit TIM44	Q5A7M2	CAALFM_C300250CA	TIM44	0.52	1.05	-1.014	-0.007
Transcriptional repressor TUP1	P0CY34	CAALFM_C100060WA	TUP1	38.11	76.8	-1.011	-0.006
Protein transport protein SEC24	Q5AQ76	CAALFM_C108740CA	SEC24	1.02	2.05	-1.007	-0.005
Phenylalanine--tRNA ligase subunit alpha	A0A1D8PCT4	CAALFM_C102710WA	FRS2	1.02	2.04	-1.000	-0.003
ADP/ATP carrier protein	Q5A516	CAALFM_C500590WA	PET9	31.6	63.06	-0.997	-0.003
ATPase GET3	P0CB54	CAALFM_C102760WA	GET3	1.55	3.05	-0.977	0.002
Isocitrate dehydrogenase [NADP]	A0A1D8PS79	CAALFM_CR02360WA	IDP2	11.59	22.79	-0.976	0.002
F-actin-capping protein subunit beta	Q5AMP9	CAALFM_C401950WA	orf19	0.52	1.02	-0.972	0.003
Phosphatidylinositol-binding protein	A0A1D8PQC2	CAALFM_C604100WA	orf19	0.52	1.02	-0.972	0.003
Uncharacterized protein	A0A1D8PU57	CAALFM_CR10200WA	CAALFM_CR10200WA	0.52	1.02	-0.972	0.003

Mitochondrial 54S ribosomal protein YmL8	Q59RQ4	CAALFM_CR07390CA	MRPL8	0.52	1.02	-0.972	0.003
Protein phosphatase 2A structural subunit	Q5ADN1	CAALFM_C306910CA	TPD3	0.52	1.02	-0.972	0.003
Aha1p	A0A1D8PU72	CAALFM_CR10270CA	AHA1	1.57	3.07	-0.968	0.004
Bifunctional (2E,6E)-farnesyl diphosphate synthase/dimethylallyltranstransferase	A0A1D8PH78	CAALFM_C204580WA	ERG20	1.61	3.14	-0.964	0.005
Yhm2p	Q59NH3	CAALFM_C600600CA	YHM2	1.05	2.04	-0.958	0.006
Sphingolipid C9-methyltransferase	Q5APD4	CAALFM_C109680WA	MTS1	1.57	3.05	-0.958	0.006
Ketol-acid reductoisomerase, mitochondrial	A0A1D8PPG7	CAALFM_C600870CA	ILV5	14.44	28.03	-0.957	0.006
AA_permease domain-containing protein	A0A1D8PU37	CAALFM_CR09920WA	CAALFM_CR09920WA	1.57	3.03	-0.949	0.008
Fatty acid synthase subunit alpha	A0A1D8PK65	CAALFM_C304830CA	FAS2	15.97	30.59	-0.938	0.011
Acetyl-coenzyme A synthetase 2	Q8NJN3	CAALFM_C104290CA	ACS2	7.77	14.88	-0.937	0.011
Fumarate reductase	Q59T35	CAALFM_C205700WA	OSM1	15.33	29.27	-0.933	0.012
ANK_REP_REGION domain-containing protein	A0A1D8PQI0	CAALFM_C700140CA	orf19	1.53	2.92	-0.932	0.012
E1 ubiquitin-activating protein	A0A1D8PKJ3	CAALFM_C306500WA	UBA1	3.57	6.81	-0.932	0.012
Ino4p	A0A1D8PH18	CAALFM_C203840CA	INO4	3.27	6.22	-0.928	0.013
Uncharacterized protein	A0A1D8PHU4	CAALFM_C207010WA	orf19	1.02	1.94	-0.928	0.013
Cystathionine beta-synthase	Q59T95	CAALFM_C101870CA	CYS4	3.07	5.8	-0.918	0.015
NADH dehydrogenase [ubiquinone] 1 alpha subcomplex subunit	Q5AJG7	CAALFM_C302190CA	orf19	1.08	2.04	-0.918	0.015
Metalloendopeptidase	Q5A2A7	CAALFM_C105300CA	PRD1	4.61	8.7	-0.916	0.015
Oxidoreductase	Q5AML3	CAALFM_C401510WA	orf19	4.63	8.73	-0.915	0.016
Yim1p	Q5AHE9	CAALFM_C203750WA	YIM1	1	1.88	-0.911	0.017
Ife2p	A0A1D8PSZ0	CAALFM_CR05340CA	IFE2	7.37	13.76	-0.901	0.019
ATP-dependent RNA helicase DED1	Q5A4E2	CAALFM_C306100CA	DED1	1.02	1.9	-0.897	0.020
Heat shock protein 78, mitochondrial	Q96UX5	CAALFM_C203390CA	HSP78	3.11	5.77	-0.892	0.021
CBM21 domain-containing protein	Q5A932	CAALFM_C101140CA	orf19	0.55	1.02	-0.891	0.021
T-complex protein 1 subunit gamma	Q5AK16	CAALFM_C505120WA	CCT3	0.55	1.02	-0.891	0.021
V-type proton ATPase subunit B	Q59PT0	CAALFM_CR05780WA	VMA2	9.76	18.07	-0.889	0.022
Proteasome regulatory particle lid subunit	A0A1D8PTV0	CAALFM_CR08910CA	RPN7	2.06	3.8	-0.883	0.023
Eukaryotic translation initiation factor 3 subunit C	Q5AML1	CAALFM_C401490WA	NIP1	5.27	9.68	-0.877	0.024
Peptide-methionine-S-sulfoxide reductase	Q5AD39	CAALFM_C200960CA	MXR1	2.57	4.72	-0.877	0.024
F1F0 ATP synthase subunit k	A0A1D8PFD4	CAALFM_C112320CA	ATP19	0.53	0.97	-0.872	0.025
Uncharacterized protein	A0A1D8PEH4	CAALFM_C109020WA	orf19	2.05	3.75	-0.871	0.025
Dynamin-related GTPase	A0A1D8PKC9	CAALFM_C305520CA	DNM1	1.04	1.9	-0.869	0.026
Small COPII coat GTPase SAR1	Q59S78	CAALFM_C602220WA	SAR1	2.67	4.86	-0.864	0.027
ATP synthase subunit beta	A0A1D8PKZ9	CAALFM_C400270WA	ATP2	118.25	214.78	-0.861	0.028
Uncharacterized protein	Q5AI75	CAALFM_C102890CA	orf19	1.05	1.9	-0.856	0.029
Obg-like ATPase 1	A0A1D8PDE8	CAALFM_C104890WA	YBN5	4.33	7.83	-0.855	0.029
Inosine triphosphate pyrophosphatase	Q59N80	CAALFM_C503860WA	HAM1	2.63	4.75	-0.853	0.030
Carnitine:acyl carnitine antiporter	Q5A967	CAALFM_CR01980CA	CRC1	1.61	2.89	-0.844	0.032
Pin4p	A0A1D8PRZ6	CAALFM_CR01500WA	PIN4	0.53	0.95	-0.842	0.032
Uroporphyrinogen decarboxylase	Q5A5N0	CAALFM_C210860CA	orf19	2.55	4.57	-0.842	0.032
Rab GDP dissociation inhibitor	A0A1D8PFX8	CAALFM_C114440CA	GDI1	1.6	2.86	-0.838	0.033
Acetyl-CoA C-acyltransferase	A0A1D8PRL6	CAALFM_CR00150CA	POT1	8.41	14.91	-0.826	0.036
Acetolactate synthase regulatory subunit	A0A1D8PLB7	CAALFM_C401370WA	ILV6	5.62	9.95	-0.824	0.036
Adenylosuccinate synthetase	P0CH96	CAALFM_C109640WA	ADE12	7.07	12.5	-0.822	0.036
Uncharacterized protein	Q59MG4	CAALFM_C205810WA	orf19	0.55	0.97	-0.819	0.037
Nop13p	Q5ADS5	CAALFM_C307300WA	NOP13	1.08	1.9	-0.815	0.038
Bifunctional 2-aminoadipate transaminase/aromatic-amino-acid:2-oxoglutarate transaminase	A0A1D8PG20	CAALFM_C200340CA	ARO8	7.68	13.47	-0.811	0.039
ATP-dependent 6-phosphofructokinase	Q5AK53	CAALFM_C504810WA	PFK1	12.97	22.67	-0.806	0.040
Uridylate kinase	Q59KZ3	CAALFM_C104420CA	URA6	5.1	8.91	-0.805	0.040
Ribosomal 60S subunit protein L3	Q59LS1	CAALFM_C209430WA	RPL3	26.78	46.75	-0.804	0.041
Cdr4p	A0A1D8PE84	CAALFM_C108070WA	CDR4	6.12	10.68	-0.803	0.041
Uncharacterized protein	A0A1D8PG37	CAALFM_C200400CA	orf19	5.12	8.92	-0.801	0.041
Putative hydrolase	Q59WF0	CAALFM_C110700CA	NIT3	1.08	1.88	-0.800	0.042
Copper metallochaperone	A0A1D8PGA3	CAALFM_C201180WA	COX17	2.06	3.58	-0.797	0.042
Riboflavin synthase	A0A1D8PP67	CAALFM_C505300WA	RIB5	2.72	4.72	-0.795	0.043
Ribosomal 60S subunit protein L21A	A0A1D8PGY0	CAALFM_C203810CA	RPL21A	6.83	11.84	-0.794	0.043
Alpha-1,4 glucan phosphorylase	A0A1D8PQQ3	CAALFM_C700930WA	GPH1	19.66	34.03	-0.792	0.043
Cam1-1p	A0A1D8PNN8	CAALFM_C503280WA	CAM1-1	9.19	15.9	-0.791	0.043
Cu-binding protein	A0A1D8PTX2	CAALFM_CR09300CA	SCO1	1.52	2.62	-0.786	0.045
Ribosomal 60S subunit protein L22B	A0A1D8PM41	CAALFM_C404390WA	orf19	21.42	36.54	-0.771	0.048
Ribosomal 60S subunit protein L11B	A0A1D8PHW1	CAALFM_C206810CA	RPL11	5.12	8.68	-0.762	0.050
Mitochondrial import inner membrane translocase subunit TIM23	Q59YG5	CAALFM_C209900CA	TIM23	2.1	3.55	-0.757	0.051
Sepiapterin reductase family protein	A0A1D8PDA0	CAALFM_C104430CA	orf19	1.53	2.58	-0.754	0.052
ATP synthase subunit gamma	A0A1D8PRY3	CAALFM_CR01310WA	ATP3	28.91	48.73	-0.753	0.052
Acetyl-coenzyme A synthetase	Q59XW4	CAALFM_C210350CA	ACS1	6.22	10.48	-0.753	0.052
40S ribosomal protein S26	Q5ALV6	CAALFM_C201610CA	RPS26A	4.88	8.21	-0.751	0.053
Succinate dehydrogenase [ubiquinone] iron-sulfur subunit, mitochondrial	Q59QN7	CAALFM_CR05180CA	SDH2	3.16	5.31	-0.749	0.053
Leucine--tRNA ligase	A0A1D8PS12	CAALFM_CR01690CA	CDC60	5.14	8.62	-0.746	0.054
Ornithine aminotransferase	A0A1D8PL14	CAALFM_C400160CA	CAR2	33.72	56.46	-0.744	0.054
Sps20p	Q59SL5	CAALFM_C400830WA	SPS20	1.53	2.55	-0.737	0.056
FAA_hydrolase domain-containing protein	A0A1D8PFV6	CAALFM_C114320CA	orf19	1.53	2.53	-0.726	0.058
V-type proton ATPase subunit a	Q59R99	CAALFM_C405240CA	VPH1	2.15	3.55	-0.724	0.059
Glycine--tRNA ligase	Q5A2A5	CAALFM_C105290WA	GRS1	7.75	12.74	-0.717	0.060
Rab family GTPase	Q5AI00	CAALFM_C103500WA	YPT1	4.13	6.78	-0.715	0.060
Elongation factor 2	Q5A0M4	CAALFM_C203100WA	EFT2	68.11	111.49	-0.711	0.061
Altered inheritance of mitochondria protein 9, mitochondrial	Q5A922	CAALFM_C101250WA	AIM9	1.56	2.55	-0.709	0.062
Protein transport protein SEC31	Q5AAU3	CAALFM_C106930WA	PGA63	9.2	15.03	-0.708	0.062
Co-chaperone	A0A1D8PIG5	CAALFM_C209270CA	SGT1	1.55	2.53	-0.707	0.062
NAD(P)-bd_dom domain-containing protein	A0A1D8PJA6	CAALFM_C301610WA	orf19	4.63	7.55	-0.706	0.063
Trehalose 6-phosphate synthase/phosphatase complex subunit	A0A1D8PIS4	CAALFM_C210690WA	TPS3	2.57	4.19	-0.705	0.063
V-type proton ATPase catalytic subunit A	Q5AJB1	CAALFM_C301630WA	TFP1	7.22	11.7	-0.696	0.065
Ali1p	A0A1D8PJ73	CAALFM_C301410CA	ALI1	4.09	6.6	-0.690	0.066
Isoleucine--tRNA ligase	Q59RI1	CAALFM_C604520WA	ILS1	8.19	13.1	-0.678	0.069
3-hydroxy-3-methylglutaryl coenzyme A synthase	A0A1D8PTW6	CAALFM_CR09160CA	ERG13	6.97	11.14	-0.677	0.069
Isocitrate lyase	Q59RB8	CAALFM_C104500WA	ICL1	11.26	17.95	-0.673	0.070
Tryptophan synthase	A0A1D8PMH6	CAALFM_C406110CA	TRP5	2.07	3.3	-0.673	0.070
Threonine--tRNA ligase	A0A1D8PMV9	CAALFM_C500110CA	THS1	4.27	6.8	-0.671	0.070
Translation termination factor eRF1	Q59ZH8	CAALFM_C205100CA	ERF1	2.11	3.35	-0.667	0.071
Eukaryotic translation initiation factor 3 subunit B	Q5AGV4	CAALFM_C701450CA	PRT1	2.11	3.35	-0.667	0.071
Elongation factor 1-alpha 1	P0CY35	CAALFM_C208370CA	TEF1	88.65	140.5	-0.664	0.072
Asparagine synthase	A0A1D8PIB2	CAALFM_C209060CA	ASN1	5.16	8.15	-0.659	0.073
Adenine phosphoribosyltransferase	Q5ALX8	CAALFM_C201430WA	APT1	3.68	5.77	-0.649	0.075
Saccharopine dehydrogenase	A0A1D8PKJ4	CAALFM_C306590WA	LYS9	8.66	13.55	-0.646	0.076
Dihydroxyacetone kinase	A0A1D8PEI2	CAALFM_C109190CA	DAK2	14.94	23.33	-0.643	0.077
Ribosomal 40S subunit protein S29A	A0A1D8PTR4	CAALFM_CR08480CA	CAALFM_CR08480CA	2.61	4.05	-0.634	0.079

Uncharacterized protein	A0A1D8PU54	CAALFM_CR10130WA	CAALFM_CR10130WA	1.55	2.4	-0.631	0.079
Ribosomal protein L15	Q5A6R1	CAALFM_CR04100CA	RPL15A	9.21	14.26	-0.631	0.079
Aconitate hydratase, mitochondrial	P82611	CAALFM_CR08210CA	ACO1	44.41	68.68	-0.629	0.080
37S ribosomal protein S9, mitochondrial	O94150	CAALFM_C112340CA	MRPS9	1.02	1.57	-0.622	0.081
Leucine aminopeptidase 2	Q59NB8	CAALFM_C110490WA	LKH1	1.02	1.57	-0.622	0.081
Ferroxidase	Q59PA3	CAALFM_C404340CA	YFH1	1.02	1.57	-0.622	0.081
CRAL-TRIO domain-containing protein	A0A1D8PQ60	CAALFM_C603470WA	orf19	1.02	1.57	-0.622	0.081
Cys-Gly metallodipeptidase DUG1	Q5AKA5	CAALFM_C504300CA	DUG1	6.72	10.17	-0.598	0.087
6-phosphogluconate dehydrogenase, decarboxylating	A0A1D8PFS4	CAALFM_C113860CA	GND1	41.67	63.06	-0.598	0.087
Pyruvate dehydrogenase E1 component subunit beta	Q5A5V6	CAALFM_C404150CA	PDB1	11.91	17.99	-0.595	0.087
Bifunctional phosphoribosylaminoimidazolecarboxamide formyltransferase/IMP cyclohydrolase	Q5A6R2	CAALFM_CR04090CA	ADE17	22.81	34.41	-0.593	0.088
Translation elongation factor EF1B gamma	A0A1D8PKC3	CAALFM_C306010WA	CAM1	13.52	20.35	-0.590	0.089
Adenylyl cyclase-associated protein	Q5A6P9	CAALFM_CR04190WA	SRV2	5.22	7.85	-0.589	0.089
L-iditol 2-dehydrogenase	A0A1D8PUB4	CAALFM_CR10840CA	XYL2	22.71	34.03	-0.584	0.090
Omega-class glutathione transferase	A0A1D8PS56	CAALFM_CR02130WA	ECM4	15.81	23.69	-0.583	0.090
Fimbrin	A0A1D8PPY8	CAALFM_C602730WA	SAC6	6.19	9.27	-0.583	0.090
Importin subunit alpha	A0A1D8PMU8	CAALFM_C500150CA	orf19	1.05	1.57	-0.580	0.091
Succinate dehydrogenase [ubiquinone] flavoprotein subunit, mitochondrial	Q5A1E8	CAALFM_C406610CA	SDH12	27.63	41.21	-0.577	0.092
Cell division control protein 10	P39827	CAALFM_CR04570CA	CDC10	1.02	1.52	-0.576	0.092
Uncharacterized protein	A0A1D8PI37	CAALFM_C207540WA	orf19	1.02	1.52	-0.576	0.092
Phosphoserine aminotransferase	Q59P52	CAALFM_C206210CA	SER1	4.09	6.09	-0.574	0.092
Mitochondrial import inner membrane translocase subunit TIM50	Q59W44	CAALFM_C111220CA	TIM50	2.05	3.05	-0.573	0.092
Proliferating cell nuclear antigen	Q5AMN0	CAALFM_C401770WA	POL30	10.63	15.75	-0.567	0.094
Mitochondrial pyruvate carrier	Q5A328	CAALFM_CR08610WA	CAALFM_CR08610WA	1.05	1.55	-0.562	0.095
Ribosomal 40S subunit protein S2	Q5A900	CAALFM_C101480CA	RPS21	14.41	21.25	-0.560	0.095
Alpha-mannosidase	Q5AF38	CAALFM_C402360WA	AMS1	3.57	5.24	-0.554	0.097
Trans-2-enoyl-CoA reductase	A0A1D8PCD8	CAALFM_C101050CA	orf19	1.05	1.54	-0.553	0.097
Separase	A0A1D8PCM0	CAALFM_C101670CA	ESP1	1.05	1.54	-0.553	0.097
D-3-phosphoglycerate dehydrogenase	Q5A3K7	CAALFM_C112030WA	SER33	7.16	10.48	-0.550	0.098
ATP-dependent RNA helicase eIF4A	P87206	CAALFM_C101350CA	TIF1	13.94	20.35	-0.546	0.098
Uncharacterized protein	A0A1D8PCU5	CAALFM_C102650WA	orf19	1.08	1.57	-0.540	0.100
Putative ATP-dependent permease	A0A1D8PT53	CAALFM_CR05950CA	ADP1	3.62	5.24	-0.534	0.101
Adenosine 5'-monophosphoramidase	Q59WG0	CAALFM_C110780CA	HNT1	9.47	13.7	-0.533	0.101
Gtt11p	A0A1D8PJR2	CAALFM_C303720WA	GTT11	2.11	3.05	-0.532	0.102
26S proteasome regulatory subunit RPN2	Q5A3L0	CAALFM_C112050WA	RPN2	2.15	3.07	-0.514	0.106
Ribosomal 40S subunit protein S16A	A0A1D8PCW6	CAALFM_C103030WA	RPS16A	9.99	14.24	-0.511	0.106
Translation initiation factor eIF5B	Q5A782	CAALFM_C108090CA	FUN12	2.14	3.05	-0.511	0.106
Protein YOP1	A0A1D8PI76	CAALFM_C208160CA	orf19	2.16	3.07	-0.507	0.107
Ribosomal 40S subunit protein S9B	A0A1D8PGY8	CAALFM_C203820CA	RPS9B	11.59	16.46	-0.506	0.107
60S ribosomal protein L10a	Q9UVJ4	CAALFM_C602240CA	RPL10A	11.35	16.09	-0.504	0.108
E2 ubiquitin-conjugating protein	A0A1D8PU40	CAALFM_CR09970WA	UBC4	2.16	3.05	-0.498	0.109
Mitochondrial outer membrane protein porin	P83781	CAALFM_C104100CA	POR1	22.16	31.24	-0.495	0.110
ATP-dependent 6-phosphofructokinase	Q5AGZ8	CAALFM_C701800CA	PFK2	10.78	15.19	-0.495	0.110
Protein channel	Q59LZ5	CAALFM_C702640WA	TOM70	10.75	15.13	-0.493	0.110
Aspartate transaminase	A0A1D8PHC9	CAALFM_C205250CA	AAT1	43.89	61.59	-0.489	0.111
Ribosomal 40S subunit protein S13	A0A1D8PPE0	CAALFM_C600650CA	RPS13	8.71	12.22	-0.489	0.111
Saccharopine dehydrogenase [NAD	P43065	CAALFM_C405320WA	LYS1	4.73	6.63	-0.487	0.112
AAA family ATPase	Q59WG3	CAALFM_C110790WA	CDC48	26.81	37.49	-0.484	0.112
Translation initiation factor eIF2 subunit alpha	Q5AAU7	CAALFM_C106960WA	SUI2	4.09	5.7	-0.479	0.113
MHD domain-containing protein	A0A1D8PL12	CAALFM_C400420CA	orf19	2.11	2.94	-0.479	0.114
Ribosome assembly factor mrt4	Q5AC00	CAALFM_C602770WA	MRT4	1.52	2.1	-0.466	0.116
Putative phosphotransferase	Q5AF71	CAALFM_C402620CA	orf19	6.27	8.64	-0.463	0.117
Bifunctional cysteine synthase/O-acetylhomoserine aminocarboxypropyltransferase	Q59US5	CAALFM_C400200CA	MET15	131.05	180.33	-0.461	0.118
Phosphoglycerate mutase	P82612	CAALFM_C203270WA	GPM1	118.96	163.11	-0.455	0.119
60S ribosomal protein L29	A0A1D8PF57	CAALFM_C111040WA	RPL29	3.74	5.12	-0.453	0.119
Cip1p	A0A1D8PPI6	CAALFM_C601070CA	CIP1	2.61	3.57	-0.452	0.120
Proteasome endopeptidase complex	Q59PZ1	CAALFM_CR06750CA	PUP2	13.76	18.8	-0.450	0.120
Ribosomal 60S subunit protein L18A	A0A1D8PK43	CAALFM_C305100CA	RPL18	14.97	20.43	-0.449	0.120
Glucose-6-phosphate isomerase	P83780	CAALFM_CR06340CA	PGI1	30.44	41.48	-0.446	0.121
Uncharacterized protein	A0A1D8PNP4	CAALFM_C503490CA	orf19	1.05	1.43	-0.446	0.121
Aldedh domain-containing protein	Q5AI07	CAALFM_C103440CA	orf19	1.05	1.43	-0.446	0.121
40S ribosomal protein S4	A0A1D8PCI6	CAALFM_C101640WA	RPS42	20.6	28.05	-0.445	0.121
Slr1p	A0A1D8PIP0	CAALFM_C210290WA	SLR1	2.16	2.94	-0.445	0.121
Lys22p	Q59TC4	CAALFM_C204460WA	LYS22	5.63	7.65	-0.442	0.122
Mitochondrial 54S ribosomal protein YmL13	Q5A3N5	CAALFM_C112280CA	orf19	4.3	5.83	-0.439	0.122
RNA-binding protein	A0A1D8PFX4	CAALFM_C114410WA	orf19	5.26	7.13	-0.439	0.122
DNA-directed RNA polymerase I subunit	Q5AP67	CAALFM_C110260CA	RPA34	1.07	1.45	-0.438	0.123
Proteasome regulatory particle base subunit	Q5A2A0	CAALFM_C105240CA	RPT1	1.52	2.05	-0.432	0.124
Tyrosine--tRNA ligase	Q5AFB3	CAALFM_C402980WA	TYS1	5.34	7.19	-0.429	0.125
Pho88p	A0A1D8PTX9	CAALFM_CR09320CA	PHO88	1.52	2.04	-0.425	0.126
Superoxide dismutase	A0A1D8PQH5	CAALFM_C700110WA	SOD3	29.63	39.67	-0.421	0.126
Plasma membrane ATPase	A0A1D8PJ01	CAALFM_C300720WA	PMA1	60.68	81.01	-0.417	0.127
Serine--tRNA ligase, cytoplasmic	Q9HGT6	CAALFM_C302780WA	SES1	9.98	13.32	-0.417	0.127
Dihydroorotase	Q5A1L8	CAALFM_C500900CA	URA4	28.59	38.15	-0.416	0.128
Cytochrome c oxidase subunit 9, mitochondrial	A0A1D8PHI5	CAALFM_C205930WA	COX9	1.53	2.04	-0.415	0.128
Sugar phosphate phosphatase	A0A1D8PLC6	CAALFM_C401690CA	HRT2	1.53	2.04	-0.415	0.128
Uncharacterized protein	A0A1D8PQ22	CAALFM_C602930WA	orf19	1.53	2.04	-0.415	0.128
Lap4p	A0A1D8PNQ9	CAALFM_C503590WA	LAP4	7.12	9.49	-0.415	0.128
ATP phosphoribosyltransferase	P46586	CAALFM_C505320CA	HIS1	6.5	8.64	-0.411	0.129
rRNA-binding ribosome biosynthesis protein	A0A1D8PU19	CAALFM_CR09730CA	SSF1	1.08	1.43	-0.405	0.130
Major glycerophosphoinositol permease GIT3	Q5A1L6	CAALFM_C500880CA	GIT3	1.08	1.43	-0.405	0.130
DNA-directed RNA polymerase subunit	A0A1D8PHV8	CAALFM_C207300CA	RPA12	2.57	3.39	-0.400	0.131
Acetyltransferase component of pyruvate dehydrogenase complex	Q5AGX8	CAALFM_C701640WA	LAT1	21.63	28.5	-0.398	0.132
Tom22p	Q59LZ9	CAALFM_C702680WA	TOM22	5.39	7.1	-0.398	0.132
Ribosomal 60S subunit protein L10	Q5AIB8	CAALFM_C102460WA	RPL10	19.54	25.67	-0.394	0.133
DNA-directed RNA polymerases I, II, and III subunit RPABC3	Q59MZ8	CAALFM_CR04780WA	RPB8	2.57	3.37	-0.391	0.133
5-methyltetrahydropteroyltriglutamate--homocysteine methyltransferase	P82610	CAALFM_CR01620CA	MET6	264.91	346.76	-0.388	0.134
Uncharacterized protein	Q5AHI3	CAALFM_C204080WA	orf19	8.19	10.7	-0.386	0.134
Fox3p	Q5AJ90	CAALFM_C301460CA	FOX3	5.96	7.78	-0.385	0.135
F1F0 ATP synthase subunit i	A0A1D8PG50	CAALFM_C200610CA	ATP18	1.61	2.1	-0.383	0.135
Alkaline phosphatase	Q5APN0	CAALFM_C108780WA	orf19	5.62	7.33	-0.383	0.135
Homocitrate synthase	Q59VG1	CAALFM_C104730CA	LYS21	3.16	4.1	-0.376	0.137
Ran GTPase-binding protein	A0A1D8PES3	CAALFM_C110100CA	NTF2	31.42	40.69	-0.373	0.137
Ribosomal 60S subunit protein L30	A0A1D8PM75	CAALFM_C404900WA	RPL30	6.77	8.75	-0.370	0.138
Alpha-ketoglutarate dehydrogenase	A0A1D8PTH3	CAALFM_CR07420WA	KGD2	13.78	17.81	-0.370	0.138

Transcription regulator	Q5ADP3	CAALFM_C307020WA	SSN6	1.56	2.01	-0.366	0.139
J domain-containing protein	Q5A441	CAALFM_C405810WA	orf19	4.13	5.31	-0.363	0.140
Yhm1p	A0A1D8PPR4	CAALFM_C601930WA	YHM1	5.16	6.61	-0.357	0.141
ATP-dependent RNA helicase	A0A1D8PKZ3	CAALFM_C400220CA	SUB2	5.27	6.74	-0.355	0.141
Uncharacterized protein	Q5AHJ1	CAALFM_C204160WA	orf19	1.6	2.04	-0.351	0.142
Actin-related protein 2/3 complex subunit	A0A1D8PT60	CAALFM_CR06180WA	ARC40	4.2	5.35	-0.349	0.143
CoA_binding domain-containing protein	Q5A3M0	CAALFM_C112140WA	orf19	11.29	14.26	-0.337	0.145
Ribokinase	A0A1D8PFI3	CAALFM_C112780WA	RBK1	3.74	4.71	-0.333	0.146
Ribosomal 60S subunit protein L16A	Q5AB87	CAALFM_C100180WA	RPL16A	9.28	11.67	-0.331	0.147
Ribosomal 60S subunit protein L7A	A0A1D8PDL6	CAALFM_C105720WA	orf19	15.97	19.97	-0.323	0.149
13 kDa ribonucleoprotein-associated protein	Q5ANL6	CAALFM_C304380CA	SNU13	3.66	4.57	-0.320	0.149
Flavodoxin-like fold family protein	A0A1D8PT02	CAALFM_CR05390WA	PST3	28.57	35.62	-0.318	0.149
Ras-related protein SEC4	P0CY31	CAALFM_CR01750CA	SEC4	6.14	7.65	-0.317	0.150
Coronin	A0A1D8PQZ0	CAALFM_C701850CA	CRN1	4.09	5.09	-0.316	0.150
Very-long-chain 3-oxoacyl-CoA reductase	Q59V93	CAALFM_CR06070WA	CAALFM_CR06070WA	2.05	2.55	-0.315	0.150
Fre30p	A0A1D8PTF8	CAALFM_CR07280WA	FRE30	1.53	1.9	-0.313	0.151
Survival factor 1	Q5ABA2	CAALFM_C100400WA	SVF1	1.53	1.9	-0.313	0.151
Uncharacterized protein	A0A1D8PQ54	CAALFM_C603320WA	orf19	13.69	16.98	-0.311	0.151
S-(hydroxymethyl)glutathione dehydrogenase	A0A1D8PU61	CAALFM_CR10250CA	FDH3	5.34	6.61	-0.308	0.152
60S ribosomal protein L20	A0A1D8PLC9	CAALFM_C401520CA	RPL20B	11.88	14.69	-0.306	0.152
Uncharacterized protein	A0A1D8PQM5	CAALFM_C700520WA	orf19	1.57	1.94	-0.305	0.152
Aminopeptidase 2	Q59KZ1	CAALFM_C104400CA	APE2	24.71	30.45	-0.301	0.153
Uncharacterized protein	A0A1D8PF49	CAALFM_C111320CA	orf19	18.2	22.4	-0.300	0.154
Coatomer subunit delta	A0A1D8PND9	CAALFM_C502300CA	RET2	2.1	2.58	-0.297	0.154
Aminomethyltransferase	A0A1D8PPW9	CAALFM_C602500CA	GCV1	8.28	10.17	-0.297	0.154
Ribosomal 60S subunit protein L32	A0A1D8PPN6	CAALFM_C601700WA	RPL32	16.21	19.88	-0.294	0.155
17-beta-hydroxysteroid dehydrogenase-like protein	A0A1D8PFV8	CAALFM_C114060WA	orf19	6.83	8.37	-0.293	0.155
Cytochrome b-c1 complex subunit Rieske, mitochondrial	A0A1D8PJX3	CAALFM_C304430WA	RIP1	6.72	8.23	-0.292	0.155
ATP-dependent RNA helicase DBP5	Q5AJD0	CAALFM_C301860CA	DBP5	2.11	2.58	-0.290	0.156
Triosephosphate isomerase	Q9P940	CAALFM_C307440WA	TPI1	86.52	105.55	-0.287	0.157
Uncharacterized protein	Q59N01	CAALFM_CR04820WA	CAALFM_CR04820WA	4.6	5.61	-0.286	0.157
NADH-cytochrome b5 reductase 2	Q59M70	CAALFM_C602040WA	MCR1	26.99	32.91	-0.286	0.157
Uncharacterized protein	A0A1D8PKD0	CAALFM_C305880CA	orf19	2.1	2.56	-0.286	0.157
ATP synthase subunit e, mitochondrial	A0A1D8PL02	CAALFM_C400330CA	orf19	2.1	2.56	-0.286	0.157
Glutathione peroxidase	Q59WW7	CAALFM_C600850WA	orf19	8.04	9.79	-0.284	0.157
Uncharacterized protein	A0A1D8PRA6	CAALFM_C703270WA	orf19	7.75	9.43	-0.283	0.157
60S ribosomal protein L6	A0A1D8PCX8	CAALFM_C103110WA	RPL6	16.3	19.76	-0.278	0.159
Ribosomal 40S subunit protein S11A	A0A1D8PN83	CAALFM_C501540WA	orf19	8.21	9.95	-0.277	0.159
Ribosomal 60S subunit protein L14B	A0A1D8PFL9	CAALFM_C113050WA	RPL14	12.38	14.96	-0.273	0.160
Succinate dehydrogenase [ubiquinone] flavoprotein subunit, mitochondrial	Q5A2A1	CAALFM_C105260CA	SDH1	5.12	6.15	-0.264	0.162
40S ribosomal protein S22-B	P0CU35	CAALFM_C103620CA	RPS22B	6.72	8.05	-0.261	0.162
Ribosomal 60S subunit protein L4B	A0A1D8PFV1	CAALFM_C114110CA	RPL4B	37.13	44.44	-0.259	0.163
Homoserine dehydrogenase	Q5AIA2	CAALFM_C102620CA	HOM6	16.63	19.89	-0.258	0.163
Proteasome core particle subunit beta 6	A0A1D8PLK1	CAALFM_C402470CA	orf19	5.13	6.13	-0.257	0.163
Aldo_ket_red domain-containing protein	A0A1D8PD74	CAALFM_C104010CA	orf19	2.56	3.05	-0.253	0.164
Lysine--tRNA ligase	Q5ADU2	CAALFM_C307410CA	KRS1	9.23	10.99	-0.252	0.164
Transcription initiation factor IIA subunit 2	Q5AMM1	CAALFM_C401680WA	TOA2	4.3	5.12	-0.252	0.164
Fumarate reductase	Q5AKX2	CAALFM_C113670WA	OSM2	19.33	22.99	-0.250	0.165
60S acidic ribosomal protein P0	A0A1D8PQS0	CAALFM_C700990WA	RPP0	16.8	19.91	-0.245	0.166
Ubiquinol--cytochrome-c reductase subunit	A0A1D8PP59	CAALFM_C505230CA	orf19	21.13	25.04	-0.245	0.166
H/ACA ribonucleoprotein complex subunit	A0A1D8PF56	CAALFM_C111550WA	GAR1	2.16	2.55	-0.240	0.167
Glutamate--tRNA ligase	A0A1D8PQL8	CAALFM_C700620WA	GUS1	6.94	8.17	-0.235	0.168
60S ribosomal subunit assembly/export protein LOC1	Q5AJF1	CAALFM_C302040CA	LOC1	2.15	2.53	-0.235	0.168
E2 ubiquitin-conjugating protein	Q59T91	CAALFM_C101830CA	UBC8	3.23	3.8	-0.235	0.168
Peptidase_M24 domain-containing protein	A0A1D8PR11	CAALFM_C702100WA	orf19	5.79	6.81	-0.234	0.168
Uncharacterized protein	A0A1D8PD11	CAALFM_C103510CA	orf19	14.28	16.79	-0.234	0.168
Peroxiredoxin TSA1-B	P0CU34	CAALFM_C306180CA	TSA1B	32.27	37.88	-0.231	0.169
Uncharacterized protein	Q59US8	CAALFM_C400170WA	orf19	11.28	13.24	-0.231	0.169
MICOS complex subunit MIC60	Q5A044	CAALFM_CR03530WA	MIC60	6.14	7.2	-0.230	0.169
Uncharacterized protein	A0A1D8PCP4	CAALFM_C102270CA	orf19	3.07	3.6	-0.230	0.169
Uncharacterized protein	A0A1D8PTY0	CAALFM_CR09240CA	CAALFM_CR09240CA	3.25	3.8	-0.226	0.170
Uncharacterized protein	A0A1D8PHQ3	CAALFM_C206630CA	orf19	2.61	3.05	-0.225	0.170
Tim12p	Q5AMM6	CAALFM_C401740WA	TIM12	4.16	4.86	-0.224	0.171
Cyb5p	A0A1D8PQP7	CAALFM_C700700WA	CYB5	4.6	5.35	-0.218	0.172
Septin	A0A1D8PD83	CAALFM_C104210CA	CDC3	3.09	3.58	-0.212	0.173
Ribosomal 40S subunit protein S3	A0A1D8PSV5	CAALFM_CR04810WA	RPS3	21.6	24.92	-0.206	0.175
Translation initiation factor eIF2 subunit beta	A0A1D8PRJ6	CAALFM_C704130CA	SUI3	5.34	6.15	-0.204	0.175
Endoplasmic reticulum transmembrane protein	A0A1D8PGL1	CAALFM_C202410WA	orf19	2.15	2.46	-0.194	0.177
Ribosomal 40S subunit protein S14B	A0A1D8PDT3	CAALFM_C106450CA	RPS14B	13.41	15.34	-0.194	0.177
Lactoylglutathione lyase	Q5ABB2	CAALFM_C100500CA	GLO1	23.43	26.76	-0.192	0.178
Ifr2p	A0A1D8PSE7	CAALFM_CR03280WA	IFR2	3.59	4.1	-0.192	0.178
Coatomer subunit epsilon	A0A1D8PMT6	CAALFM_C500080CA	orf19	4.16	4.75	-0.191	0.178
Uncharacterized protein	Q59TB4	CAALFM_C204370WA	orf19	2.06	2.35	-0.190	0.178
ATP-binding cassette family ATPase	Q5A2T2	CAALFM_C208000CA	KRE30	2.56	2.92	-0.190	0.178
LsmAD domain-containing protein	Q5A7K7	CAALFM_C300130CA	orf19	3.16	3.6	-0.188	0.179
Bifunctional AP-4-A phosphorylase/ADP sulfurylase	A0A1D8PQ57	CAALFM_C603410CA	APA2	2.69	3.05	-0.181	0.180
Acyl carrier protein	Q5AHH7	CAALFM_C204030CA	ACP12	10.19	11.52	-0.177	0.181
Proteasome endopeptidase complex	A0A1D8PRH6	CAALFM_C704020CA	PRE5	8.19	9.25	-0.176	0.181
Hexokinase-2	P83776	CAALFM_CR04510WA	HXK2	41.02	45.93	-0.163	0.184
Ribosomal 60S subunit protein L9B	Q5AEN2	CAALFM_C302470CA	RPL9B	17.85	19.97	-0.162	0.185
Lhp1p	A0A1D8PE36	CAALFM_C107500CA	LHP1	10	11.18	-0.161	0.185
Pyridoxal phosphate homeostasis protein	A0A1D8PE30	CAALFM_C107510WA	orf19	2.63	2.94	-0.161	0.185
Dld1p	A0A1D8PGR9	CAALFM_C202980CA	DLD1	5.62	6.28	-0.160	0.185
Malate dehydrogenase, cytoplasmic	P83778	CAALFM_CR00540CA	MDH1	31.45	35.12	-0.159	0.185
ADP-ribosylation factor GTPase-activating protein	Q5A7M1	CAALFM_C300240CA	GLO3	4.61	5.11	-0.149	0.188
Protein SDS23	Q5A744	CAALFM_C108370WA	SDS24	4.61	5.09	-0.143	0.189
Arf family GTPase	Q5AND9	CAALFM_C304950WA	ARF2	4.86	5.35	-0.139	0.190
Metalloaminopeptidase	A0A1D8PJE0	CAALFM_C302170CA	LAP41	38.71	42.57	-0.137	0.190
Cell wall protein RHD3	Q5A5U4	CAALFM_C404050CA	RHD3	3.25	3.57	-0.136	0.190
Mitochondrial 37S ribosomal protein MRP1	A0A1D8PJB1	CAALFM_C301850WA	orf19	5.12	5.62	-0.134	0.191
Uncharacterized protein	Q5ANH8	CAALFM_C304650WA	orf19	2.61	2.85	-0.127	0.192
Uncharacterized protein	A0A1D8PNE5	CAALFM_C502380WA	orf19	9.98	10.78	-0.111	0.196
F1F0 ATP synthase subunit 4	Q59ZE0	CAALFM_C205500WA	ATP4	14.85	16.04	-0.111	0.196
Proteinase B	A0A1D8PRH0	CAALFM_C703860WA	orf19	7.52	8.12	-0.111	0.196
RRM domain-containing protein	A0A1D8PIF0	CAALFM_C209650WA	orf19	9.47	10.22	-0.110	0.196

Chitin biosynthesis protein CHS5	O74161	CAALFM_C204140WA	CHS5	4.11	4.41	-0.102	0.198
Profilin	Q5A786	CAALFM_C108030WA	PFY1	13.28	14.23	-0.100	0.198
Cytochrome b-c1 complex subunit 7	Q5ABS1	CAALFM_C603400CA	QCR7	10.51	11.18	-0.089	0.201
Inhibitor I9 domain-containing protein	Q5AF37	CAALFM_C402340WA	orf19	21.04	22.38	-0.089	0.201
Rct1p	A0A1D8PKD1	CAALFM_C305710WA	RCT1	74.63	79.19	-0.086	0.202
3'(2'),5'-bisphosphate nucleotidase	P0CY20	CAALFM_C600970CA	HAL21	4.1	4.34	-0.082	0.202
Reticulon-like protein	A0A1D8PM53	CAALFM_C404800WA	orf19	13.3	14.06	-0.080	0.203
Coproporphyrinogen oxidase	Q59MR4	CAALFM_C304060CA	HEM13	5.78	6.11	-0.080	0.203
Mrf1p	Q59TU5	CAALFM_C111700CA	MRF1	10.73	11.2	-0.062	0.207
Peptidyl-prolyl cis-trans isomerase	A0A1D8PKL0	CAALFM_C306360CA	CYP5	14.48	15.1	-0.061	0.207
Uncharacterized protein	A0A1D8PIS1	CAALFM_C210650WA	orf19	5.37	5.59	-0.058	0.208
FG-nucleoporin	A0A1D8PKV6	CAALFM_C307780CA	NUP159	0.5	0.52	-0.057	0.208
Karyopherin beta	Q59VX7	CAALFM_C102240WA	orf19	0.5	0.52	-0.057	0.208
Uncharacterized protein	A0A1D8PGP5	CAALFM_C202620WA	orf19	3.68	3.82	-0.054	0.209
Lipid-binding protein	Q59KV8	CAALFM_C206730WA	LSP1	34.37	35.66	-0.053	0.209
Cytochrome b5 heme-binding domain-containing protein	A0A1D8PHE5	CAALFM_C205550WA	orf19	5.16	5.35	-0.052	0.209
Uncharacterized protein	Q5ANB5	CAALFM_C305150WA	orf19	15.83	16.4	-0.051	0.209
V-type proton ATPase subunit D	P87220	CAALFM_C406400CA	VMA8	3.69	3.82	-0.050	0.210
L51_S25_CI-B8 domain-containing protein	A0A1D8PQU3	CAALFM_C701160CA	orf19	6.44	6.65	-0.046	0.210
Heat shock protein 90 homolog	P46598	CAALFM_C702030WA	HSP90	42.23	43.54	-0.044	0.211
MICOS complex subunit	A0A1D8PF88	CAALFM_C111760CA	orf19	1.02	1.05	-0.042	0.211
DNA-directed RNA polymerase core subunit	A0A1D8PFX2	CAALFM_C114390WA	RPC10	1.02	1.05	-0.042	0.211
Ist2p	A0A1D8PE41	CAALFM_C107520CA	IST2	7.37	7.55	-0.035	0.213
Uncharacterized protein	Q5A8Z9	CAALFM_C101490WA	orf19	22.72	23.26	-0.034	0.213
Superoxide dismutase [Cu-Zn]	A0A1D8PLJ3	CAALFM_C402320CA	SOD1	29.94	30.63	-0.033	0.213
Bifunctional 4-hydroxy-4-methyl-2-oxoglutarate aldolase/oxaloacetate decarboxylase	Q5AP69	CAALFM_C110240CA	orf19	5.13	5.24	-0.031	0.214
F1F0 ATP synthase subunit h	A0A1D8PHL7	CAALFM_C206290CA	ATP14	9.98	10.18	-0.029	0.214
Flavodoxin-like fold family protein	A0A1D8PT03	CAALFM_CR05380CA	YCP4	8.22	8.38	-0.028	0.215
ANK_REP_REGION domain-containing protein	Q5ANE2	CAALFM_C304920CA	orf19	4.63	4.72	-0.028	0.215
Uncharacterized protein	A0A1D8PSE0	CAALFM_CR03120WA	CAALFM_CR03120WA	14.47	14.75	-0.028	0.215
Eukaryotic translation initiation factor 4E	Q9P975	CAALFM_CR10490WA	TIF45	7	7.13	-0.027	0.215
Copper metallochaperone	A0A1D8PTB8	CAALFM_CR06950CA	ATX1	8.98	9.12	-0.022	0.216
Putative NADPH-dependent methylglyoxal reductase GRP2	P83775	CAALFM_C502860CA	GRP2	11.57	11.73	-0.020	0.216
Adenylate kinase	Q5A4Q1	CAALFM_C601910WA	ADK1	32.2	32.61	-0.018	0.217
Phm7p	A0A1D8PI19	CAALFM_C208140CA	PHM7	5.63	5.7	-0.018	0.217
Elongation factor 3	P25997	CAALFM_C501580CA	CEF3	67.85	68.22	-0.008	0.219
Ribosomal 40S subunit protein S10A	A0A1D8PI15	CAALFM_C208040CA	RPS10	6.14	6.15	-0.002	0.220
GTPase-activating protein	A0A1D8PJD7	CAALFM_C301990WA	RNA1	6.27	6.28	-0.002	0.220
Phospho-2-dehydro-3-deoxyheptonate aldolase, tyrosine-inhibited	P79023	CAALFM_C105110CA	ARO4	8.22	8.23	-0.002	0.220
ENTH domain-containing protein	A0A1D8PL36	CAALFM_C400590CA	orf19	0.5	0.5	0.000	0.221
Mitochondrial 54S ribosomal protein RML2	Q5A7P6	CAALFM_C300450CA	orf19	0.5	0.5	0.000	0.221
Glucosamine 6-phosphate N-acetyltransferase	Q5AHF9	CAALFM_C203870WA	GNA1	0.5	0.5	0.000	0.221
Oxysterol-binding protein-like protein OBPa	Q9UW25	CAALFM_C501770CA	OBPA	0.5	0.5	0.000	0.221
Muc1p	A0A1D8PL32	CAALFM_C400600CA	MUC1	1.02	1.02	0.000	0.221
Axl2p	A0A1D8PM01	CAALFM_C404170CA	AXL2	1.02	1.02	0.000	0.221
Sgd1p	A0A1D8PSJ3	CAALFM_CR03650WA	SGD1	1.02	1.02	0.000	0.221
Nucleoporin	A0A1D8PD79	CAALFM_C104200CA	orf19	1.05	1.05	0.000	0.221
Septation protein 7	Q59VX8	CAALFM_C102230WA	9/7/2021	1.05	1.05	0.000	0.221
U3 small nucleolar RNA-associated protein 11	Q59UH8	CAALFM_CR03360WA	CAALFM_CR03360WA	0.52	0.52	0.000	0.221
MFS domain-containing protein	Q5AGW0	CAALFM_C701510WA	orf19	2.05	2.05	0.000	0.221
Repressed by TUP1 protein 5	Q59UT4	CAALFM_C400130WA	RBT5	8.16	8.15	0.002	0.221
Mitochondrial zinc maintenance protein 1, mitochondrial	Q5A7N3	CAALFM_C300340WA	MZM1	7.69	7.67	0.004	0.222
60S ribosomal protein L8	Q5ANA1	CAALFM_C305240CA	RPL8B	47.8	47.65	0.005	0.222
Phosphorelay intermediate protein YPD1	Q59WC6	CAALFM_C107240WA	YPD1	9.19	9.16	0.005	0.222
Actin-related protein 2/3 complex subunit 5	Q59RN2	CAALFM_CR07210WA	ARC15	11.25	11.2	0.006	0.222
Ubiquinol--cytochrome-c reductase subunit 6	A0A1D8PJT8	CAALFM_C304080WA	orf19	2.05	2.04	0.007	0.222
Mitochondrial 54S ribosomal protein YmL32	Q5A6K5	CAALFM_CR04580WA	CAALFM_CR04580WA	2.05	2.04	0.007	0.222
Uncharacterized protein	Q5A785	CAALFM_C108050WA	orf19	1.55	1.54	0.009	0.223
Aldehyde dehydrogenase	A0A1D8PC76	CAALFM_C100410CA	orf19	1.53	1.52	0.009	0.223
Phosphatidylglycerol/phosphatidylinositol transfer protein	Q5A8A2	CAALFM_CR01280CA	NPC2	4.79	4.75	0.012	0.224
40S ribosomal protein S1	P40910	CAALFM_C103090WA	RPS1	22.19	22	0.012	0.224
Ribosomal 40S subunit protein S18B	A0A1D8PQQ5	CAALFM_C700960WA	RPS18	24.73	24.51	0.013	0.224
Lon protease homolog, mitochondrial	Q5A6N1	CAALFM_CR04340WA	PIM1	2.12	2.1	0.014	0.224
Calmodulin-dependent protein kinase	Q59XV1	CAALFM_C210260CA	CMK2	2.06	2.04	0.014	0.224
NFACT-R_1 domain-containing protein	A0A1D8PQD9	CAALFM_C604260CA	orf19	1.03	1.02	0.014	0.224
Uncharacterized protein	A0A1D8PQI8	CAALFM_C700240WA	orf19	1.03	1.02	0.014	0.224
Mso1p	Q5A3M2	CAALFM_C112160WA	MSO1	1.03	1.02	0.014	0.224
Uncharacterized protein	A0A1D8PQJ8	CAALFM_C700350CA	orf19	4.84	4.79	0.015	0.224
Ribosomal 60S subunit protein L23B	A0A1D8PPT5	CAALFM_C602070CA	RPL23A	12.88	12.74	0.016	0.224
Bbc1p	A0A1D8PE54	CAALFM_C107540CA	BBC1	8.75	8.64	0.018	0.225
Uncharacterized protein	A0A1D8PT84	CAALFM_CR06530WA	CAALFM_CR06530WA	3.09	3.05	0.019	0.225
60S ribosomal protein L8	A0A1D8PF11	CAALFM_C111030WA	RPL82	47.3	46.6	0.022	0.226
Nucleoside diphosphate kinase	Q5AG68	CAALFM_C502890WA	YNK1	29.05	28.53	0.026	0.227
Uncharacterized protein	A0A1D8PPX6	CAALFM_C602560WA	orf19	17.15	16.84	0.026	0.227
Ubiquitin carboxyl-terminal hydrolase	A0A1D8PGI9	CAALFM_C202020WA	orf19	1.07	1.05	0.027	0.227
Methionine aminopeptidase 2	Q59LF9	CAALFM_C604080WA	MAP2	14.48	14.21	0.027	0.227
Protein YAE1	Q9P840	CAALFM_C700800CA	YAE1	1.07	1.05	0.027	0.227
Stf2p	A0A1D8PG09	CAALFM_C200250WA	STF2	19.7	19.3	0.030	0.227
Mlc1p	A0A1D8PSE1	CAALFM_CR03090CA	MLC1	15.47	15.1	0.035	0.229
Ecm15p	A0A1D8PKH0	CAALFM_C306480CA	ECM15	1.08	1.05	0.041	0.230
Srp40p	A0A1D8PSB9	CAALFM_CR02980CA	SRP40	1.08	1.05	0.041	0.230
Calcineurin regulatory subunit B	A0A1D8PP58	CAALFM_C505160CA	CNB1	2.1	2.04	0.042	0.230
Uncharacterized protein	A0A1D8PRQ4	CAALFM_CR00430CA	CAALFM_CR00430CA	2.1	2.04	0.042	0.230
Cell division control protein 42 homolog	P0CY33	CAALFM_C108450CA	CDC42	2.1	2.04	0.042	0.230
DNA-directed RNA polymerase subunit	Q59Z24	CAALFM_C207200WA	orf19	2.1	2.04	0.042	0.230
Endoplasmic reticulum vesicle protein 25	Q5A302	CAALFM_C306250WA	ERV25	2.1	2.04	0.042	0.230
Uncharacterized protein	Q5APD5	CAALFM_C109670CA	orf19	2.1	2.04	0.042	0.230
Smi1p	A0A1D8PE53	CAALFM_C107870CA	SMI1	1.05	1.02	0.042	0.230
Uncharacterized protein	A0A1D8PM60	CAALFM_C404870CA	orf19	1.05	1.02	0.042	0.230
YL1_C domain-containing protein	A0A1D8PML5	CAALFM_C406450WA	orf19	1.05	1.02	0.042	0.230
Mitochondrial 54S ribosomal protein YmL49	A0A1D8PRA1	CAALFM_C703000CA	orf19	1.05	1.02	0.042	0.230
Uncharacterized protein	Q5A2K4	CAALFM_C207800WA	orf19	1.05	1.02	0.042	0.230
Uncharacterized protein	Q5A431	CAALFM_C405890WA	orf19	1.05	1.02	0.042	0.230
Protein ROT1	Q5ABP8	CAALFM_C100770CA	ROT1	1.05	1.02	0.042	0.230
DUF4149 domain-containing protein	A0A1D8PFM0	CAALFM_C113190WA	orf19	1.05	1.02	0.042	0.230

Spermidine synthase	Q59Z50	CAALFM_C206960WA	SPE3	1.05	1.02	0.042	0.230
Cytochrome c	P53698	CAALFM_C210110WA	CYC1	9.43	9.16	0.042	0.230
Uncharacterized protein	A0A1D8PU51	CAALFM_CR10140WA	CAALFM_CR10140WA	9.71	9.42	0.044	0.231
Rho family GTPase	A0A1D8PDV5	CAALFM_C106730WA	RAC1	2.63	2.55	0.045	0.231
CAP-Gly domain-containing protein	A0A1D8PKW0	CAALFM_C307770CA	orf19	4.21	4.07	0.049	0.232
Actin cytoskeleton-regulatory complex protein SLA1	Q5ALV2	CAALFM_C201640WA	SLA1	1.08	1.04	0.054	0.233
Uncharacterized protein	A0A1D8PPM1	CAALFM_C601460CA	orf19	1.6	1.54	0.055	0.233
Polyubiquitin-binding protein	Q5A0H8	CAALFM_C202730WA	orf19	1.02	0.98	0.058	0.234
Coi1p	Q5A799	CAALFM_C107900WA	COI1	2.15	2.06	0.062	0.235
Respiratory supercomplex factor 1, mitochondrial	Q59N74	CAALFM_C503800WA	RCF1	3.19	3.05	0.065	0.235
Uncharacterized protein	A0A1D8PH05	CAALFM_C203930CA	SNF6	2.16	2.06	0.068	0.236
Uncharacterized protein	A0A1D8PSW5	CAALFM_CR04940WA	CAALFM_CR04940WA	1.07	1.02	0.069	0.236
Mitochondrial import inner membrane translocase subunit TIM8	Q59MI8	CAALFM_C307890WA	TIM8	1.07	1.02	0.069	0.236
Type 1 phosphatases regulator YPI1	Q59ZW4	CAALFM_C114190CA	YPI1	1.07	1.02	0.069	0.236
Putative lipase ATG15	Q5A4N0	CAALFM_C601740CA	ATG15	1.07	1.02	0.069	0.236
Iron-sulfur cluster assembly protein	Q5AGZ0	CAALFM_C701760CA	ISU1	11.76	11.2	0.070	0.237
Uncharacterized protein	Q5AAR2	CAALFM_C106600WA	orf19	12.44	11.84	0.071	0.237
Pyrroline-5-carboxylate reductase	A0A1D8PKZ7	CAALFM_C400240CA	PRO3	3.07	2.92	0.072	0.237
Ribosomal 40S subunit protein S23B	A0A1D8PDU3	CAALFM_C106580WA	RPS23A	13.5	12.84	0.072	0.237
Rga2p	Q5AMQ3	CAALFM_C402000CA	RGA2	2.66	2.53	0.072	0.237
mRNA-binding translational activator	Q59YJ9	CAALFM_C501910WA	GIS2	22.1	21	0.074	0.237
snoRNP complex protein	A0A1D8PU46	CAALFM_CR09950CA	SIK1	11.81	11.21	0.075	0.238
Ubiquitin carboxyl-terminal hydrolase	A0A1D8PC77	CAALFM_C100440WA	UBP6	2.15	2.04	0.076	0.238
NUC153 domain-containing protein	A0A1D8PEZ5	CAALFM_C110970WA	orf19	2.15	2.04	0.076	0.238
Adenylyl-sulfate kinase	Q5A500	CAALFM_C500430WA	MET14	2.15	2.04	0.076	0.238
Histone	A0A1D8PR93	CAALFM_C703180CA	HHO1	9.74	9.22	0.079	0.239
Pyridoxine biosynthesis protein	Q5AIA6	CAALFM_C102590CA	SNZ1	41.56	39.33	0.080	0.239
RXT2_N domain-containing protein	Q5AMN1	CAALFM_C401780CA	orf19	2.05	1.94	0.080	0.239
Sulfhydryl oxidase	A0A1D8PSD6	CAALFM_CR03010CA	ERV1	7.58	7.17	0.080	0.239
Type I HSP40 co-chaperone	A0A1D8PSQ3	CAALFM_CR04200WA	YDJ1	20.07	18.97	0.081	0.239
Malate dehydrogenase	Q5A5S6	CAALFM_C210480WA	MDH1-3	5.62	5.31	0.082	0.239
Fmp45p	A0A1D8PR25	CAALFM_C702240WA	FMP45	1.63	1.54	0.082	0.239
Nucleolar protein 58	Q59S06	CAALFM_C600370CA	NOP58	20	18.89	0.082	0.239
Uncharacterized protein	A0A1D8PU60	CAALFM_CR10180WA	CAALFM_CR10180WA	2.16	2.04	0.083	0.239
Uncharacterized protein	A0A1D8PLG7	CAALFM_C402200CA	orf19	1.08	1.02	0.083	0.239
Uncharacterized protein	Q59ZZ8	CAALFM_CR03470WA	CAALFM_CR03470WA	1.08	1.02	0.083	0.239
Septin	A0A1D8PCY5	CAALFM_C103210CA	CDC12	1.08	1.02	0.083	0.239
Amino acid transporter	Q59RL1	CAALFM_C600330CA	GNP1	1.08	1.02	0.083	0.239
COX assembly mitochondrial protein	Q5ALU3	CAALFM_C201720CA	orf19	1.08	1.02	0.083	0.239
Mitochondrial intermembrane space import and assembly protein 40	O94030	CAALFM_C102880CA	MIA40	18.38	17.33	0.085	0.240
Uncharacterized protein	Q5ABD5	CAALFM_C100700WA	orf19	1.52	1.43	0.088	0.241
snoRNP complex protein	A0A1D8PTN4	CAALFM_CR08020CA	NOP10	3.25	3.05	0.092	0.241
Type 1 phosphatases regulator YPI2	Q59ZU1	CAALFM_C114350WA	YPI2	8.19	7.67	0.095	0.242
Acyl carrier protein	A0A1D8PDT0	CAALFM_C106060CA	ACP1	4.35	4.07	0.096	0.242
37S ribosomal protein S24, mitochondrial	Q5AI30	CAALFM_C103280WA	orf19	2.63	2.46	0.096	0.242
Transcription initiation factor IIA large subunit	Q5AFC5	CAALFM_C403090WA	orf19	15.25	14.2	0.103	0.244
Uncharacterized protein	A0A1D8PGJ9	CAALFM_C202310WA	orf19	3.62	3.37	0.103	0.244
Cytochrome c peroxidase, mitochondrial	Q5AEN1	CAALFM_C302480CA	CCP1	24.07	22.36	0.106	0.245
Mam33p	A0A1D8PRG4	CAALFM_C703930CA	MAM33	24.21	22.43	0.110	0.246
RRM domain-containing protein	A0A1D8PQJ1	CAALFM_C700250CA	orf19	11.32	10.48	0.111	0.246
Palmitoyltransferase	Q5AI79	CAALFM_C102860CA	YKT6	3.16	2.92	0.114	0.246
Uncharacterized protein	A0A1D8PN37	CAALFM_C501190WA	orf19	1.05	0.97	0.114	0.246
Ribosomal 60S subunit protein L42A	A0A1D8PEV4	CAALFM_C110390CA	RPL42	1.05	0.97	0.114	0.246
Proteasome regulatory particle lid subunit	Q59SD0	CAALFM_C208930WA	orf19	2.1	1.94	0.114	0.246
Peptide-methionine	Q5A942	CAALFM_C101040WA	orf19	2.1	1.94	0.114	0.246
Ribosomal protein L37	A0A1D8PF45	CAALFM_C111360WA	RPL37B	17.88	16.49	0.117	0.247
Uncharacterized protein	Q5AB84	CAALFM_C100160CA	orf19	41.41	38.17	0.118	0.247
Ras-like protein 1	Q59XU5	CAALFM_C210210CA	RAS1	13.35	12.3	0.118	0.247
Deoxyuridine 5'-triphosphate nucleotidohydrolase	P0CY19	CAALFM_C101330CA	DUT1	9.48	8.73	0.119	0.247
Arc1p	A0A1D8PSC8	CAALFM_CR03060WA	ARC1	15.87	14.61	0.119	0.248
Rdi1p	Q5AND4	CAALFM_C305000WA	RDI1	12.1	11.14	0.119	0.248
Trifunctional formate-tetrahydrofolate ligase/methenyltetrahydrofolate cyclohydrolase/methylenetetrahydrofo	Q59SM8	CAALFM_CR07010WA	MIS11	16.83	15.48	0.121	0.248
Ribosomal 40S subunit protein S20	Q5A389	CAALFM_CR08150WA	RPS20	14.94	13.74	0.121	0.248
SCF ubiquitin ligase subunit	Q59WE2	CAALFM_C107410CA	SKP1	15.15	13.92	0.122	0.248
L-methionine (R)-S-oxide reductase	A0A1D8PPZ9	CAALFM_C602800WA	orf19	4.73	4.34	0.124	0.249
mRNA-binding protein	A0A1D8PFV4	CAALFM_C114280CA	NPL3	9.43	8.64	0.126	0.249
Edc3p	A0A1D8PM93	CAALFM_C405190CA	EDC3	1.55	1.42	0.126	0.249
Adenosine kinase	A0A1D8PQ26	CAALFM_C603080CA	ADO1	54.2	49.62	0.127	0.249
Translation termination factor GTPase eRF3	Q59YE8	CAALFM_C209720WA	SUP35	6.16	5.61	0.135	0.251
F1F0 ATP synthase subunit delta	A0A1D8PUD2	CAALFM_CR10850CA	ATP16	57.6	52.44	0.135	0.251
SBDS domain-containing protein	Q59U89	CAALFM_C207630CA	orf19	46.66	42.43	0.137	0.252
Ribosomal 60S subunit protein L35A	A0A1D8PK30	CAALFM_C304960WA	RPL35	5.77	5.24	0.139	0.252
Elongation factor Tu	Q5ABC3	CAALFM_C100590WA	TUF1	29.17	26.45	0.141	0.252
Oxysterol-binding protein related protein	A0A1D8PE79	CAALFM_C108180CA	orf19	1.07	0.97	0.142	0.253
Uncharacterized protein	Q59Z37	CAALFM_C207080CA	orf19	1.07	0.97	0.142	0.253
Ras family GTPase	A0A1D8PS50	CAALFM_CR02140WA	RSR1	1.58	1.43	0.144	0.253
SUI1 domain-containing protein	Q59SK8	CAALFM_C400770CA	orf19	1.05	0.95	0.144	0.253
NADP-dependent alcohol dehydrogenase	Q5AC33	CAALFM_C602480WA	orf19	1.05	0.95	0.144	0.253
Stress protein DDR48	Q59X49	CAALFM_C209220WA	DDR48	277.9	251.06	0.147	0.254
Serine hydroxymethyltransferase	A0A1D8PRB3	CAALFM_C703330CA	SHM1	88.64	80.04	0.147	0.254
HABP4_PAI-RBP1 domain-containing protein	A0A1D8PK71	CAALFM_C304810CA	orf19	100.85	90.99	0.148	0.254
Cell surface mannoprotein MP65	Q59XX2	CAALFM_C210030CA	MP65	16.31	14.69	0.151	0.255
PWI domain-containing protein	A0A1D8PN50	CAALFM_C501310WA	orf19	0.5	0.45	0.152	0.255
Multiprotein-bridging factor 1	Q5A940	CAALFM_C101060WA	MBF1	47.88	43.05	0.153	0.255
Actin-related protein 8	A0A1D8PCJ9	CAALFM_C101700WA	ARP8	2.67	2.4	0.154	0.255
Csp37p	Q5A9D4	CAALFM_CR01470WA	CSP37	132.89	119.44	0.154	0.255
Malate dehydrogenase	Q5AMP4	CAALFM_C401900CA	MDH1-1	131.82	118.43	0.155	0.255
Hypoxanthine phosphoribosyltransferase	A0A1D8PGN7	CAALFM_C202740CA	HPT1	5.12	4.6	0.155	0.255
Glycine cleavage system H protein	Q5AKX1	CAALFM_C113680CA	GCV3	26.08	23.42	0.155	0.256
Uncharacterized protein	A0A1D8PLN9	CAALFM_C402860WA	orf19	7.58	6.8	0.157	0.256
Cta3p	A0A1D8PF53	CAALFM_C111540CA	CTA3	21.01	18.83	0.158	0.256
Uncharacterized protein	Q5ABU1	CAALFM_C603220WA	orf19	1.06	0.95	0.158	0.256
Hgt19p	Q5A7L9	CAALFM_C300220WA	HGT19	11.85	10.62	0.158	0.256
Hap2p	A0A1D8PE35	CAALFM_C107680WA	HAP2	4.09	3.66	0.160	0.257
Nop6p	Q5AAS4	CAALFM_C106740CA	NOP6	1.6	1.43	0.162	0.257

Phospho-2-dehydro-3-deoxyheptonate aldolase	A0A1D8PGI8	CAALFM_C202030WA	ARO3	7.55	6.74	0.164	0.258
Monothiol glutaredoxin	Q59PW1	CAALFM_C107630WA	orf19	7.05	6.28	0.167	0.258
3,4-dihydroxy-2-butanone 4-phosphate synthase	Q5A3V6	CAALFM_C112360CA	RIB3	8.59	7.65	0.167	0.258
H(+)-transporting V1 sector ATPase subunit E	A0A1D8PS38	CAALFM_CR01970CA	VMA4	26.32	23.42	0.168	0.259
GPI-anchored protein 52	Q59L72	CAALFM_C200100CA	PGA52	16.03	14.23	0.172	0.259
UV excision repair protein RAD23	A0A1D8PGE3	CAALFM_C201850WA	RAD23	30.49	27	0.175	0.260
Ifu5p	A0A1D8PS14	CAALFM_CR01730WA	IFU5	5.79	5.12	0.177	0.261
U6 snRNA-associated Sm-like protein LSm3	A0A1D8PTG5	CAALFM_CR07330WA	SMX4	5.79	5.12	0.177	0.261
Phosphoenolpyruvate carboxykinase	A0A1D8PRM7	CAALFM_CR00200WA	PCK1	35.13	31.02	0.180	0.261
Protein FYV4, mitochondrial	Q5A8X7	CAALFM_C101680CA	FYV4	5.89	5.2	0.180	0.261
Uncharacterized protein	A0A1D8PQC8	CAALFM_C604290WA	orf19	5.77	5.09	0.181	0.261
40S ribosomal protein S24	Q5A7K0	CAALFM_C300090WA	RPS24	16.84	14.85	0.181	0.262
40S ribosomal protein S0	O42817	CAALFM_C305370CA	RPS0	20.37	17.95	0.183	0.262
Ribosomal 40S subunit protein S19A	A0A1D8PK61	CAALFM_C305200WA	RPS19A	50.29	44.31	0.183	0.262
40S ribosomal protein S25	A0A1D8PNQ6	CAALFM_C503540CA	RPS25B	12.8	11.27	0.184	0.262
V-type proton ATPase subunit G	Q59U85	CAALFM_C207590WA	VMA10	4.85	4.27	0.184	0.262
Exosome non-catalytic core subunit	A0A1D8PD51	CAALFM_C103830CA	orf19	1.08	0.95	0.185	0.262
Uncharacterized protein	A0A1D8PMF0	CAALFM_C405900CA	orf19	1.08	0.95	0.185	0.262
Uncharacterized protein	A0A1D8PN59	CAALFM_C501430CA	orf19	1.08	0.95	0.185	0.262
Ribosomal protein P2B	Q5ANH5	CAALFM_C304680WA	RPP2B	8.71	7.65	0.187	0.263
pH-responsive protein 2	O13318	CAALFM_C100220WA	PHR2	22.16	19.44	0.189	0.263
Ssz1p	Q5A678	CAALFM_C404700WA	SSZ1	13.91	12.2	0.189	0.263
Histone H2A.2	Q59VP2	CAALFM_C104170CA	HTA2	11.35	9.95	0.190	0.263
RanBD1 domain-containing protein	A0A1D8PHF9	CAALFM_C205530CA	orf19	8.71	7.63	0.191	0.264
Deoxyhypusine hydroxylase	Q59Z14	CAALFM_C207290WA	LIA1	36.31	31.77	0.193	0.264
Uncharacterized protein	A0A1D8PF90	CAALFM_C111860WA	orf19	14.38	12.57	0.194	0.264
Vacuolar membrane protease	Q59RF7	CAALFM_C604650WA	CAALFM_C604650WA	1.66	1.45	0.195	0.265
Mannose-6-phosphate isomerase	P34948	CAALFM_C209640WA	PMI1	16.35	14.26	0.197	0.265
Translation initiation factor eIF4G	A0A1D8PI73	CAALFM_C208760CA	TIF4631	8.19	7.14	0.198	0.265
Chaperone ATPase	A0A1D8PTP9	CAALFM_CR08250CA	HSP104	25.76	22.45	0.198	0.265
F1F0 ATP synthase subunit 5	Q5A7P7	CAALFM_C300460WA	ATP5	58.4	50.86	0.199	0.266
Mrp8p	Q5A646	CAALFM_C404430WA	MRP8	3.8	3.3	0.204	0.266
40S ribosomal protein S27	A0A1D8PTI7	CAALFM_CR07630CA	RPS27	4.09	3.55	0.204	0.267
Ribosomal 60S subunit protein L33A	A0A1D8PHH4	CAALFM_C205710CA	orf19	6.64	5.76	0.205	0.267
GTP-Rho binding exocyst subunit	A0A1D8PMI1	CAALFM_C406250CA	SEC3	0.52	0.45	0.209	0.268
Dolichyl-phosphate-mannose--protein mannosyltransferase 1	O74189	CAALFM_C702890CA	PMT1	0.52	0.45	0.209	0.268
Probable kinetochore protein SPC25	Q59PT6	CAALFM_CR05840WA	SPC25	0.52	0.45	0.209	0.268
Thioredoxin	A0A1D8PU69	CAALFM_CR10350CA	TRX1	23.63	20.41	0.211	0.268
Metacaspase-1	Q5ANA8	CAALFM_C305190CA	MCA1	5.64	4.86	0.215	0.269
Mitochondrial nucleoid protein	Q59Z25	CAALFM_C207190CA	orf19	15.97	13.76	0.215	0.269
Superoxide dismutase	Q5A8Z4	CAALFM_C101520CA	SOD2	45.5	39.17	0.216	0.269
Pho100p	Q59WE5	CAALFM_C107430WA	PHO100	17.12	14.73	0.217	0.269
Protein SUR7	Q5A4M8	CAALFM_C601720CA	SUR7	8.94	7.67	0.221	0.270
Ribose-5-phosphate isomerase	Q5AJ92	CAALFM_C301480CA	RKI1	8.04	6.87	0.227	0.272
Peroxiredoxin	Q5AF44	CAALFM_C402410CA	AHP1	66.17	56.43	0.230	0.272
Cytochrome b-c1 complex subunit 2, mitochondrial	P83782	CAALFM_C503350WA	QCR2	73.08	62.27	0.231	0.273
Pex19p	Q5A330	CAALFM_CR08600CA	PEX19	32.63	27.78	0.232	0.273
Acetyl-CoA carboxylase	A0A1D8PRR7	CAALFM_CR00640WA	ACC1	6.16	5.24	0.233	0.273
Blood-induced peptide 1	P0CT51	CAALFM_C112850WA	BLP1	20.37	17.31	0.235	0.273
Snl1p	Q59NB3	CAALFM_C110530WA	SNL1	0.53	0.45	0.236	0.274
Carboxypeptidase	A0A1D8PRC2	CAALFM_C703360WA	CPY1	41.47	35.2	0.237	0.274
SCP2 domain-containing protein	Q5AJ84	CAALFM_C301420CA	orf19	6.31	5.35	0.238	0.274
Peroxiredoxin	A0A1D8PR39	CAALFM_C702390WA	AHP2	4.33	3.67	0.239	0.274
Uncharacterized protein	A0A1D8PF42	CAALFM_C111200WA	orf19	871.72	737.84	0.241	0.275
Succinate--CoA ligase [ADP-forming] subunit beta, mitochondrial	A0A1D8PTB5	CAALFM_CR06760CA	LSC2	59.96	50.44	0.249	0.277
Ribosomal 60S subunit protein L28	A0A1D8PSC5	CAALFM_CR03030CA	RPL28	13.91	11.7	0.250	0.277
40S ribosomal protein S8	Q59T44	CAALFM_C205610CA	RPS8A	46.95	39.42	0.252	0.277
Heat shock protein SSA2	P46587	CAALFM_C104300CA	SSA2	139.64	117.08	0.254	0.278
Tos1p	A0A1D8PJA8	CAALFM_C301550CA	TOS1	10	8.37	0.257	0.278
Ribonucloprotein	Q5A6M9	CAALFM_CR04360CA	NHP2	8.19	6.85	0.258	0.279
Transaldolase	Q5A017	CAALFM_CR03720WA	TAL1	52.16	43.61	0.258	0.279
Fumarase	Q5A6L1	CAALFM_CR04530WA	FUM11	6.82	5.7	0.259	0.279
Ubiquitin-conjugating enzyme E2 2	O74201	CAALFM_C703870WA	UBC2	4.85	4.05	0.260	0.279
Chromatin modification-related protein EAF7	Q5A6Q7	CAALFM_CR04130CA	EAF7	3.07	2.56	0.262	0.280
Wor4p	Q5ADX8	CAALFM_C307730WA	WOR4	3.66	3.05	0.263	0.280
Hsp70 family ATPase	A0A1D8PRH1	CAALFM_C704010WA	SSQ1	1.08	0.9	0.263	0.280
Protein VTS1	Q5AI80	CAALFM_C102850WA	VTS1	12.22	10.18	0.264	0.280
Protein transport protein SSO2	Q59YF0	CAALFM_C209740WA	SSO2	19.57	16.29	0.265	0.280
Secreted beta-glucosidase SIM1	Q5AKU5	CAALFM_C113940WA	SIM1	4.31	3.58	0.268	0.281
Single-stranded telomeric DNA-binding/mRNA-binding protein	A0A1D8PQM1	CAALFM_C700440CA	GBP2	48.24	40.03	0.269	0.281
SAP domain-containing protein	A0A1D8PIF1	CAALFM_C209040WA	orf19	17.67	14.66	0.269	0.281
Hxt5p	A0A1D8PSH1	CAALFM_CR03450WA	HXT5	4.63	3.84	0.270	0.281
Uncharacterized protein	Q5A6L4	CAALFM_CR04490CA	CAALFM_CR04490CA	3.16	2.62	0.270	0.281
Uncharacterized protein	Q59YF2	CAALFM_C209760WA	orf19	8.23	6.81	0.273	0.282
Kis1p	Q59X47	CAALFM_C209230CA	KIS1	8.22	6.8	0.274	0.282
HMG box domain-containing protein	A0A1D8PJ67	CAALFM_C301210CA	orf19	4.3	3.55	0.277	0.283
Zuotin	Q5AF98	CAALFM_C402870CA	ZUO1	11.78	9.72	0.277	0.283
Peptidylprolyl isomerase	A0A1D8PFM9	CAALFM_C113260WA	orf19	7.46	6.15	0.279	0.283
Sm-like protein	A0A1D8PNP9	CAALFM_C503400CA	orf19	6.49	5.35	0.279	0.283
Ribosomal 60S subunit protein L34B	A0A1D8PDZ1	CAALFM_C106890CA	orf19	5.16	4.25	0.280	0.284
Ribosomal 40S subunit protein S5	Q5AG43	CAALFM_C503070WA	RPS5	37.84	31	0.288	0.285
Ribosomal 60S subunit protein L12A	Q5AJF7	CAALFM_C302110WA	RPL12	25.71	21.03	0.290	0.286
Pst2p	Q59Y37	CAALFM_C208640CA	PST2	28.42	23.24	0.290	0.286
Gvp36p	Q5A473	CAALFM_C405550CA	GVP36	23.81	19.44	0.293	0.286
Decapping enzyme complex catalytic subunit	A0A1D8PTQ2	CAALFM_CR08120CA	DCP2	3.74	3.05	0.294	0.287
Transcriptional regulator of yeast form adherence 4	Q5ANC8	CAALFM_C305050WA	TRY4	3.74	3.05	0.294	0.287
Pyridoxamine-phosphate oxidase	A0A1D8PSU2	CAALFM_CR04590CA	PDX3	5.79	4.71	0.298	0.288
Uncharacterized protein	A0A1D8PQ38	CAALFM_C603130WA	orf19	8.43	6.85	0.299	0.288
Proteasome regulatory particle lid subunit	A0A1D8PMQ0	CAALFM_C406880CA	orf19	5.79	4.7	0.301	0.288
Ribosomal 60S subunit protein L26B	A0A1D8PCQ5	CAALFM_C102330CA	orf19	18.23	14.78	0.303	0.289
Mci4p	Q5A995	CAALFM_CR01740WA	MCI4	10.82	8.75	0.306	0.290
Eukaryotic translation initiation factor 3 subunit J	Q5ACM9	CAALFM_CR10370WA	HCR1	14.6	11.8	0.307	0.290
Pst1p	A0A1D8PHR5	CAALFM_C206870CA	PST1	76.19	61.53	0.308	0.290
Age3p	A0A1D8PCP6	CAALFM_C102260CA	AGE3	8.19	6.61	0.309	0.290
Abp1p	Q5AFA8	CAALFM_C402940WA	ABP1	88.95	71.75	0.310	0.290
zf-CHCC domain-containing protein	Q59Y38	CAALFM_C208650WA	orf19	18.38	14.8	0.313	0.291

Non-histone chromosomal protein 6	Q9UVL1	CAALFM_C401700CA	NHP6	13.93	11.2	0.315	0.291
Mannan endo-1,6-alpha-mannosidase DFG5	Q5ACZ2	CAALFM_C200520WA	DFG5	4.85	3.89	0.318	0.292
Proteasome regulatory particle base subunit	A0A1D8PMQ4	CAALFM_C406870WA	RPT5	15.26	12.24	0.318	0.292
Glutaredoxin domain-containing protein	A0A1D8PPH3	CAALFM_C600910CA	orf19	106.66	85.49	0.319	0.292
GTP-binding protein RHO1	O42825	CAALFM_CR02860WA	RHO1	5.37	4.3	0.321	0.293
Tubulin-binding prefolding complex subunit	A0A1D8PF30	CAALFM_C111110CA	orf19	3.16	2.53	0.321	0.293
Formate dehydrogenase	Q59QN6	CAALFM_CR05170CA	FDH1	21.97	17.57	0.322	0.293
Trx2p	Q5A1L9	CAALFM_C500910CA	TRX2	3.57	2.85	0.325	0.294
Ubiquitin-binding protein	A0A1D8PDI5	CAALFM_C105170CA	CUE5	49.12	39.19	0.326	0.294
Uncharacterized protein	A0A1D8PS81	CAALFM_CR02510WA	CAALFM_CR02510WA	2.57	2.05	0.326	0.294
Heat shock protein SSA1	P41797	CAALFM_C113480WA	SSA1	177.3	141.21	0.328	0.294
Nuo2p	Q5AEI1	CAALFM_C302940CA	NUO2	10.52	8.37	0.330	0.295
Uncharacterized protein	Q59WV9	CAALFM_C600920WA	orf19	5.12	4.07	0.331	0.295
Uncharacterized protein	A0A1D8PHY0	CAALFM_C207550WA	orf19	5.41	4.3	0.331	0.295
Ribosome biogenesis protein ALB1	Q5AFG1	CAALFM_C700160CA	ALB1	6.77	5.38	0.332	0.295
U4/U6-U5 snRNP complex subunit	A0A1D8PRP0	CAALFM_CR00240WA	LSM6	15.31	12.12	0.337	0.296
Candidapepsin-9	Q59SU1	CAALFM_C303870CA	SAP9	12.86	10.18	0.337	0.296
Png2p	A0A1D8PHH2	CAALFM_C205660WA	PNG2	25.23	19.89	0.343	0.298
Peptidyl-prolyl cis-trans isomerase D	Q5ACI8	CAALFM_CR10670WA	CPR6	19.44	15.3	0.346	0.298
Agglutinin-like protein 4	A0A1D8PQB9	CAALFM_C604130CA	ALS4	12.32	9.69	0.346	0.298
6-phosphogluconolactonase-like protein	Q59PZ6	CAALFM_CR06700CA	SOL3	12.99	10.21	0.347	0.299
Protein MAK16	Q59P36	CAALFM_C206360CA	MAK16	3.07	2.41	0.349	0.299
Histone H2B.1	P48989	CAALFM_C303900CA	HTB1	29.49	23.12	0.351	0.300
Histone H2B.2	Q59VP1	CAALFM_C104180WA	HTB2	29.49	23.12	0.351	0.300
cAMP-dependent protein kinase regulatory subunit	Q9HEW1	CAALFM_C201110CA	BCY1	18.51	14.51	0.351	0.300
Uncharacterized protein	A0A1D8PHS1	CAALFM_C206850WA	orf19	6.64	5.2	0.353	0.300
Mitochondrial 54S ribosomal protein YmL37	Q59VH8	CAALFM_C104880CA	MRPL37	3.07	2.4	0.355	0.300
Endopolyphosphatase	A0A1D8PQR0	CAALFM_C700980WA	PHM5	2.61	2.04	0.356	0.301
Ferulic acid decarboxylase 1	A0A1D8PQ75	CAALFM_C603620CA	FDC1	4.59	3.58	0.359	0.301
mRNA stability protein	Q5AEI3	CAALFM_C302920WA	orf19	6.72	5.24	0.359	0.301
Eukaryotic translation initiation factor 5A	O94083	CAALFM_C601610WA	ANB1	363.87	283.49	0.360	0.302
Eukaryotic translation initiation factor 3 subunit I	Q5AI86	CAALFM_C102790WA	TIF34	30.76	23.96	0.360	0.302
Uncharacterized protein	A0A1D8PD02	CAALFM_C103240WA	orf19	3.25	2.53	0.361	0.302
1,2-dihydroxy-3-keto-5-methylthiopentene dioxygenase	Q59WJ5	CAALFM_C111100WA	ADI1	3.25	2.53	0.361	0.302
Uncharacterized protein	Q59WV8	CAALFM_C600930CA	orf19	23.57	18.33	0.363	0.302
TFIID_20kDa domain-containing protein	Q5ADM2	CAALFM_C306820CA	TAF12	4.63	3.6	0.363	0.302
Catabolic 3-dehydroquinase	Q59Z17	CAALFM_C207260CA	DQD1	17.18	13.32	0.367	0.303
Enhancer of mRNA-decapping protein 1	Q5ALP1	CAALFM_C202190CA	EDC1	5.26	4.07	0.370	0.304
Epsin	A0A1D8PGB7	CAALFM_C201390WA	orf19	9.23	7.13	0.372	0.304
Kel1p	Q5AB80	CAALFM_C100140WA	KEL1	4.63	3.57	0.375	0.305
Proteasome regulatory particle lid subunit	A0A1D8PDB0	CAALFM_C104230WA	orf19	18.36	14.15	0.376	0.305
Ribosomal protein P1B	A0A1D8PRG5	CAALFM_C703920CA	RPP1B	8.59	6.61	0.378	0.306
Asr1p	A0A1D8PEZ0	CAALFM_C110740CA	ASR1	38.39	29.53	0.379	0.306
Histone H2A.Z-specific chaperone CHZ1	Q59RN6	CAALFM_CR07230WA	CHZ1	12.59	9.68	0.379	0.306
60S ribosomal protein L27	A0A1D8PFG4	CAALFM_C112390CA	RPL27A	21.13	16.14	0.389	0.308
Pho112p	A0A1D8PS71	CAALFM_CR02400WA	PHO112	22.01	16.81	0.389	0.308
Inorganic pyrophosphatase	P83777	CAALFM_C208810CA	IPP1	65.42	49.9	0.391	0.308
Uncharacterized protein	A0A1D8PEU9	CAALFM_C110350CA	orf19	3.74	2.85	0.392	0.309
Lipid-binding protein	A0A1D8PDD1	CAALFM_C104680WA	PIL1	156.19	119	0.392	0.309
Heat shock protein homolog SSE1	Q96VB9	CAALFM_C106100CA	MSI3	31.22	23.78	0.393	0.309
60S ribosomal protein L36	A0A1D8PH21	CAALFM_C203960WA	RPL39	17.9	13.62	0.394	0.309
F-actin-capping protein subunit alpha	Q5A893	CAALFM_CR01180WA	CAP01	10.23	7.78	0.395	0.309
Uncharacterized protein	A0A1D8PPF1	CAALFM_C600680CA	orf19	3.16	2.4	0.397	0.310
Tom20p	Q5AIA0	CAALFM_C102640CA	TOM20	6.44	4.89	0.397	0.310
Gcf1p	Q59QB8	CAALFM_C108550CA	GCF1	14.07	10.68	0.398	0.310
3'(2'),5'-bisphosphate nucleotidase	Q59XQ1	CAALFM_C601030WA	HAL22	5.13	3.89	0.399	0.310
Peroxiredoxin	A0A1D8PDN3	CAALFM_C105410CA	TRP99	25.06	18.92	0.406	0.312
D-arabinose 1-dehydrogenase	A0A1D8PI24	CAALFM_C208130WA	ARA1	2.57	1.94	0.406	0.312
Ribosomal 60S subunit protein L2A	A0A1D8PF08	CAALFM_C111060CA	RPL2	24.28	18.31	0.407	0.312
Rbt7p	A0A1D8PLR0	CAALFM_C403100WA	RBT7	3.78	2.85	0.407	0.312
Hsp12p	A0A1D8PNC7	CAALFM_C502080CA	HSP12	69.93	52.71	0.408	0.312
Uncharacterized protein	A0A1D8PQ55	CAALFM_C603380WA	orf19	9.73	7.33	0.409	0.312
Mitochondrial 54S ribosomal protein MRPL50	A0A1D8PHU0	CAALFM_C207030CA	orf19	14.19	10.68	0.410	0.313
JAB_MPN domain-containing protein	Q5AG96	CAALFM_C502660CA	orf19	5.41	4.07	0.411	0.313
Kre9p	Q5ANN9	CAALFM_C304180WA	KRE9	6.97	5.24	0.412	0.313
Oxysterol-binding protein	A0A1D8PHG1	CAALFM_C205720CA	orf19	2.05	1.54	0.413	0.313
Serine/threonine protein kinase	A0A1D8PKB4	CAALFM_C305650WA	YCK2	2.05	1.54	0.413	0.313
Imidazole glycerol phosphate synthase hisHF	A0A1D8PRE9	CAALFM_C703720CA	HIS7	2.05	1.54	0.413	0.313
Polyadenylate-binding protein, cytoplasmic and nuclear	Q5AI15	CAALFM_C103370WA	PAB1	41.93	31.49	0.413	0.313
Prefoldin subunit 4	Q5A435	CAALFM_C405860WA	orf19	3.8	2.85	0.415	0.314
Uncharacterized protein	Q5ALN3	CAALFM_C202260WA	orf19	18.98	14.23	0.416	0.314
Bud21p	Q5A7N6	CAALFM_C300370CA	BUD21	2.03	1.52	0.417	0.314
Actin cytoskeleton-regulatory complex protein PAN1	Q5AHB1	CAALFM_C203380WA	PAN1	17.9	13.4	0.418	0.314
Uncharacterized protein	A0A1D8PKZ4	CAALFM_C400230WA	orf19	2.06	1.54	0.420	0.315
Small heat shock protein 21	Q5AHH4	CAALFM_C204010CA	HSP21	84.35	63.06	0.420	0.315
Uncharacterized protein	A0A1D8PJ76	CAALFM_C301520CA	orf19	12.94	9.66	0.422	0.315
Extracellular glycosidase UTR2	Q5AJC0	CAALFM_C301730CA	UTR2	12.31	9.19	0.422	0.315
Peroxiredoxin	Q5A5A0	CAALFM_C702810WA	PRX1	25.44	18.99	0.422	0.315
Predicted GPI-anchored protein 45	Q5AA33	CAALFM_C105960WA	PGA45	4.09	3.05	0.423	0.316
GRIP domain-containing protein	A0A1D8PHG9	CAALFM_C205800CA	orf19	14.32	10.66	0.426	0.316
Rfa2p	Q59Z33	CAALFM_C207120WA	RFA2	19.96	14.82	0.430	0.317
Hsp70 family ATPase	Q5A397	CAALFM_CR08090WA	SSB1	144.73	107.44	0.430	0.317
Translationally-controlled tumor protein homolog	Q5A860	CAALFM_CR00860CA	TMA19	124.67	92.53	0.430	0.317
ATP synthase subunit d, mitochondrial	Q59PV8	CAALFM_C107600WA	ATP7	24.19	17.95	0.430	0.317
Bzz1p	A0A1D8PJ75	CAALFM_C301500CA	BZZ1	2.05	1.52	0.432	0.318
Ribosomal 40S subunit protein S28B	A0A1D8PQN0	CAALFM_C700710WA	RPS28B	25.47	18.85	0.434	0.318
Ribosomal 60S subunit protein L38	A0A1D8PG16	CAALFM_C200210WA	RPL38	9.21	6.81	0.436	0.318
Copper chaperone	Q59WC0	CAALFM_C107180WA	CCS1	17.87	13.21	0.436	0.319
Cytochrome c oxidase subunit VI	A0A1D8PH08	CAALFM_C203470CA	COX6	22.18	16.39	0.436	0.319
14-3-3 protein homolog	O42766	CAALFM_C103220CA	BMH1	40.51	29.93	0.437	0.319
RNA polymerase II degradation factor 1	Q5AMM4	CAALFM_C401720CA	DEF1	31.7	23.42	0.437	0.319
Non-classical export protein 102	Q5ANE3	CAALFM_C304910CA	NCE102	6.49	4.79	0.438	0.319
RRM domain-containing protein	A0A1D8PJE6	CAALFM_C302160CA	orf19	2.06	1.52	0.439	0.319
Mdg1p	A0A1D8PFV7	CAALFM_C114290CA	MDG1	102.83	75.85	0.439	0.319
Eukaryotic translation initiation factor 3 subunit G	Q59ZV5	CAALFM_C114260CA	TIF35	5.77	4.25	0.441	0.320

ANK_REP_REGION domain-containing protein	A0A1D8PRI6	CAALFM_C704140CA	orf19	12.44	9.16	0.442	0.320
Uncharacterized protein	Q5A5Q4	CAALFM_C210670WA	orf19	21.47	15.79	0.443	0.320
Histone H4	Q59VN4	_C104240CACAALFM_CR0	HHF1	10.58	7.78	0.444	0.320
mRNA-binding protein	A0A1D8PCH2	CAALFM_C101470WA	orf19	13.14	9.66	0.444	0.320
Peptide hydrolase	Q5AA00	CAALFM_C105670WA	orf19	11.29	8.28	0.447	0.321
DNA-directed RNA polymerase core subunit	Q59QT0	CAALFM_C503360WA	RPO26	2.15	1.57	0.454	0.323
Ran GTPase-binding protein	A0A1D8PRQ3	CAALFM_CR00580WA	YRB1	25.08	18.31	0.454	0.323
Mitochondrial 54S ribosomal protein YmL10/YmL18	Q59ZI7	CAALFM_C205000CA	MRPL10	4.59	3.35	0.454	0.323
Asm3p	Q5ABD3	CAALFM_C100680WA	ASM3	2.11	1.54	0.454	0.323
Septin CDC11	G1UB61	CAALFM_C500070WA	CDC11	4.63	3.37	0.458	0.324
Cup2p	Q5AKX6	CAALFM_C113640WA	CUP2	2.05	1.49	0.460	0.324
Glg2p	A0A1D8PKH2	CAALFM_C306450WA	GLG2	2.67	1.94	0.461	0.324
Dap1p	Q5A6R5	CAALFM_CR04060CA	DAP1	2.67	1.94	0.461	0.324
Pyridoxal 5'-phosphate synthase	Q5AGF9	CAALFM_C502140CA	orf19	6.69	4.86	0.461	0.324
RNA-binding protein	A0A1D8PK11	CAALFM_C304590WA	orf19	6.49	4.71	0.463	0.325
GrpE protein homolog	Q5A9E1	CAALFM_CR01390WA	MGE1	27.34	19.84	0.463	0.325
Isa2p	Q5ADN0	CAALFM_C306900WA	ISA2	9.11	6.61	0.463	0.325
Fe-S cluster assembly protein DRE2	Q5A218	CAALFM_CR02650CA	DRE2	5.62	4.07	0.466	0.325

Rim1p	Q5AA01	CAALFM_C105680CA	RIM1	23.53	17.04	0.466	0.325
V-type proton ATPase subunit F	A0A1D8PH17	CAALFM_C204150CA	VMA7	4.76	3.44	0.469	0.326
Beta-hexosaminidase	A0A1D8PNR7	CAALFM_C503610WA	HEX1	2.63	1.9	0.469	0.326
Chitinase 3	P40954	CAALFM_CR10110WA	CHT3	16.23	11.7	0.472	0.327
E2 ubiquitin-conjugating protein	A0A1D8PFF9	CAALFM_C112650CA	orf19	6.69	4.82	0.473	0.327
SH3 domain-containing protein	A0A1D8PH89	CAALFM_C204850CA	orf19	2.11	1.52	0.473	0.327
Nascent polypeptide-associated complex subunit beta	Q59TU0	CAALFM_C111650WA	EGD1	16.3	11.74	0.473	0.327
Acid phosphatase	Q59UY6	CAALFM_CR02180WA	PHO113	24.05	17.29	0.476	0.328
E2 ubiquitin-conjugating protein	A0A1D8PQD4	CAALFM_C604280WA	orf19	20.56	14.78	0.476	0.328
Mitochondrial 54S ribosomal protein YmL2	A0A1D8PRG0	CAALFM_C703790WA	MRP7	8.56	6.15	0.477	0.328
Nicotinate-nucleotide pyrophosphorylase [carboxylating]	A0A1D8PE45	CAALFM_C107840WA	orf19	63.98	45.83	0.481	0.329
40S ribosomal protein S7	Q5AJ93	CAALFM_C301490WA	RPS7A	45.27	32.39	0.483	0.329
Cytochrome c oxidase subunit	Q5ALV9	CAALFM_C201590WA	COX13	2.63	1.88	0.484	0.329
Uncharacterized protein	A0A1D8PJA9	CAALFM_C301890CA	orf19	20.56	14.69	0.485	0.330
Bud22p	A0A1D8PRR2	CAALFM_CR00680WA	BUD22	2.16	1.54	0.488	0.330
Putative phosphoric monoester hydrolase	A0A1D8PHU6	CAALFM_C207140WA	orf19	20.03	14.28	0.488	0.330
Covalently-linked cell wall protein 14	Q5AFN8	CAALFM_C700860WA	SSR1	6.44	4.59	0.489	0.330
Nfu_N domain-containing protein	Q59N44	CAALFM_CR07670WA	CAALFM_CR07670WA	10.25	7.3	0.490	0.331
40S ribosomal protein S30	A0A1D8PSK2	CAALFM_CR03770CA	RPS30	6.14	4.37	0.491	0.331
Copper transport protein CTR1	Q59NP1	CAALFM_C600790CA	CTR1	10.73	7.63	0.492	0.331
Ribosomal 40S subunit protein S17B	A0A1D8PEY9	CAALFM_C110870WA	RPS17B	33.26	23.64	0.493	0.331
Type II HSP40 co-chaperone	Q59V92	CAALFM_CR06080WA	SIS1	12.41	8.82	0.493	0.331
Glycerophosphodiester transporter GIT2	A0A1D8PN12	CAALFM_C500890CA	GIT2	3.57	2.53	0.497	0.332
Pdx1p	Q5AKV6	CAALFM_C113830CA	PDX1	38.84	27.49	0.499	0.333
Translation machinery-associated protein 20	Q5A199	CAALFM_C406170CA	orf19	2.15	1.52	0.500	0.333
rRNA methyltransferase	Q5A0V9	CAALFM_C406720WA	NOP1	9.71	6.85	0.503	0.334
Formate dehydrogenase	A0A1D8PIL8	CAALFM_C210070WA	orf19	4.88	3.44	0.505	0.334
Fructose-bisphosphate aldolase	Q9URB4	CAALFM_C401750CA	FBA1	143.91	101.4	0.505	0.334
Cytochrome c oxidase subunit Va	Q5APK5	CAALFM_C109030CA	COX5	8.66	6.09	0.508	0.335
Uncharacterized protein	A0A1D8PQE6	CAALFM_C604420WA	orf19	8.11	5.7	0.509	0.335
Polyamine acetyltransferase	A0A1D8PFY0	CAALFM_C114500CA	orf19	13.78	9.68	0.510	0.335
Asr2p	A0A1D8PTU7	CAALFM_CR08890CA	ASR2	75.77	53.19	0.511	0.335
40S ribosomal protein S12	Q5ADQ6	CAALFM_C307150CA	RPS12	77.84	54.6	0.512	0.336
Uncharacterized protein	A0A1D8PD97	CAALFM_C104360CA	orf19	6.19	4.34	0.512	0.336
Mitochondrial import inner membrane translocase subunit TIM13	Q5AF54	CAALFM_C402480CA	TIM13	4.71	3.3	0.513	0.336
Nfu_N domain-containing protein	A0A1D8PG46	CAALFM_C200600CA	orf19	7.58	5.31	0.514	0.336
Nucleolar protein 16	Q59YD8	CAALFM_C209660WA	NOP16	3.66	2.56	0.516	0.336
Crd2p	A0A1D8PL78	CAALFM_C401160WA	CRD2	8.71	6.09	0.516	0.337
Asr3p	A0A1D8PH00	CAALFM_C203790CA	ASR3	4.82	3.37	0.516	0.337
Rho family GTPase	A0A1D8PH96	CAALFM_C205030CA	RHO3	2.05	1.43	0.520	0.337
Ubiquitin-ribosomal 40S subunit protein S31 fusion protein	Q5A109	CAALFM_C407180WA	UBI3	69.13	48.11	0.523	0.338
Kri1_C domain-containing protein	A0A1D8PJF3	CAALFM_C302350WA	orf19	10.23	7.11	0.525	0.339
Mannan endo-1,6-alpha-mannosidase DCW1	Q5AD78	CAALFM_C201360CA	DCW1	5.27	3.66	0.526	0.339
Ribosomal 60S subunit protein L25	A0A1D8PPS1	CAALFM_C601970CA	RPL25	18.46	12.8	0.528	0.339
Bfr1p	Q5AMT7	CAALFM_C402270CA	BFR1	69.16	47.9	0.530	0.340
Cytosine deaminase	P78594	CAALFM_C600620WA		11.76	8.14	0.531	0.340
TATA-binding protein-associated factor	A0A1D8PH31	CAALFM_C204220CA	TAF14	8.11	5.61	0.532	0.340
Cytochrome c oxidase subunit IV	Q5ALV5	CAALFM_C201620WA	COX4	42.12	29.06	0.536	0.341
Ecm33p	A0A1D8PCY4	CAALFM_C103190CA	ECM33	168.6	116.09	0.538	0.342
mRNA-binding protein	A0A1D8PMA5	CAALFM_C405370WA	PUF3	3.68	2.53	0.541	0.342
Rod1p	A0A1D8PGG1	CAALFM_C201970CA	ROD1	1.53	1.05	0.543	0.343
Tif11p	Q5A5P8	CAALFM_C210710WA	TIF11	13.19	9.04	0.545	0.343
Proteasome regulatory particle base subunit	Q59X29	CAALFM_C206150CA	RPN10	7.88	5.38	0.551	0.344
Peroxin-14	A0A1D8PSW1	CAALFM_CR04930WA	PEX14	34.31	23.42	0.551	0.344
Uncharacterized protein	Q59WJ7	CAALFM_C111120CA	orf19	17.45	11.89	0.554	0.345
Uncharacterized protein	Q5AC28	CAALFM_C602530CA	orf19	31.45	21.41	0.555	0.345
Gca2p	A0A1D8PEW1	CAALFM_C110550CA	GCA2	91.95	62.49	0.557	0.346
FG-nucleoporin	A0A1D8PGV7	CAALFM_C203280WA	NSP1	81.72	55.46	0.559	0.346
Actin cytoskeleton-regulatory complex protein END3	Q5AJ82	CAALFM_C301400WA	END3	19.4	13.16	0.560	0.346
Tif3p	A0A1D8PPQ1	CAALFM_C601630WA	TIF3	82.48	55.91	0.561	0.347
Histone H2A.1	Q59SU5	CAALFM_C303910WA	HTA1	8.66	5.87	0.561	0.347
Cta1p	Q5A4P1	CAALFM_C601830WA	CTA1	1.55	1.05	0.562	0.347
Phosphopantothenoylcysteine decarboxylase complex subunit	Q5A868	CAALFM_CR00980CA	CAB3	1.55	1.05	0.562	0.347
Sbp1p	Q5ANP6	CAALFM_C304090WA	SBP1	82.08	55.46	0.566	0.348
Agglutinin-like protein 1	Q5A8T4	CAALFM_C603700WA	ALS1	17.37	11.72	0.568	0.348
60S acidic ribosomal protein P1-A	Q9HFQ7	CAALFM_C103010WA	RPP1A	13.14	8.86	0.569	0.348
Ribosomal 60S subunit protein L31B	A0A1D8PHF5	CAALFM_C205410WA	orf19	34.75	23.4	0.571	0.349
WD_REPEATS_REGION domain-containing protein	A0A1D8PP79	CAALFM_C505340WA	orf19	1.52	1.02	0.576	0.350
Phosphomannomutase	P31353	CAALFM_C102480WA	PMM1	24.36	16.32	0.578	0.350
Carboxypeptidase	A0A1D8PN69	CAALFM_C501450WA	PRC2	25.04	16.77	0.578	0.350
Calmodulin	A0A1D8PMF8	CAALFM_C406030WA	CMD1	14.74	9.85	0.582	0.351
ANK_REP_REGION domain-containing protein	A0A1D8PHS4	CAALFM_C207000WA	orf19	8.38	5.59	0.584	0.352
Uncharacterized protein	Q59MV4	CAALFM_CR07830CA	CAALFM_CR07830CA	1.53	1.02	0.585	0.352
GYF domain-containing protein	A0A1D8PMM2	CAALFM_C406680CA	orf19	29.17	19.44	0.586	0.352
PITH domain-containing protein	Q5AH92	CAALFM_C203260WA	orf19	13.84	9.19	0.591	0.353
Uncharacterized protein	A0A1D8PPZ0	CAALFM_C602740WA	orf19	2.16	1.43	0.595	0.354
Glutamate decarboxylase	A0A1D8PF79	CAALFM_C111660WA	GAD1	217.02	143.6	0.596	0.354

Peptidyl-prolyl cis-trans isomerase	P22011	CAALFM_C702380CA	CYP1	325.47	215.11	0.597	0.355
Hsp70 nucleotide exchange factor FES1	Q59NN8	CAALFM_C600760WA	FES1	26.23	17.31	0.600	0.355
Uncharacterized protein	A0A1D8PMK4	CAALFM_C406470WA	orf19	5.79	3.82	0.600	0.355
Mitochondrial 37S ribosomal protein YMR31	A0A1D8PND4	CAALFM_C502190CA	orf19	5.79	3.82	0.600	0.355
Sgt2p	Q5A0I8	CAALFM_C202830CA	SGT2	41.05	27.08	0.600	0.355
Uncharacterized protein	A0A1D8PNB1	CAALFM_C501890WA	orf19	4.63	3.05	0.602	0.356
Hsp90 co-chaperone Cdc37	Q8X1E6	CAALFM_C602610CA	CDC37	9.64	6.35	0.602	0.356
Hgt2p	Q59VZ0	CAALFM_C102110CA	HGT2	11.21	7.38	0.603	0.356
Mitochondrial 37S ribosomal protein RSM18	Q59KY7	CAALFM_C104370CA	orf19	5.12	3.37	0.603	0.356
ADF-H domain-containing protein	A0A1D8PPG2	CAALFM_C600660CA	orf19	1.55	1.02	0.604	0.356
Uncharacterized protein	Q5A5S3	CAALFM_C210540WA	MIG2	2.16	1.42	0.605	0.357
Actin-related protein 2/3 complex subunit 3	Q59WT0	CAALFM_C601140CA	ARC18	1.6	1.05	0.608	0.357
Protein phosphatase regulator	A0A1D8PS15	CAALFM_CR01610CA	SHP1	48.11	31.54	0.609	0.357
Fum12p	A0A1D8PKV4	CAALFM_C307640CA	FUM12	40.86	26.77	0.610	0.358
FG-nucleoporin	Q5ANH3	CAALFM_C304700WA	orf19	3.07	2.01	0.611	0.358
Uncharacterized protein	A0A1D8PJ10	CAALFM_C300850CA	orf19	147.92	96.65	0.614	0.358
Phosphoglycerate kinase	P46273	CAALFM_C600750CA	PGK1	221.61	144.24	0.620	0.360
Hgt8p	A0A1D8PG82	CAALFM_C201010WA	HGT8	11.23	7.3	0.621	0.360
RNA-binding signal recognition particle subunit	Q5A9A9	CAALFM_CR01660CA	SEC65	1.57	1.02	0.622	0.360
Uncharacterized protein	A0A1D8PFU8	CAALFM_C114090WA	orf19	9.47	6.15	0.623	0.360
Transcription factor RBF1	Q5ABZ2	CAALFM_C602840CA	RBF1	8.07	5.24	0.623	0.361
rRNA-processing protein	A0A1D8PLN1	CAALFM_C402790CA	SAS10	11.35	7.34	0.629	0.362
Sec63 complex subunit	Q59SJ2	CAALFM_C400630CA	SEC72	1.61	1.04	0.631	0.362
E3 ubiquitin-protein ligase BRE1	Q5A4X0	CAALFM_C500210CA	BRE1	3.16	2.04	0.631	0.362
Enhancer of translation termination 1	Q5A0J9	CAALFM_C202930CA	ETT1	11.95	7.71	0.632	0.363
Uncharacterized protein	A0A1D8PNY3	CAALFM_C504410CA	orf19	5.26	3.39	0.634	0.363
Thiosulfate sulfurtransferase	A0A1D8PTD8	CAALFM_CR07020WA	CAALFM_CR07020WA	6.32	4.07	0.635	0.363
Ofr1p	Q5A784	CAALFM_C108060WA	OFR1	14.2	9.14	0.636	0.363
Peptidyl-prolyl cis-trans isomerase	Q5ALM6	CAALFM_C202320CA	CPR3	92.71	59.67	0.636	0.363
Protein disulfide isomerase	A0A1D8PR99	CAALFM_C703250CA	PDI1	134.08	85.99	0.641	0.365
Succinate--CoA ligase [ADP-forming] subunit alpha, mitochondrial	Q5A8X6	CAALFM_C101690CA	LSC1	23.01	14.74	0.643	0.365
Ferroxidase	A0A1D8PPC9	CAALFM_C600440CA	FET34	8.75	5.59	0.646	0.366
FK506-binding protein 1	P28870	CAALFM_C702570CA	RBP1	26.73	17.04	0.650	0.366
Uncharacterized protein	Q5A796	CAALFM_C107950CA	orf19	1.6	1.02	0.650	0.366
Dihydrolipoyl dehydrogenase	Q59RQ6	CAALFM_CR07400CA	LPD1	1069.75	681.61	0.650	0.367
Dot6p	A0A1D8PS02	CAALFM_CR01580CA	DOT6	6.44	4.1	0.651	0.367
Nucleolar protein 12	Q5AHI7	CAALFM_C204120CA	NOP12	3.77	2.4	0.652	0.367
SUMO family protein	Q59W54	CAALFM_C111330CA	SMT3	8.38	5.31	0.658	0.368
Uncharacterized protein	Q5AH10	CAALFM_C701930CA	orf19	1.61	1.02	0.659	0.368
Uncharacterized protein	A0A1D8PP27	CAALFM_C504890CA	orf19	3.22	2.04	0.659	0.368
RRM domain-containing protein	Q59T93	CAALFM_C101850CA	orf19	3.22	2.04	0.659	0.368
Ribulose-phosphate 3-epimerase	A0A1D8PQI4	CAALFM_C700150WA	orf19	22.09	13.99	0.659	0.369
Nucleoporin	A0A1D8PNP6	CAALFM_C503550WA	orf19	27.06	17.09	0.663	0.369
Tfs1p	Q5A1M1	CAALFM_C500930CA	TFS1	67.61	42.7	0.663	0.369
Uncharacterized protein	A0A1D8PFM7	CAALFM_C113320CA	orf19	8.06	5.09	0.663	0.369
Uncharacterized protein	Q5A924	CAALFM_C101220CA	orf19	13.69	8.64	0.664	0.370
Glyoxalase 3	Q5AF03	CAALFM_C302610CA	GLX3	140.03	88.18	0.667	0.370
Thioredoxin peroxidase	Q5A7P9	CAALFM_C300480CA	DOT5	52.66	33.08	0.671	0.371
Uncharacterized protein	Q5AGY4	CAALFM_C701700WA	orf19	6.49	4.07	0.673	0.372
Transcriptional regulator CBF1	Q5A1E3	CAALFM_C406580WA	CBF1	9.75	6.11	0.674	0.372
Cytochrome c oxidase subunit	A0A1D8PQD5	CAALFM_C604250WA	orf19	10.04	6.29	0.675	0.372
Uncharacterized protein	A0A1D8PTW2	CAALFM_CR09140CA	CAALFM_CR09140CA	37.86	23.71	0.675	0.372
DNA-directed RNA polymerase III subunit	A0A1D8PS98	CAALFM_CR02720CA	RPC31	4.09	2.56	0.676	0.372
Cytochrome c oxidase-assembly factor COX23, mitochondrial	Q5A884	CAALFM_CR01100CA	COX23	4.09	2.56	0.676	0.372
Sedoheptulose-bisphosphatase	A0A1D8PHW0	CAALFM_C207420WA	orf19	1.55	0.97	0.676	0.372
NADH dehydrogenase [ubiquinone] iron-sulfur protein 4, mitochondrial	A0A1D8PCD3	CAALFM_C101010WA	orf19	26.58	16.59	0.680	0.373
Translation initiation factor eIF1	Q59LQ6	CAALFM_C504090CA	SUI1	8.42	5.24	0.684	0.374
Ribosomal 60S subunit protein L17B	Q59TE0	CAALFM_C204600CA	RPL17B	54.08	33.63	0.685	0.374
Uso6p	A0A1D8PK69	CAALFM_C305310WA	USO6	39.4	24.48	0.687	0.375
JmjC domain-containing protein	A0A1D8PN39	CAALFM_C501090CA	orf19	1.53	0.95	0.688	0.375
Pre-mRNA polyadenylation factor FIP1	Q5AGC1	CAALFM_C502480WA	FIP1	7.37	4.57	0.690	0.375
Monothiol glutaredoxin	Q5AF81	CAALFM_C402710CA	GRX3	6.31	3.89	0.698	0.377
Chitinase 2	P40953	CAALFM_C504130CA	CHT2	17.87	11	0.700	0.378
Mitochondrial 54S ribosomal protein YmL27	Q5AHY8	CAALFM_C103650CA	MRPL27	10.32	6.35	0.701	0.378
Uncharacterized protein	A0A1D8PJR3	CAALFM_C303340CA	orf19	3.16	1.94	0.704	0.379
Negative cofactor 2 transcription regulator complex subunit	A0A1D8PLM5	CAALFM_C402630CA	HFL2	1.55	0.95	0.706	0.379
Aqy1p	Q5A1Z4	CAALFM_CR02920CA	AQY1	1.55	0.95	0.706	0.379
Heat shock protein 60, mitochondrial	O74261	CAALFM_CR06490CA	HSP60	346.17	212	0.707	0.379
Tyrosine protein phosphatase	A0A1D8PE91	CAALFM_C108260CA	LTP1	3.07	1.88	0.708	0.379
Galactose-1-phosphate uridylyltransferase	A0A1D8PCM2	CAALFM_C102180WA	GAL7	9.13	5.59	0.708	0.380
MICOS complex subunit	Q5A2C6	CAALFM_C105490CA	orf19	6.66	4.07	0.711	0.380
Glucoamylase 1	O74254	CAALFM_C110290WA	GAM1	88.85	54.29	0.711	0.380
Probable electron transfer flavoprotein subunit alpha	A0A1D8PQF9	CAALFM_C604560WA	orf19	18.36	11.2	0.713	0.381
Glycogenin glucosyltransferase	A0A1D8PCG4	CAALFM_C101360CA	orf19	44.5	27.01	0.720	0.382
Tropomyosin	A0A1D8PTR7	CAALFM_CR08460WA	TPM2	30.26	18.35	0.722	0.383
Isoleucine biosynthesis protein	A0A1D8PDX5	CAALFM_C106900CA	MMD1	27.89	16.91	0.722	0.383
Uncharacterized protein	Q5A4Y4	CAALFM_C500310CA	orf19	12.09	7.33	0.722	0.383
RRM domain-containing protein	A0A1D8PET7	CAALFM_C110190WA	orf19	8.5	5.15	0.723	0.383
Putative ammonium permease	A0A1D8PHP8	CAALFM_C206680WA	FRP3	5.62	3.39	0.729	0.384
Uncharacterized protein	A0A1D8PQH1	CAALFM_C700070CA	orf19	15.26	9.2	0.730	0.385
Gir2p	Q5AGV1	CAALFM_C701420WA	GIR2	3.22	1.94	0.731	0.385
Peptide hydrolase	A0A1D8PI95	CAALFM_C208800CA	APE3	41.37	24.91	0.732	0.385
Lysophospholipase	A0A1D8PU17	CAALFM_CR09690CA	PLB3	16.05	9.65	0.734	0.385
Mitochondrial 54S ribosomal protein YmL36	Q59YH5	CAALFM_C501700WA	MRPL36	11.86	7.1	0.740	0.387
Repressed by EFG1 protein 1	Q59ZX3	CAALFM_C114120CA	RBE1	21.51	12.83	0.746	0.388
FG-nucleoporin	A0A1D8PKH4	CAALFM_C306440WA	orf19	18.78	11.2	0.746	0.388
Prefoldin subunit 3	A0A1D8PK48	CAALFM_C305120CA	orf19	5.12	3.05	0.747	0.388
Secreted beta-glucosidase SUN41	Q59NP5	CAALFM_C600820WA	SUN41	5.12	3.05	0.747	0.388
Protein transport protein sec16	A0A1D8PNM2	CAALFM_C503140CA	orf19	5.69	3.37	0.756	0.390
Uncharacterized protein	A0A1D8PSW7	CAALFM_CR05030WA	CAALFM_CR05030WA	2.57	1.52	0.758	0.391
RNA-binding signal recognition particle subunit	A0A1D8PLY2	CAALFM_C403930CA	orf19	8.11	4.79	0.760	0.391
FACT complex subunit SPT16	Q5A1D5	CAALFM_C406500WA	CDC68	2.61	1.54	0.761	0.391
Serine/threonine-protein kinase CST20	P0CY24	CAALFM_C502340CA	CST20	5.22	3.07	0.766	0.393
Uncharacterized protein	A0A1D8PDN8	CAALFM_C105950CA	orf19	2.63	1.54	0.772	0.394
Uncharacterized protein	A0A1D8PTH5	CAALFM_CR07340CA	CAALFM_CR07340CA	2.63	1.54	0.772	0.394

Ribosomal protein P2A	A0A1D8PTS0	CAALFM_CR08360CA	RPP2A	106.98	62.57	0.774	0.394
Long-chain fatty acid-CoA ligase	A0A1D8PU56	CAALFM_CR10160WA	FAA4	6.16	3.6	0.775	0.395
Dos2p	Q5AKU7	CAALFM_C113920WA	DOS2	11.32	6.61	0.776	0.395
Cofilin	A0A1D8PMW6	CAALFM_C500370WA	COF1	211.95	123.35	0.781	0.396
E2 ubiquitin-conjugating protein	Q5A513	CAALFM_C500560WA	orf19	4.13	2.4	0.783	0.396
Vrp1p	A0A1D8PI26	CAALFM_C207930CA	VRP1	11.38	6.61	0.784	0.397
Ribosome biogenesis regulatory protein	A0A1D8PCC8	CAALFM_C100900WA	RRS1	5.41	3.14	0.785	0.397
Rtg1p	Q5APP3	CAALFM_C108640WA	RTG1	6.16	3.57	0.787	0.397
Wh11p	A0A1D8PHF8	CAALFM_C205180WA	WH11	6.19	3.58	0.790	0.398
Transcriptional regulator HMO1	Q59PR9	CAALFM_CR05670CA	HMO1	11.78	6.81	0.791	0.398
RRM domain-containing protein	Q5AK88	CAALFM_C504470CA	orf19	57.49	33.23	0.791	0.398
Putative zinc metalloprotease	A0A1D8PQI1	CAALFM_C700170WA	VPS70	20.52	11.74	0.806	0.401
1,3-beta-glucanosyltransferase PGA4	Q5AJY5	CAALFM_C505390CA	PGA4	21.09	12.05	0.808	0.402
DNA-directed RNA polymerase core subunit	Q59LT9	CAALFM_CR02520WA	RPC19	2.7	1.54	0.810	0.402
Dithiol glutaredoxin	Q5ABB1	CAALFM_C100490CA	TTR1	4.21	2.4	0.811	0.403
AMPK1_CBM domain-containing protein	A0A1D8PCL8	CAALFM_C101930WA	orf19	18.4	10.44	0.818	0.404
Transcriptional regulator GZF3	Q5A201	CAALFM_C02850CA	GZF3	6.49	3.67	0.822	0.405
Long-chain fatty acid transporter	A0A1D8PQN3	CAALFM_C700750WA	ACB1	11.78	6.65	0.825	0.406
Hsp90 cochaperone	A0A1D8PN90	CAALFM_C501820WA	STI1	76.8	43.24	0.829	0.407
Cap-associated protein CAF20	Q5AQ12	CAALFM_C109350WA	CAF20	18.89	10.62	0.831	0.407
Ent3p	A0A1D8PGL6	CAALFM_C202340CA	ENT3	7.66	4.3	0.833	0.408
Iwr1 domain-containing protein	Q5AH41	CAALFM_C702170CA	orf19	8.69	4.86	0.838	0.409
Hgt7p	A0A1D8PG81	CAALFM_C201000WA	HGT7	7.64	4.25	0.846	0.411
Slk19p	Q5ADT0	CAALFM_C307310CA	SLK19	226.01	125.41	0.850	0.411
TPR_REGION domain-containing protein	Q5A9E7	CAALFM_CR01350CA	CAALFM_CR01350CA	11.03	6.11	0.852	0.412
Translation initiation factor eIF5	A0A1D8PNF5	CAALFM_C502490CA	TIF5	9.19	5.09	0.852	0.412
Nbp2p	Q5AGV0	CAALFM_C701410CA	NBP2	4.66	2.56	0.864	0.415
Uncharacterized protein	Q5AAW4	CAALFM_C107080WA	orf19	7.66	4.19	0.870	0.416
Stress response protein NST1	Q5A2K0	CAALFM_C207740WA	NST1	3.74	2.04	0.875	0.417
Cyb2p	Q5AKX8	CAALFM_C113630WA	CYB2	3.74	2.04	0.875	0.417
Uncharacterized protein	Q5A4Z5	CAALFM_C500390CA	orf19	5.77	3.14	0.878	0.418
Uncharacterized protein	A0A1D8PMD5	CAALFM_C405610CA	orf19	53.22	28.96	0.878	0.418
Uncharacterized protein	A0A1D8PC73	CAALFM_C100450CA	orf19	4.71	2.56	0.880	0.418
Chromatin-binding transcription coactivator	Q59UQ4	CAALFM_C400390WA	orf19	11.25	6.11	0.881	0.418
40S ribosomal protein S21	A0A1D8PCG7	CAALFM_C101370CA	RPS21B	311.53	169.08	0.882	0.419
Uncharacterized protein	Q5AMR6	CAALFM_C402110WA	orf19	5.62	3.05	0.882	0.419
Het1p	A0A1D8PPA5	CAALFM_C600100CA	HET1	11.28	6.11	0.885	0.419
Protein phosphatase regulator	A0A1D8PGA2	CAALFM_C201200CA	REG1	46.06	24.91	0.887	0.420
Uncharacterized protein	A0A1D8PL49	CAALFM_C400840WA	orf19	7.87	4.25	0.889	0.420
Type 2C protein phosphatase	A0A1D8PRZ8	CAALFM_CR01520WA	PTC2	4.71	2.53	0.897	0.422
Ecm1p	A0A1D8PLZ5	CAALFM_C404100CA	ECM1	2.61	1.4	0.899	0.422
Protein transport protein SEC9	Q59XP0	CAALFM_C601100WA	SEC9	17.06	9.12	0.904	0.423
Uncharacterized protein	A0A1D8PU62	CAALFM_CR10280WA	CAALFM_CR10280WA	6.44	3.44	0.905	0.424
Phosphate transporter	Q5AMP8	CAALFM_C401940WA	PHO89	8.71	4.65	0.905	0.424
Heat shock protein SSC1, mitochondrial	P83784	CAALFM_C207380WA	SSC1	161.35	85.97	0.908	0.424
Nascent polypeptide-associated complex subunit alpha	Q5ANP2	CAALFM_C304140CA	EGD2	73.58	39.2	0.909	0.425
Hsp70 family ATPase	A0A1D8PG96	CAALFM_C201120WA	KAR2	74.77	39.76	0.911	0.425
Rsn1p	Q5AEC1	CAALFM_C303490WA	RSN1	3.59	1.9	0.918	0.427
Ribosomal 40S subunit protein S15	A0A1D8PK22	CAALFM_C304670CA	RPS15	54.17	28.51	0.926	0.428
Uncharacterized protein	A0A1D8PQN6	CAALFM_C700830CA	orf19	22.49	11.7	0.943	0.432
Bromodomain-containing factor 1	Q5A4W8	CAALFM_C500200CA	BDF1	13.68	7.11	0.944	0.433
TRAMP complex RNA-binding subunit	Q5APC1	CAALFM_C109790CA	orf19	5.62	2.92	0.945	0.433
Nitrogen-responsive transcriptional regulator	A0A1D8PNX9	CAALFM_C504280CA	GLN3	10.73	5.57	0.946	0.433
Ribosomal protein L19	A0A1D8PK40	CAALFM_C304500CA	RPL19A	70.7	36.7	0.946	0.433
Protein phosphatase	A0A1D8PL06	CAALFM_C400340WA	PTC7	6.84	3.55	0.946	0.433
Respiratory growth induced protein 1	Q59KG2	CAALFM_C208290CA	RGI1	122.16	63.22	0.950	0.434
Carboxypeptidase	A0A1D8PDM9	CAALFM_C105770CA	PRC3	3.04	1.57	0.953	0.435
Phosphoribosylaminoimidazolesuccinocarboxamide synthase	A0A1D8PRQ1	CAALFM_CR00510CA	ADE1	10.87	5.61	0.954	0.435
DNA-directed RNA polymerase II subunit	A0A1D8PH80	CAALFM_C204650CA	RPB4	9.43	4.86	0.956	0.435
Mitochondrial 54S ribosomal protein YmL44	A0A1D8PSW9	CAALFM_CR05150WA	CAALFM_CR05150WA	3.78	1.94	0.962	0.437
Uncharacterized protein	Q5ADW1	CAALFM_C307600WA	orf19	5.69	2.92	0.963	0.437
Uncharacterized protein	Q5A6L7	CAALFM_CR04460CA	CAALFM_CR04460CA	1.02	0.52	0.972	0.439
Glucan 1,3-beta-glucosidase 2	Q5AIA1	CAALFM_C102630CA	EXG2	1.02	0.52	0.972	0.439
Ribosomal 60S subunit protein L24A	Q5A6A1	CAALFM_C404890CA	RPL24A	37.33	18.99	0.975	0.439
Uncharacterized protein	A0A1D8PLW5	CAALFM_C403690CA	orf19	2.05	1.04	0.979	0.440
rRNA-binding ribosome biosynthesis protein	A0A1D8PQN7	CAALFM_C700690WA	NOP15	12.1	6.11	0.986	0.442
Glyoxylate reductase	A0A1D8PCW3	CAALFM_C102980WA	GOR1	1.03	0.52	0.986	0.442
Ifm3p	A0A1D8PI81	CAALFM_C208080CA	IFM3	1.03	0.52	0.986	0.442
Phosphatidylinositol 4-phosphate-binding protein	A0A1D8PCX6	CAALFM_C103130CA	GGA2	2.06	1.04	0.986	0.442
CHCH domain-containing protein	A0A1D8PJ94	CAALFM_C301780CA	orf19	1.05	0.53	0.986	0.442
Elf1p	A0A1D8PTY4	CAALFM_CR09370WA	ELF1	1.05	0.53	0.986	0.442
Hsp70 family chaperone	A0A1D8PGU0	CAALFM_C202760WA	LHS1	6.68	3.37	0.987	0.442
Rax2p	A0A1D8PFE5	CAALFM_C112510WA	RAX2	9.64	4.86	0.988	0.442
Transcription initiation factor TFIID subunit 10	Q5A886	CAALFM_CR01120CA	CAALFM_CR01120CA	7.58	3.82	0.989	0.442
Transcription factor TFIIIC subunit	A0A1D8PQW6	CAALFM_C701650WA	orf19	2.03	1.02	0.993	0.443
Protein BCP1	Q59PE7	CAALFM_C112760WA	BCP1	3.09	1.55	0.995	0.444
60S ribosomal protein L13	O59931	CAALFM_C103020CA	RPL13	61.89	30.97	0.999	0.445
Hch1p	A0A1D8PPR5	CAALFM_C601860CA	HCH1	7.16	3.58	1.000	0.445
Uncharacterized protein	A0A1D8PDC7	CAALFM_C104600CA	orf19	7.68	3.84	1.000	0.445
Putative glucan endo-1\3-beta-D-glucosidase	A0A1D8PNW1	CAALFM_C504110WA	SCW11	6.1	3.05	1.000	0.445
Uncharacterized protein	Q59YD2	CAALFM_C209600CA	orf19	12.28	6.11	1.007	0.447
Methylthioribulose-1-phosphate dehydratase	Q5AG73	CAALFM_C502820CA	MDE1	12.28	6.11	1.007	0.447
Ribosome biosynthesis protein	A0A1D8PFH2	CAALFM_C112680WA	orf19	2.05	1.02	1.007	0.447
Uncharacterized protein	A0A1D8PNJ4	CAALFM_C502830WA	orf19	2.05	1.02	1.007	0.447
GATA-type domain-containing protein	Q59TU4	CAALFM_C111690WA	orf19	2.05	1.02	1.007	0.447
Negative cofactor 2 transcription regulator complex subunit	Q5A0I6	CAALFM_C202810CA	NCB2	2.05	1.02	1.007	0.447
RRM domain-containing protein	Q5A297	CAALFM_C105220CA	orf19	2.05	1.02	1.007	0.447
Uncharacterized protein	A0A1D8PQ50	CAALFM_C603370WA	orf19	6.64	3.3	1.009	0.447
Uncharacterized protein	A0A1D8PSQ6	CAALFM_CR04390CA	CAALFM_CR04390CA	13.32	6.61	1.011	0.447
Pheromone-processing carboxypeptidase KEX1	Q5AFP8	CAALFM_C700940WA	KEX1	5.16	2.56	1.011	0.448
Protein kinase	A0A1D8PH10	CAALFM_C203760CA	orf19	1.05	0.52	1.014	0.448
ATPase-activating ribosome biosynthesis protein	Q59SI1	CAALFM_C400510CA	RLP24	1.05	0.52	1.014	0.448
Protein-lysine N-methyltransferase EFM5	Q5A653	CAALFM_C404500CA	EFM5	1.05	0.52	1.014	0.448
Transcription factor IIF subunit	A0A1D8PG13	CAALFM_C200220CA	orf19	2.06	1.02	1.014	0.448
Uncharacterized protein	A0A1D8PQS3	CAALFM_C701070CA	orf19	23.75	11.74	1.017	0.449

Transcriptional regulator SKO1	Q59VR1	CAALFM_C103770WA	SKO1	3.12	1.54	1.019	0.449
Sortilin	A0A1D8PFH5	CAALFM_C112490WA	PEP1	2.07	1.02	1.021	0.450
MFAP1 domain-containing protein	A0A1D8PFJ7	CAALFM_C112800WA	orf19	3.09	1.52	1.024	0.450
Rrp15p	A0A1D8PIJ2	CAALFM_C209380WA	RRP15	7.28	3.58	1.024	0.450
Candidapepsin-10	Q5A651	CAALFM_C404470WA	SAP10	1.02	0.5	1.029	0.451
High-affinity glucose transporter 1	A0A1D8PCL1	CAALFM_C101980WA	HGT1	8.67	4.25	1.029	0.451
Uncharacterized protein	A0A1D8PRB4	CAALFM_C703370CA	orf19	9.72	4.75	1.033	0.452
Altered inheritance of mitochondria protein 21	Q5AP87	CAALFM_C110090CA	AIM21	33.57	16.32	1.041	0.454
Serine/threonine protein kinase	A0A1D8PDY7	CAALFM_C106780WA	NPR1	1.03	0.5	1.043	0.455
Transcriptional regulator of yeast form adherence 2	Q59LX5	CAALFM_C105090WA	TRY2	4.21	2.04	1.045	0.455
Uncharacterized protein	G1UAZ9	CAALFM_C703030WA	orf19	22.08	10.69	1.047	0.455
Ribosome assembly protein 3	Q59VF9	CAALFM_C104710CA	RSA3	2.11	1.02	1.049	0.456
Enhanced filamentous growth protein 1	Q59X67	CAALFM_CR07890WA	EFG1	19.92	9.62	1.050	0.456
Probable electron transfer flavoprotein subunit beta	A0A1D8PTZ6	CAALFM_CR09510CA	CAALFM_CR09510CA	15.97	7.71	1.051	0.456
Uncharacterized protein	A0A1D8PLR3	CAALFM_C403080WA	CAALFM_C403080WA	6.94	3.35	1.051	0.456
Uncharacterized protein	A0A1D8PH99	CAALFM_C204870CA	orf19	1.08	0.52	1.054	0.457
Uncharacterized protein	A0A1D8PT39	CAALFM_CR05890CA	CAALFM_CR05890CA	1.08	0.52	1.054	0.457
Hsp90 cochaperone	A0A1D8PQ94	CAALFM_C603750CA	SBA1	8.56	4.1	1.062	0.459
Pre-mRNA-splicing factor CWC15	Q59PD3	CAALFM_C112630CA	CWC15	1.05	0.5	1.070	0.461
Lysophospholipase	A0A1D8PEB1	CAALFM_C108230CA	PLB5	2.15	1.02	1.076	0.462
Uncharacterized protein	A0A1D8PQC7	CAALFM_C604190CA	orf19	2.15	1.02	1.076	0.462
Hydroxyacylglutathione hydrolase	A0A1D8PID4	CAALFM_C209260CA	GLO2	26.76	12.69	1.076	0.462
Protein-arginine N-methyltransferase	A0A1D8PGT0	CAALFM_C203130WA	orf19	2.16	1.02	1.083	0.464
Fcf2 domain-containing protein	A0A1D8PL04	CAALFM_C307800CA	orf19	2.16	1.02	1.083	0.464
H/ACA ribonucleoprotein complex subunit CBF5	O43101	CAALFM_C110620WA	CBF5	2.16	1.02	1.083	0.464
CAP-Gly domain-containing protein	A0A1D8PSA3	CAALFM_CR02690WA	CAALFM_CR02690WA	2.06	0.97	1.087	0.464
Aspartate-semialdehyde dehydrogenase	Q5ALM0	CAALFM_C202370CA	HOM2	121.76	57.27	1.088	0.465
FG-nucleoporin	A0A1D8PQ58	CAALFM_C603460WA	orf19	15.35	7.19	1.094	0.466
Protein BFR2	Q5ACL9	CAALFM_CR10470CA	BFR2	11.35	5.31	1.096	0.467
NADH-ubiquinone oxidoreductase	Q5A222	CAALFM_CR02620CA	CAALFM_CR02620CA	32.61	15.25	1.097	0.467
Pheromone-regulated membrane protein 10	Q5AH11	CAALFM_C701940CA	PRM10	1.07	0.5	1.098	0.467
Uncharacterized protein	A0A1D8PKJ5	CAALFM_C306610WA	orf19	4.21	1.96	1.103	0.468
Zinc finger-containing protein	Q5A934	CAALFM_C101110CA	ZPR1	33.13	15.42	1.103	0.468
Uncharacterized protein	Q59N81	CAALFM_C503870CA	orf19	15.47	7.19	1.105	0.469
Uncharacterized protein	A0A1D8PGR5	CAALFM_C202540WA	orf19	1.08	0.5	1.111	0.470
Mitochondrial 54S ribosomal protein YmL3	Q5A798	CAALFM_C107910CA	MRPL3	3.07	1.42	1.112	0.470
Clathrin light chain	Q5AMQ2	CAALFM_C401980CA	CLC1	17.85	8.23	1.117	0.471
WW domain-containing protein	A0A1D8PM15	CAALFM_C404330CA	orf19	18.04	8.3	1.120	0.472
Guanylate kinase	A0A1D8PNS0	CAALFM_C503790WA	GUK1	6.62	3.03	1.128	0.474
Signal recognition particle subunit	Q59TA2	CAALFM_C101940CA	orf19	5.27	2.4	1.135	0.475
DNA-directed RNA polymerase III subunit	A0A1D8PLP0	CAALFM_C402800WA	RPC53	3.16	1.42	1.154	0.480
Vps51p	A0A1D8PQ10	CAALFM_C602920CA	VPS51	3.16	1.42	1.154	0.480
Bni4p	A0A1D8PD43	CAALFM_C103860CA	BNI4	10.25	4.59	1.159	0.481
Translation elongation factor 1 subunit beta	A0A1D8PM35	CAALFM_C404480CA	EFB1	218.89	97.63	1.165	0.482
Uncharacterized protein	A0A1D8PGJ8	CAALFM_C202170WA	orf19	5.92	2.63	1.171	0.483
DUF3835 domain-containing protein	A0A1D8PNA9	CAALFM_C502010CA	orf19	27.59	12.25	1.171	0.483
Mitochondrial 37S ribosomal protein PET123	Q59NG6	CAALFM_C600550WA	orf19	18.4	8.16	1.173	0.484
Ribosome biogenesis protein NOP53	A0A1D8PHI7	CAALFM_C205750WA	orf19	4.3	1.9	1.178	0.485
Transcriptional regulator STP4	Q5AH87	CAALFM_C203220CA	STP4	1.02	0.45	1.181	0.486
Kexin	A0A1D8PEG3	CAALFM_C108990CA	KEX2	4.63	2.04	1.182	0.486
Mitochondrial 37S ribosomal protein RSM7	Q5AK02	CAALFM_C505250CA	orf19	4.63	2.04	1.182	0.486
Nrd1 complex RNA-binding subunit	A0A1D8PN05	CAALFM_C500790CA	orf19	1.03	0.45	1.195	0.489
Ribonuclease H	A0A1D8PQ43	CAALFM_C603260WA	orf19	27.88	12.17	1.196	0.489
Transcriptional regulator CRZ1	Q5A4H5	CAALFM_C305780CA	CRZ1	4.61	2.01	1.198	0.489
Mitochondrial 54S ribosomal protein YmL35	Q5AHD3	CAALFM_C203560CA	orf19	8.22	3.58	1.199	0.490
Transcription factor SFL1	Q5A287	CAALFM_CR05990CA	SFL1	4.71	2.04	1.207	0.491
Uncharacterized protein	A0A1D8PSB8	CAALFM_CR02930WA	CAALFM_CR02930WA	15.33	6.63	1.209	0.492
Phosphate-sensing transcription factor	A0A1D8PMD1	CAALFM_C405680WA	PHO4	10.16	4.37	1.217	0.494
Uncharacterized protein	A0A1D8PPV6	CAALFM_C602330WA	orf19	16.63	7.13	1.222	0.495
Putative aspartic endopeptidase	Q5AH56	CAALFM_C702300WA	YPS7	1.05	0.45	1.222	0.495
DNA polymerase epsilon noncatalytic subunit	Q5AHY9	CAALFM_C103640CA	HFL1	1.05	0.45	1.222	0.495
Protein transporter	A0A1D8PLH1	CAALFM_C402140CA	TIM10	4.82	2.06	1.226	0.496
U3 small nucleolar ribonucleoprotein protein MPP10	A0A1D8PFZ9	CAALFM_C200070CA	MPP10	17.37	7.39	1.233	0.497
PI31_Prot_C domain-containing protein	A0A1D8PIK6	CAALFM_C209980WA	orf19	8.42	3.55	1.246	0.500
Xylulokinase	A0A1D8PMB3	CAALFM_C405300WA	XKS1	1.07	0.45	1.250	0.501
40S ribosomal protein S6	A0A1D8PL99	CAALFM_C401270WA	RPS6A	100.21	41.98	1.255	0.502
Rei1p	A0A1D8PDH5	CAALFM_C105060WA	REI1	1.08	0.45	1.263	0.504
Msb2p	A0A1D8PGF8	CAALFM_C201780WA	MSB2	3.66	1.52	1.268	0.505
AP-1-like transcription factor CAP1	Q5AJU7	CAALFM_C302220WA	CAP1	14.97	6.19	1.274	0.506
Hexose transporter	Q5AD47	CAALFM_C201020WA	HGT6	14.83	6.11	1.279	0.508
pH-responsive protein 1	P43076	CAALFM_C404530CA	PHR1	19.71	8.12	1.279	0.508
FG-nucleoporin	A0A1D8PS91	CAALFM_CR02610CA	CAALFM_CR02610CA	6.16	2.53	1.284	0.509
FK506-binding protein 3	Q59VR3	CAALFM_C103790CA	FPR3	19.98	8.15	1.294	0.511
Zds1p	A0A1D8PM39	CAALFM_C404580WA	ZDS1	5.89	2.4	1.295	0.511
Proteasome regulatory particle lid subunit	A0A1D8PNA8	CAALFM_C502030WA	RPN8	18.96	7.65	1.309	0.514
DNA-directed RNA polymerase III subunit	A0A1D8PQS2	CAALFM_C701210CA	orf19	3.8	1.52	1.322	0.517
U5 snRNP complex subunit	A0A1D8PTC5	CAALFM_CR06970CA	CAALFM_CR06970CA	2.55	1.02	1.322	0.517
Glucan 1,3-beta-glucosidase BGL2	Q5AMT2	CAALFM_C402250CA	BGL2	32.16	12.86	1.322	0.517
Dynein light chain	A0A1D8PGE0	CAALFM_C201440CA	orf19	2.63	1.05	1.325	0.518
Gim5p	Q5AA13	CAALFM_C105810WA	GIM5	5.16	2.06	1.325	0.518
Sys3p	A0A1D8PSD4	CAALFM_CR03150WA	SYS3	15.37	6.11	1.331	0.519
Tubulin-specific chaperone A	A0A1D8PST9	CAALFM_CR04650WA	CAALFM_CR04650WA	2.57	1.02	1.333	0.520
Phosducin domain-containing protein	A0A1D8PIN9	CAALFM_C210120WA	orf19	3.77	1.49	1.339	0.521
FG-nucleoporin	A0A1D8PMK3	CAALFM_C406350CA	NUP60	24.05	9.49	1.342	0.522
Transcription initiation factor IIF subunit alpha	Q5AMR1	CAALFM_C402060CA	TFG1	2.59	1.02	1.344	0.522
Uncharacterized protein	Q5AP78	CAALFM_C110170WA	orf19	9.08	3.55	1.355	0.525
Npl6p	A0A1D8PG62	CAALFM_C200710WA	NPL6	2.61	1.02	1.356	0.525
Chromatin modification-related protein EAF6	Q59QC2	CAALFM_C108510WA	EAF6	2.61	1.02	1.356	0.525
Rpd3L histone deacetylase complex subunit	A0A1D8PH30	CAALFM_C204130WA	orf19	8.66	3.37	1.362	0.526
NEDD8 family protein	A0A1D8PJP6	CAALFM_C303320WA	RUB1	2.63	1.02	1.367	0.527
Glycerol-1-phosphatase	Q5A7M9	CAALFM_C300320WA	RHR2	2.63	1.02	1.367	0.527
Adenylate kinase isoenzyme 6 homolog HBR1	Q8TG40	CAALFM_C100340WA	HBR1	12.41	4.79	1.373	0.529
CCR4-NOT core subunit	A0A1D8PE87	CAALFM_C108300WA	NOT5	26.6	10.18	1.386	0.532
PH domain-containing protein	A0A1D8PMG9	CAALFM_C406130WA	orf19	46.33	17.64	1.393	0.533
FG-nucleoporin	A0A1D8PFQ0	CAALFM_C113550CA	NUP49	2.63	1	1.395	0.534

4a-hydroxytetrahydrobiopterin dehydratase	Q5ACY8	CAALFM_C200480CA	PHHB	2.61	0.98	1.413	0.538
Uncharacterized protein	A0A1D8PH72	CAALFM_C204570WA	orf19	12.63	4.72	1.420	0.539
Nucleotide exchange factor SIL1	Q5A360	CAALFM_CR08350WA	SIL1	14.05	5.24	1.423	0.540
MICOS complex subunit MIC10	A0A1D8PM81	CAALFM_C404970CA	orf19	2.55	0.95	1.425	0.540
Uncharacterized protein	A0A1D8PTA8	CAALFM_CR06780WA	CAALFM_CR06780WA	2.69	1	1.428	0.541
Uncharacterized protein	A0A1D8PRS1	CAALFM_CR00690CA	CAALFM_CR00690CA	5.27	1.94	1.442	0.544
Hpc2p	Q5AKU2	CAALFM_C113970CA	HPC2	5.12	1.88	1.445	0.545
Pin3p	Q5ANE7	CAALFM_C304880WA	PIN3	14.72	5.31	1.471	0.551
Mlp1p	A0A1D8PL86	CAALFM_C401060WA	MLP1	34.86	12.57	1.472	0.551
Peroxisomal targeting signal receptor	O74711	CAALFM_C400150CA	PEX5	2.67	0.95	1.491	0.555
Repressor of filamentous growth 1	Q5A220	CAALFM_CR02640WA	RFG1	2.69	0.95	1.502	0.557
Uncharacterized protein	Q59Y36	CAALFM_C208630CA	orf19	16.7	5.83	1.518	0.561
FYVE-type domain-containing protein	A0A1D8PGH2	CAALFM_C201680CA	orf19	6.98	2.4	1.540	0.566
Bromo domain-containing protein	A0A1D8PP04	CAALFM_C504640CA	orf19	1.53	0.52	1.557	0.570
Uncharacterized protein	A0A1D8PPS0	CAALFM_C602020CA	orf19	4.63	1.57	1.560	0.571
Prefolding complex chaperone subunit	Q59VX1	CAALFM_C102300WA	orf19	5.63	1.9	1.567	0.572
zf-C2HC5 domain-containing protein	A0A1D8PSF1	CAALFM_CR03330WA	CAALFM_CR03330WA	3.07	1.02	1.590	0.577
Cap4p	Q5AHD5	CAALFM_C203590CA	CAP4	3.07	1.02	1.590	0.577
Ubiquitin carboxyl-terminal hydrolase	A0A1D8PNY8	CAALFM_C504430CA	YUH2	3.07	1.02	1.590	0.577
Rtf1p	A0A1D8PQN1	CAALFM_C700720WA	RTF1	6.16	2.04	1.594	0.578
Lysophospholipase 1	Q9UWF6	CAALFM_C601990WA	PLB1	6.14	2.01	1.611	0.582
DNA-directed RNA polymerase II core subunit	A0A1D8PCD0	CAALFM_C100940WA	RPB11	1.6	0.52	1.622	0.584
Uncharacterized protein	A0A1D8PCX0	CAALFM_C103100WA	orf19	1.6	0.52	1.622	0.584
Uncharacterized protein	Q5AKW1	CAALFM_C113790CA	orf19	1.61	0.52	1.631	0.586
DNA polymerase epsilon noncatalytic subunit	A0A1D8PG40	CAALFM_C200430CA	DPB4	3.16	1.02	1.631	0.587
Imp2'p	Q5A0W0	CAALFM_C406730CA	orf19	3.16	1.02	1.631	0.587
Myosin 1	A0A1D8PN92	CAALFM_C501650CA	MYO1	1.55	0.5	1.632	0.587
S-adenosylmethionine permease GAP4	Q59WB3	CAALFM_C107120WA	GAP4	1.57	0.5	1.651	0.591
Biofilm and cell wall regulator 1	Q59U10	CAALFM_CR06440CA	BCR1	3.22	1.02	1.659	0.593
Serine/threonine protein kinase	Q5A650	CAALFM_C404460CA	SAK1	3.07	0.97	1.662	0.594
Hap5p	A0A1D8PN26	CAALFM_C500940CA	HAP5	4.88	1.54	1.664	0.594
Rpl7p	Q59V85	CAALFM_CR06120WA	RPL7	7.68	2.4	1.678	0.597
Uncharacterized protein	Q59T87	CAALFM_C101800WA	orf19	6.82	2.1	1.699	0.602
Mitochondrial import inner membrane translocase subunit TIM9	Q59R24	CAALFM_C703630CA	TIM9	6.68	2.04	1.711	0.605
Uncharacterized protein	A0A1D8PTY9	CAALFM_CR09440CA	CAALFM_CR09440CA	6.72	2.04	1.720	0.606
Mediator of RNA polymerase II transcription subunit 11	Q59S43	CAALFM_C701290WA	MED11	4.74	1.42	1.739	0.611
PAPA-1 domain-containing protein	A0A1D8PIC9	CAALFM_C208720WA	orf19	8.56	2.56	1.742	0.611
RRF domain-containing protein	Q5A6S7	CAALFM_CR03950WA	CAALFM_CR03950WA	21.05	6.19	1.766	0.617
Cytidine deaminase	A0A1D8PMQ1	CAALFM_C406860CA	orf19	1.55	0.45	1.784	0.621
Uncharacterized protein	Q5AG39	CAALFM_C503100CA	orf19	5.34	1.54	1.794	0.623
Yeast-form wall Protein 1	Q59Y31	CAALFM_C208590WA	YWP1	44.2	12.74	1.795	0.623
Uncharacterized protein	Q5AKV7	CAALFM_C113820CA	orf19	3.59	1.02	1.815	0.628
54S ribosomal protein L51, mitochondrial	Q5A3J1	CAALFM_C111880WA	MRPL51	1.6	0.45	1.830	0.631
Uncharacterized protein	Q59T47	CAALFM_C205580WA	orf19	7.37	2.01	1.875	0.641
Zcf21p	A0A1D8PL89	CAALFM_C400760WA	ZCF21	5.78	1.57	1.880	0.642
PDCD2_C domain-containing protein	Q5A5B2	CAALFM_C702930CA	orf19	14.94	3.96	1.916	0.650
Nucleosome assembly protein 1	Q5AAI8	CAALFM_CR00320CA	NAP1	7.72	2.04	1.920	0.651
Uncharacterized protein	A0A1D8PU27	CAALFM_CR09800CA	CAALFM_CR09800CA	11.56	3.05	1.922	0.652
Transcription factor IRO1	Q5AJ77	CAALFM_C301360CA	IRO1	7.22	1.88	1.941	0.656
RNA-binding snoRNP assembly protein	A0A1D8PSP5	CAALFM_CR04110WA	CAALFM_CR04110WA	7.9	2.04	1.953	0.659
Uncharacterized protein	A0A1D8PNB9	CAALFM_C501980CA	orf19	3.68	0.95	1.954	0.659
Transcriptional regulator	A0A1D8PCC3	CAALFM_C100930CA	SIN3	2.05	0.52	1.979	0.665
Uncharacterized protein	A0A1D8PH93	CAALFM_C204490WA	orf19	5.88	1.49	1.981	0.665
rRNA-processing protein	A0A1D8PLA3	CAALFM_C401450WA	PWP1	21.01	5.2	2.015	0.672
Arginase	A0A1D8PP00	CAALFM_C504490CA	CAR1	17.87	4.41	2.019	0.673
Uncharacterized protein	Q5A217	CAALFM_CR02670CA	CAALFM_CR02670CA	6.27	1.54	2.026	0.675
Transcriptional regulator IFH1	Q5AG97	CAALFM_C502650CA	IFH1	2.05	0.5	2.036	0.677
Transcription activator MSS11	Q59N20	CAALFM_CR04840CA	MSS11	2.16	0.52	2.054	0.681

Mitochondrial 54S ribosomal protein YmL40	Q5A6S0	CAALFM_CR04010CA	MRPL40	8.67	2.04	2.088	0.689
Lysophospholipase	A0A1D8PGF0	CAALFM_C201380WA	PLB4	22.53	5.29	2.091	0.690
Vacuolar-sorting protein SNF7	Q5ABD0	CAALFM_C100650CA	SNF7	4.21	0.97	2.118	0.696
SHNi-TPR domain-containing protein	A0A1D8PDB1	CAALFM_C104490WA	orf19	13.68	3.08	2.151	0.703
Ribosomal 60S subunit protein L43A	A0A1D8PP14	CAALFM_C504590CA	RPL43A	89.3	19.98	2.160	0.705
U2 snRNP complex subunit	A0A1D8PU52	CAALFM_CR10060WA	CAALFM_CR10060WA	2.05	0.45	2.188	0.711
Virulence protein SSD1	Q5AK62	CAALFM_C504730CA	SSD1	2.06	0.45	2.195	0.713
NMT1 domain-containing protein	Q59X88	CAALFM_CR08050CA	CAALFM_CR08050CA	2.1	0.45	2.222	0.719
Cytokin_check_N domain-containing protein	A0A1D8PJL3	CAALFM_C302760CA	orf19	4.33	0.9	2.266	0.729
Cell wall acid trehalase ATC1	Q5AAU5	CAALFM_C106940CA	ATC1	2.57	0.52	2.305	0.738
Ltv1p	A0A1D8PUB0	CAALFM_CR10650WA	LTV1	5.22	1.05	2.314	0.740
Stress-responsive transcriptional activator	A0A1D8PEH3	CAALFM_C108940CA	MSN4	5.16	1.02	2.339	0.745
Tco89p	A0A1D8PDD8	CAALFM_C104810WA	TCO89	7.37	1.45	2.346	0.747
TMF_TATA_bd domain-containing protein	Q5ADY0	CAALFM_C307740WA	orf19	7.18	1.4	2.359	0.750
Uncharacterized protein	Q5A460	CAALFM_C405650WA	orf19	2.57	0.5	2.362	0.750
Nif3p	A0A1D8PMG0	CAALFM_C405960WA	NIF3	2.63	0.5	2.395	0.758
Uncharacterized protein	A0A1D8PT83	CAALFM_CR06510WA	CAALFM_CR06510WA	2.72	0.5	2.444	0.769
Uso1p	A0A1D8PMA2	CAALFM_C405280WA	USO1	5.26	0.95	2.469	0.774
Virulence factor CaO19.6688	Q59R32	CAALFM_C703560WA	CAALFM_C703560WA	5.64	1	2.496	0.780
Regulator of rDNA transcription 14	Q5AJ85	CAALFM_C301430WA	RRT14	2.55	0.45	2.503	0.782
Rtg3p	A0A1D8PF20	CAALFM_C110990CA	RTG3	6.14	1.02	2.590	0.801
RNA polymerase II subunit A C-terminal domain phosphatase	Q59UG2	CAALFM_CR03210CA	SSU72	3.16	0.52	2.603	0.805
RNA-binding GTPase	A0A1D8PMJ1	CAALFM_C406210CA	orf19	3.59	0.5	2.844	0.858
Prn4p	Q5AA23	CAALFM_C105880WA	PRN4	3.6	0.45	3.000	0.893
Uncharacterized protein	A0A1D8PDN5	CAALFM_C105900WA	orf19	3.71	0.45	3.043	0.903
Uncharacterized protein	A0A1D8PI53	CAALFM_C208450WA	orf19	4.16	0.5	3.057	0.906
FG-nucleoporin	A0A1D8PLY0	CAALFM_C403850WA	orf19	14.34	1.52	3.238	0.947
Uncharacterized protein	A0A1D8PH73	CAALFM_C204620WA	orf19	9.21	0.97	3.247	0.949
Uncharacterized protein	Q5A2T4	CAALFM_C208020CA	orf19	5.12	0.5	3.356	0.973
Uncharacterized protein	A0A1D8PRN1	CAALFM_CR00290WA	CAALFM_CR00290WA	6.14	0.45	3.770	1.066
SWI5-dependent HO expression protein 3	Q5ABV6	CAALFM_C603100WA	SHE3	8.69	0	drug inhibited	2.463
Peptidyl-prolyl cis-trans isomerase	Q59KZ2	CAALFM_C104410CA	ESS1	6.84	0	drug inhibited	2.463
Transcription factor TFIIIB subunit	Q5A9D7	CAALFM_CR01420WA	CAALFM_CR01420WA	3.69	0	drug inhibited	2.463
Purine nucleoside permease	Q5AGW8	CAALFM_C701560CA	NUP	3.6	0	drug inhibited	2.463
U6 snRNA-associated Sm-like protein LSm8	A0A1D8PNJ9	CAALFM_C502810WA	LSM8	3.57	0	drug inhibited	2.463
Mitochondrial 37S ribosomal protein MRP21	Q5A1Z1	CAALFM_CR02950CA	CAALFM_CR02950CA	3.15	0	drug inhibited	2.463
Cic1p	A0A1D8PPJ1	CAALFM_C601170WA	CIC1	3.07	0	drug inhibited	2.463

Pre-rRNA-processing protein ESF2	Q59YL9	CAALFM_C502070CA	ESF2	3.07	0	drug inhibited	2.463
Uncharacterized protein	A0A1D8PFW3	CAALFM_C114240WA	orf19	2.61	0	drug inhibited	2.463
U4/U6-U5 snRNP complex subunit	A0A1D8PNL9	CAALFM_C503010WA	orf19	2.59	0	drug inhibited	2.463
Stb3p	Q59SE2	CAALFM_C209010WA	STB3	2.57	0	drug inhibited	2.463
Uncharacterized protein	A0A1D8PPE1	CAALFM_C600360CA	orf19	2.55	0	drug inhibited	2.463
OTU domain-containing protein	Q5A1N7	CAALFM_C501110WA	orf19	2.55	0	drug inhibited	2.463
Hms1p	A0A1D8PMZ4	CAALFM_C500670CA	HMS1	2.15	0	drug inhibited	2.463
Uncharacterized protein	Q5AMK4	CAALFM_C401430CA	orf19	2.11	0	drug inhibited	2.463
Thioredoxin domain-containing protein	A0A1D8PJ36	CAALFM_C301140WA	orf19	2.1	0	drug inhibited	2.463
Uncharacterized protein	Q5AI68	CAALFM_C102940CA	orf19	2.07	0	drug inhibited	2.463
Spa2p	A0A1D8PFH4	CAALFM_C112620WA	SPA2	2.05	0	drug inhibited	2.463
Uncharacterized protein	Q59T43	CAALFM_C205640WA	orf19	2.05	0	drug inhibited	2.463
SWR1-complex protein 5	Q5A8H7	CAALFM_C603950CA	SWC5	2.05	0	drug inhibited	2.463
Uncharacterized protein	A0A1D8PLA2	CAALFM_C401420WA	orf19	1.6	0	drug inhibited	2.463
Serine/threonine-protein phosphatase 2A 56 kDa regulatory subunit	Q59Q44	CAALFM_C206420CA	RTS1	1.6	0	drug inhibited	2.463
GCR1_C domain-containing protein	Q59U54	CAALFM_C207370WA	orf19	1.6	0	drug inhibited	2.463
Na+)-exporting P-type ATPase	Q5ABA1	CAALFM_C100390WA	ENA2	1.57	0	drug inhibited	2.463
ANK_REP_REGION domain-containing protein	A0A1D8PEE1	CAALFM_C108700WA	orf19	1.56	0	drug inhibited	2.463
Ribosome biosynthesis protein	A0A1D8PI86	CAALFM_C208180CA	orf19	1.56	0	drug inhibited	2.463
Polyphosphatase	Q5AGF0	CAALFM_C502220CA	orf19	1.55	0	drug inhibited	2.463
Hap42p	A0A1D8PGE6	CAALFM_C201700CA	HAP42	1.55	0	drug inhibited	2.463
Histone acetyltransferase	Q5AI28	CAALFM_C103290WA	NGG1	1.55	0	drug inhibited	2.463
Heat shock transcription factor	Q5AQ33	CAALFM_C109170WA	CTA8	1.55	0	drug inhibited	2.463
Uncharacterized protein	A0A1D8PQC6	CAALFM_C604140CA	orf19	1.53	0	drug inhibited	2.463
VHS domain-containing protein	A0A1D8PIJ4	CAALFM_C209710CA	orf19	1.52	0	drug inhibited	2.463
PWWP domain-containing protein	A0A1D8PQR7	CAALFM_C700910CA	orf19	1.52	0	drug inhibited	2.463
PWWP domain-containing protein	Q5A4L3	CAALFM_C601590WA	orf19	1.52	0	drug inhibited	2.463
Divalent metal ion transporter	A0A1D8PHV2	CAALFM_C207160WA	SMF12	1.08	0	drug inhibited	2.463
Bem3p	A0A1D8PLJ2	CAALFM_C402310WA	BEM3	1.08	0	drug inhibited	2.463
Uncharacterized protein	A0A1D8PLW9	CAALFM_C403770WA	orf19	1.08	0	drug inhibited	2.463
Transcription elongation factor 1 homolog	Q59PU1	CAALFM_CR05910WA	CAALFM_CR05910WA	1.08	0	drug inhibited	2.463
Putative mitochondrial 37S ribosomal protein SWS2	Q5A357	CAALFM_CR08400CA	CAALFM_CR08400CA	1.08	0	drug inhibited	2.463
Fcr1p	Q5ADM4	CAALFM_C306850WA	FCR1	1.08	0	drug inhibited	2.463
Uncharacterized protein	Q5ANH4	CAALFM_C304690CA	orf19	1.08	0	drug inhibited	2.463
Uncharacterized protein	A0A1D8PPC2	CAALFM_C600400CA	orf19	1.07	0	drug inhibited	2.463
EKC/KEOPS complex subunit GON7	Q59W04	CAALFM_C102310CA	GON7	1.07	0	drug inhibited	2.463
Uncharacterized protein	A0A1D8PRN5	CAALFM_CR00160CA	CAALFM_CR00160CA	1.05	0	drug inhibited	2.463
G-patch_2 domain-containing protein	A0A1D8PU82	CAALFM_CR10430CA	CAALFM_CR10430CA	1.05	0	drug inhibited	2.463
C2H2-type domain-containing protein	A0A1D8PCR9	CAALFM_C102450CA	orf19	1.05	0	drug inhibited	2.463
EAF domain-containing protein	A0A1D8PFG3	CAALFM_C112710CA	orf19	1.05	0	drug inhibited	2.463
Protein phosphatase regulator	A0A1D8PHB1	CAALFM_C205260WA	BUD14	1.05	0	drug inhibited	2.463
DNL-type domain-containing protein	A0A1D8PQU2	CAALFM_C701360CA	orf19	1.05	0	drug inhibited	2.463
Cch1p	Q5A936	CAALFM_C101100WA	CCH1	1.05	0	drug inhibited	2.463
Glutamate synthase	A0A1D8PDU9	CAALFM_C106550WA	GLT1	1.04	0	drug inhibited	2.463
Uncharacterized protein	A0A1D8PNU5	CAALFM_C503980WA	orf19	1.03	0	drug inhibited	2.463
2-methoxy-6-polyprenyl-1,4-benzoquinol methylase, mitochondrial	Q59ZE2	CAALFM_C205470WA	COQ5	1.03	0	drug inhibited	2.463
Rca1p	Q5AB69	CAALFM_C100080CA	RCA1	1.03	0	drug inhibited	2.463
Uncharacterized protein	A0A1D8PDP5	CAALFM_C105790WA	orf19	1.02	0	drug inhibited	2.463
Ubiquinol--cytochrome-c reductase subunit 10	A0A1D8PDP8	CAALFM_C106050CA	orf19	1.02	0	drug inhibited	2.463
Uncharacterized protein	A0A1D8PNC3	CAALFM_C502060WA	orf19	1.02	0	drug inhibited	2.463
TFIID_20kDa domain-containing protein	A0A1D8PSN1	CAALFM_CR03910CA	TAF12L	1.02	0	drug inhibited	2.463
Uncharacterized protein	A0A1D8PTT3	CAALFM_CR08880CA	CAALFM_CR08880CA	1.02	0	drug inhibited	2.463
Transcriptional regulator of filamentous growth FLO8	Q59QW5	CAALFM_C604350CA	FLO8	1.02	0	drug inhibited	2.463
Uncharacterized protein	Q59UG6	CAALFM_CR03250CA	CAALFM_CR03250CA	1.02	0	drug inhibited	2.463
Uncharacterized protein	Q5A327	CAALFM_CR08620CA	CAALFM_CR08620CA	1.02	0	drug inhibited	2.463
Ena21p	Q5A5B0	CAALFM_C702910WA	ENA21	1.02	0	drug inhibited	2.463
Carbohydrate metabolism regulator TYE7	Q5AL36	CAALFM_C113140CA	TYE7	1.02	0	drug inhibited	2.463
Nab3p	A0A1D8PPX7	CAALFM_C602600WA	NAB3	1	0	drug inhibited	2.463
Uncharacterized protein	Q59V65	CAALFM_CR06330CA	CAALFM_CR06330CA	1	0	drug inhibited	2.463
rRNA biogenesis protein RRP36	Q59SN0	CAALFM_CR07030CA	RRP36	0.55	0	drug inhibited	2.463
Ftr2p	A0A1D8PFV2	CAALFM_C114220CA	FTR2	0.53	0	drug inhibited	2.463
Transcription initiation factor TFIID subunit 4	Q59U67	CAALFM_C207460WA	TAF4	0.53	0	drug inhibited	2.463
Cell wall integrity transcriptional regulator CAS5	Q5AMH6	CAALFM_C401190WA	CAS5	0.53	0	drug inhibited	2.463
Uncharacterized protein	A0A1D8PGB8	CAALFM_C201420CA	orf19	0.52	0	drug inhibited	2.463
Mitochondrial 54S ribosomal protein YmL25	A0A1D8PUC5	CAALFM_CR10830CA	CAALFM_CR10830CA	0.52	0	drug inhibited	2.463
Transcription and mRNA export factor SUS1	Q5ADP6	CAALFM_C307050WA	SUS1	0.52	0	drug inhibited	2.463
Uncharacterized protein	Q5AP68	CAALFM_C110250CA	orf19	0.52	0	drug inhibited	2.463
Djp1p	A0A1D8PEX2	CAALFM_C110480WA	DJP1	0.5	0	drug inhibited	2.463
Repressor of RNA polymerase III transcription MAF1	A0A1D8PI21	CAALFM_C208120WA	MAF1	0.5	0	drug inhibited	2.463
Slp2p	A0A1D8PIA6	CAALFM_C209170WA	SLP2	0.5	0	drug inhibited	2.463
Ato1p	A0A1D8PJ22	CAALFM_C300920WA	ATO1	0.5	0	drug inhibited	2.463
Electron transfer flavoprotein-ubiquinone oxidoreductase	A0A1D8PNB3	CAALFM_C501960CA	orf19	0.5	0	drug inhibited	2.463
Uncharacterized protein	A0A1D8PU55	CAALFM_CR10230WA	CAALFM_CR10230WA	0.5	0	drug inhibited	2.463
Duo1p	Q59P88	CAALFM_C404220WA	DUO1	0.5	0	drug inhibited	2.463
Uncharacterized protein	Q59SC0	CAALFM_C208850CA	orf19	0.5	0	drug inhibited	2.463
3'\5'-cyclic-nucleotide phosphodiesterase PDE1	Q5AGE4	CAALFM_C502290WA	PDE1	0.5	0	drug inhibited	2.463
pH-response transcription factor pacC/RIM101	Q9UW14	CAALFM_C114340CA	RIM101	0.5	0	drug inhibited	2.463

Table S3. Fitting parameters for the SAXS patterns of control liposomes and liposomes with PQA-Az-13.

Fitting parameter	Control liposomes	Liposomes with PQA-Az-13
Tail thickness (Å)	11.4 ± 0.003 (PD* = 0.04)	10.7 ± 0.003 (PD* = 0.04)
Head thickness (Å)	10.6 ± 0.007	11.5 ± 0.005
SLD tail (x 10^{-6} Å^{-2})	8.5 (fixed)	8.5 (fixed)
SLD head (x 10^{-6} Å^{-2})	10.5 (fixed)	10.5 (fixed)
SLD solvent (x 10^{-6} Å^{-2})	9.45 (fixed)	9.45 (fixed)

*PD = Gaussian polydispersity

References

1. Lipinski CA, Lombardo F, Dominy BW, Feeney PJ. Experimental and computational approaches to estimate solubility and permeability in drug discovery and development settings. *Adv Drug Deliv Rev*. 2001;46(1-3):3-26. doi: 10.1016/s0169-409x(00)00129-0

2. Daina A, Michielin O, Zoete V. SwissADME: a free web tool to evaluate pharmacokinetics, drug-likeness and medicinal chemistry friendliness of small molecules. *Sci Rep.* 2017;7:42717. doi: 10.1038/srep42717
